# Transcriptomic Analysis of Vibrio parahaemolyticus Underlying the Wrinkly and Smooth Phenotypes

**DOI:** 10.1128/spectrum.02188-22

**Published:** 2022-09-13

**Authors:** Qimin Wu, Xue Li, Tingting Zhang, Miaomiao Zhang, Xingfan Xue, Wenhui Yang, Lingfei Hu, Zhe Yin, Dongsheng Zhou, Yuyu Sun, Renfei Lu, Yiquan Zhang

**Affiliations:** a Department of Clinical Laboratory, Affiliated Nantong Hospital 3 of Nantong University, Nantong, Jiangsu, China; b School of Medicine, Jiangsu University, Zhenjiang, Jiangsu, China; c State Key Laboratory of Pathogen and Biosecurity, Beijing Institute of Microbiology and Epidemiology, Beijing, China; McGill University

**Keywords:** *Vibrio parahaemolyticus*, wrinkly, smooth, biofilm, virulence, regulation

## Abstract

Vibrio parahaemolyticus, a causative agent of seafood-associated gastroenteritis, undergoes opaque-translucent (OP-TR) colony switching associated with capsular polysaccharide (CPS) production. Here, we showed that V. parahaemolyticus was also able to naturally and reversibly switch between wrinkly and smooth phenotypes. More than 1,000 genes were significantly differentially expressed during colony morphology switching, including the major virulence gene loci and key biofilm-related genes. The genes responsible for type III secretion system 1 (T3SS1), type VI secretion systems (T6SS1 and T6SS2), and flagellar synthesis were downregulated in the wrinkly spreader phenotype, whereas genes located on the pathogenicity island Vp-PAI and those responsible for chitin-regulated pili (ChiRP) and Syp exopolysaccharide synthesis were upregulated. In addition, we showed that the wrinkly spreader grew faster, had greater motility and biofilm capacities, and produced more c-di-GMP than the smooth type. A dozen genes potentially associated with c-di-GMP metabolism were shown to be significantly differentially expressed, which may account for the differences in c-di-GMP levels between the two phenotypes. Most importantly, dozens of putative regulators were significantly differentially expressed, and hundreds of noncoding RNAs were detected during colony morphology switching, indicating that phenotype switching is strictly regulated by a complex molecular regulatory network in V. parahaemolyticus. Taken together, the presented work highlighted the gene expression profiles related to wrinkly-smooth switching, showing that the significantly differentially expressed genes were involved in various biological behaviors, including virulence factor production, biofilm formation, metabolism, adaptation, and colonization.

**IMPORTANCE** We showed that Vibrio parahaemolyticus was able to naturally and reversibly switch between wrinkly and smooth phenotypes and disclosed the gene expression profiles related to wrinkly-smooth switching, showing that the significantly differentially expressed genes between the two colony morphology phenotypes were involved in various biological behaviors, including virulence factor production, biofilm formation, metabolism, adaptation, and colonization.

## INTRODUCTION

Vibrio parahaemolyticus is a Gram-negative, halophilic bacterium that can cause seafood-associated gastroenteritis in humans who eat raw or undercooked seafood (1). Full virulence of V. parahaemolyticus requires the production of various virulence determinants, including thermostable direct hemolysin (TDH), TDH-related hemolysin (TRH), type III secretion systems (T3SS1 and T3SS2), type VI secretion systems (T6SS1 and T6SS2), lipopolysaccharide, and extracellular proteases (2, 3). The two hemolysins contribute to the hemolytic activities of V. parahaemolyticus, but only TDH can cause β-type hemolysis on Wagatsuma agar, known as the Kanagawa phenomenon (KP) (4, 5). TDH also contributes to cytotoxicity and enterotoxicity (5). T3SS is a needle-like contractile injection system that injects toxic effectors into host cells to manipulate host cell functioning (6). V. parahaemolyticus T3SS1 predominantly contributes to cytotoxicity against several cell lines, whereas T3SS2 is mainly required for enterotoxicity in animal models (7, 8). Like the T3SS pathway, T6SS is also able to translocate effectors into recipient cells (9). T6SS2 functions as a factor in adherence of V. parahaemolyticus to host cells, whereas T6SS1 mainly contributes to the antibacterial activity of V. parahaemolyticus, which might improve the environmental fitness of the bacterium in natural niches (10, 11).

V. parahaemolyticus is also capable of forming biofilms on surfaces, which is a strategy for the bacterium to adapt to adverse growth conditions (12). Biofilm formation by V. parahaemolyticus is an extremely complex process that requires the participation of various specific structures and substances, such as flagella, type IV pili, exopolysaccharide (EPS), extracellular DNA (eDNA), and extracellular proteins (12–15). Flagella-mediated motility is required for the initial stages of biofilm formation by promoting movement toward and along the surface (12). V. parahaemolyticus possesses two kinds of flagella, a single polar flagellum for swimming and lateral flagella for swarming (16). Polar flagellar mutants of V. parahaemolyticus are unable to form mature biofilms, and the defects cannot be overcome by extending growth periods (15). V. parahaemolyticus produces two kinds of type IV pili, mannose-sensitive hemagglutinin type IV pili (MSHA) and chitin-regulated pili (ChiRP), both of which play roles in biofilm formation but through different mechanisms (14). MSHA contributes to bacterial attachment to the surface, whereas ChiRP is required for bacterial agglutination (14). EPS is the main matrix component of biofilms that helps the bacterial cells attach together on the surface (12). In V. parahaemolyticus, the *cpsA*-*K* and *sypA*-*R* loci have been demonstrated to be responsible for EPS production (12, 17). Expression of these loci leads to the biofilm-associated wrinkly colony morphology, whereas mutations in these loci result in smooth colonies (12, 17). Biofilm formation by V. parahaemolyticus has also been strongly correlated with the extracellular proteins and extracellular DNA concentrations in polymeric substances (13). Biofilm formation by V. parahaemolyticus has been shown to be tightly regulated by numerous factors, including regulators such as OxyR (18), ToxR (19), AphA (20), and OpaR (21); environmental parameters such as temperature (22) and salinity (23); and the ubiquitous secondary messenger bis-(3′–5′)-cyclic di-GMP (c-di-GMP) (12).

V. parahaemolyticus switches between opaque (OP) and translucent (TR) phenotypes based on whether capsular polysaccharide (CPS) is produced or not (17, 24). TR colonies either do not produce CPS or produce less than OP colonies (17). CPS plays a negative role in biofilm formation (25), suggesting that switching between OP and TR colonies is closely related to the biofilm formation ability of V. parahaemolyticus. Indeed, a study showed that both OP and TR strains were able to form biofilms but with different structures (15). Another type of adaptive biofilm-associated switching between wrinkly and smooth colony types has been observed in other *Vibrio* species, such as Vibrio alginolyticus, Vibrio fischeri, and Vibrio cholerae (26–28). Transcriptomic analysis demonstrated that the wrinkly and smooth phenotypes of V. fischeri differed in their expression profiles, showing that genes related to major biochemical cascades, such as those involved in oxidative stress and membrane transport play roles in the wrinkly phenotype (26). At least 124 differentially expressed genes were associated with the wrinkly and smooth variants of V. cholerae, including the *vps* gene loci for EPS production (28). Wild-type V. parahaemolyticus strains can form wrinkly colonies on Congo red plates (12). Some gene mutants of V. parahaemolyticus also show altered colony morphology. For example, mutation of *aphA*, *toxR*, or *hns* leads to smooth colonies on agar plates, whereas *opaR* or *qsvR* mutants were more wrinkled than the wild-type strain (15, 19, 29–31). However, whether wrinkly-smooth colony switching occurs naturally in V. parahaemolyticus remains unknown.

In the present study, we demonstrated that V. parahaemolyticus was able to naturally and reversibly switch between the wrinkly and smooth phenotypes. The results of transcriptomic analysis showed that more than 1,000 genes were significantly differentially expressed between the two phenotypes, including the major virulence gene loci and known key biofilm-related genes. The phenotypic data showed that the wrinkly-type strain grew faster, had greater motility and biofilm formation capacities, and produced more c-di-GMP than the smooth-type strain. These results demonstrated that V. parahaemolyticus may utilize reversible switching from a smooth phenotype to a wrinkly phenotype to alter functions such as biofilm formation to better adapt to changing environments.

## RESULTS AND DISCUSSION

### V. parahaemolyticus switches between wrinkly and smooth phenotypes.

**FIG 1:**
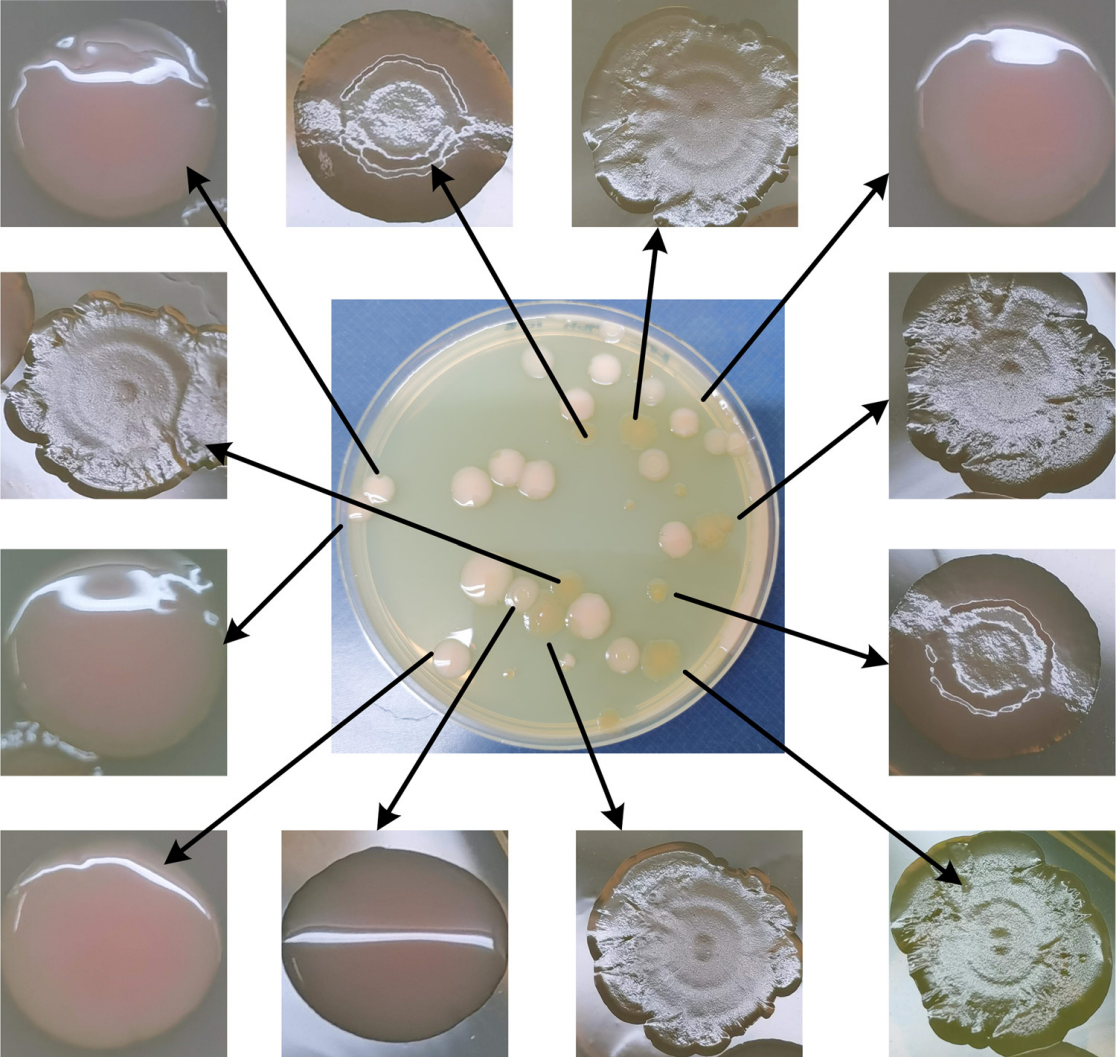
Vibrio parahaemolyticus switches between wrinkly and smooth phenotypes. A monoclonal strain of V. parahaemolyticus was statically incubated in M broth at 30°C for 48 h, transferred to agar plates, and incubated for 48 h at 37°C. The smooth colonies had neat circumferences and possessed smooth, raised, and moist surfaces. In contrast, wrinkly colonies had irregular circumferences and possessed sunken, wrinkled, and slightly dry surfaces.

Spatially heterogeneous niches such as nonshaking test tubes affect the occurrence and maintenance of bacterial morphology diversity (32). Pseudomonas fluorescens exhibits three dominant morphs on agar plates, smooth, wrinkly, and fuzzy colonies (32, 33). The data presented here showed that V. parahaemolyticus was also able to switch between wrinkly and smooth phenotypes on agar plates after being statically cultured in marine (M) broth at 30°C for 48 h (Fig. 1). Most interestingly, purely smooth colonies produced wrinkly colonies and vice versa (data not shown). Although several studies have demonstrated that EPS production or not dictates the wrinkly or smooth phenotype (12, 17, 34), the causes and significance of wrinkly and smooth switching are not fully understood. The wrinkly variation significantly enhances the microbial fitness of V. fischeri in the squid host (26). Thus, we speculated that wrinkly phenotype strains of V. parahaemolyticus may be better able to adapt to adverse environments than smooth phenotype strains. However, more research should be conducted in the future to verify this hypothesis.

### Growth of wrinkly and smooth colonies.

Smooth and wrinkly colonies were randomly selected in triplicate, resuspended in phosphate-buffered saline (PBS) to an optical density at 600 nm (OD_600_) value of 1.4, 50-fold diluted into 10 mL of heart infusion (HI) (or M) broth, and allowed to continuously grow at 37°C (or 30°C) with shaking at 200 rpm. The OD_600_ values of each culture were measured at 1-h intervals to create growth curves. The results showed that wrinkly colonies exhibited higher growth rates than smooth colonies in HI broth at 37°C (see Fig. S1a in the supplemental material). The opposite result was observed when the bacteria were cultured in M broth at 30°C but only in the late-logarithmic growth phase (Fig. S1b). These results suggested that wrinkly spreaders had greater growth advantages than smooth ones in nutrient-rich conditions. According to the growth curves, V. parahaemolyticus strains had longer adaptation periods when transferred from agar plates into liquid broths than when transferred from broth to broth media (19, 35).

### Overview of RNA-seq results.

**FIG 2:**
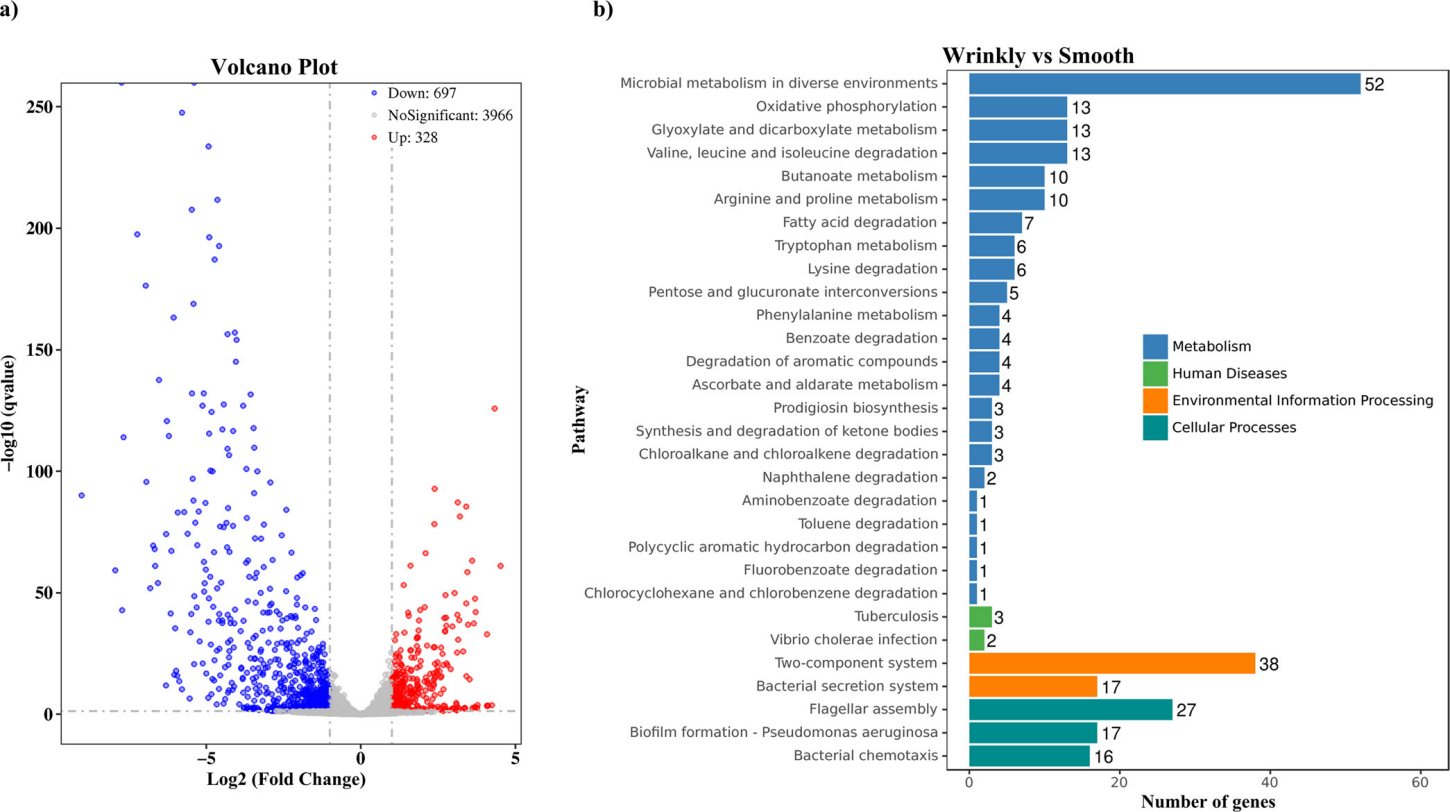
Gene expression of wrinkly and smooth colonies. (a) Volcano plot showing gene expression. Red, blue, and gray points represent upregulated, downregulated, and nonsignificant genes, respectively. (b) Pathways of differentially expressed genes analyzed by Kyoto Encyclopedia of Genes and Genomes (KEGG). The number on the right of each bar indicates the number of differentially expressed genes.

The mRNA profiles of wrinkly V. parahaemolyticus colonies (reference) were compared with those of smooth colonies (test) using RNA sequencing (RNA-seq) assays to investigate the genes responsible for the phenotype switching. We sequenced six Illumina libraries, three from wrinkly colonies and three from smooth colonies, and obtained more than 16.4 million reads for each library, of which more than 96% were uniquely mapped to the genome of V. parahaemolyticus RIMD 2210633. As shown in Fig. 2a, a total of 1,025 genes were significantly differentially expressed in smooth colonies compared to wrinkly colonies, of which 328 were upregulated and 697 were downregulated. The genome of V. parahaemolyticus RIMD 2210633 consists of two chromosomes and contains 4,832 genes (36). Therefore, it can be concluded that the expression of at least one-fifth of all V. parahaemolyticus genes were significantly altered during wrinkly and smooth phenotype switching. The results of Kyoto Encyclopedia of Genes and Genomes (KEGG) analysis showed that 167 genes were involved in metabolism, 60 genes were involved in cellular processes, 55 genes were involved in environmental information processing, and 5 genes were involved in human diseases (Fig. 2b). The functions of differentially expressed genes were predicted using the Cluster of Orthologous Groups of proteins (COG) database, and the results demonstrated that they could be further divided into 20 functional categories as shown in Fig. S2 in the supplemental material. A detailed description of the differentially expressed genes is listed in Table S2 in the supplemental material.

### Validation of RNA-seq data by qPCR.

**FIG 3:**
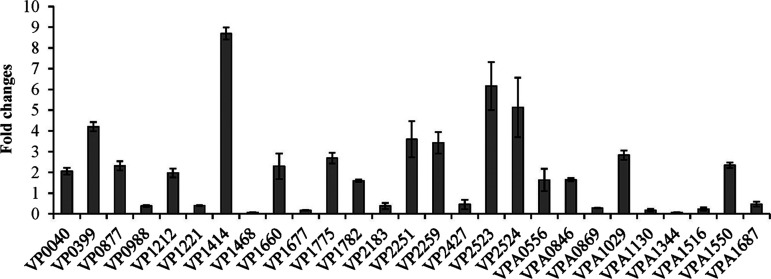
Validation of RNA-seq data by qPCR. The relative mRNA levels of each selected gene were compared between wrinkly and smooth spreaders. The 16S rRNA gene was used as the internal control.

The quantitative PCR (qPCR) was used to validate the RNA-seq data. Twenty-seven genes were selected as target genes (see Table S3 in the supplemental material). As shown in Fig. 3, the qPCR results of all of the tested genes showed a consistent trend with those of RNA-seq (Table S3), confirming the reliability of the transcriptome data.

### Biofilm-associated genes.

**FIG 4:**
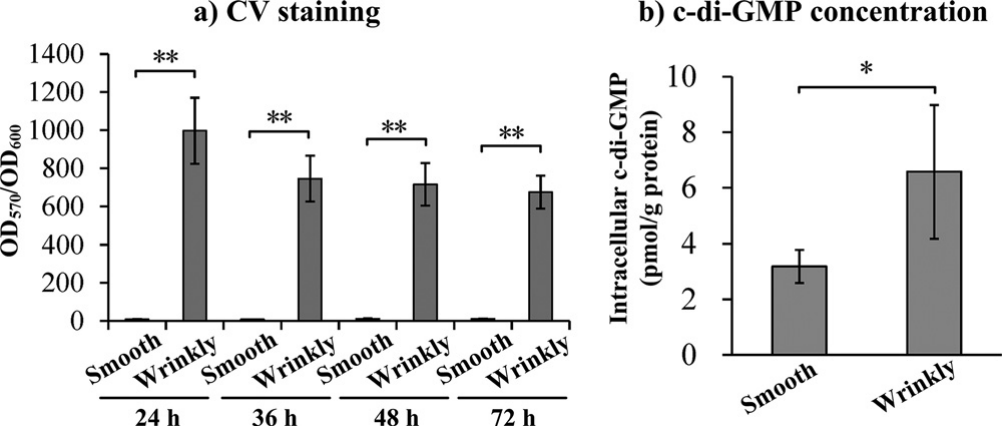
Biofilm-related phenotypic assays. (a) crystal violet staining was used to measure V. parahaemolyticus biofilms grown for different incubation times. (b) Intracellular c-di-GMP concentrations. The data are expressed as the mean ± SD of at least three independent experiments. Asterisks indicate statistically significant differences between wrinkly and smooth colonies (*, *P* < 0.05; **, *P* < 0.01).

Wrinkly colonies of V. fischeri demonstrated a stronger biofilm formation ability than smooth colonies (37). This prompted us to assess the differences in biofilm formation between the two types of V. parahaemolyticus colonies. Unsurprisingly, wrinkly colonies produced significantly more biofilms than smooth colonies at all time points tested (Fig. 4a). The biofilm matrix contains three main substances, EPS, eDNA, and extracellular proteins, and their concentrations change in different ways during biofilm development (13). The *cpsA–K* (VPA1403*–*1413) and *sypA–R* (VP1476*–*1458) operons are responsible for EPS synthesis in V. parahaemolyticus (12, 17). However, only transcripts of VPA1412 (*cpsJ*) and VPA1413 (*cpsK*), but not other genes in the *cps* locus, were able to be detected in samples from the two phenotypes according to RNA-seq data (data not shown). The data also demonstrated similar expression levels of VPA1412 and VPA1413 between the wrinkly and smooth phenotypes. In contrast, expression of nine *syp* genes, including *sypG*, was significantly induced in wrinkly colonies relative to smooth colonies (Table S3). It is unclear why V. parahaemolyticus does not express *cps* genes in both wrinkly and smooth phenotypes. It may be that the RNA samples used in RNA-seq were extracted at particular time points that did not reflect the dynamic expression of genes involved in colony morphology switching. However, the data at least indicated that EPS-related genes may no longer be required in colonies that have completed colony morphology switching. The roles of *syp* genes in host colonization, biofilm formation, and EPS production have been well investigated in V. fischeri (38–41). However, only one study showed that deletion of *sypG* decreased biofilm formation by V. parahaemolyticus. More studies should be performed to investigate the roles of *syp* genes in V. parahaemolyticus.

No differences in expression levels of CPS genes were detected between the two phenotypes (data not shown). Both wrinkly and smooth colonies displayed the OP phenotype on HI agar plates at all time points observed (see Fig. S3 in the supplemental material), suggesting that the two types of adaptive biofilm-associated variations do not occur simultaneously.

Type IV pili have been demonstrated to play positive roles in V. parahaemolyticus biofilm formation (14). ChiRP was shown to be involved in bacterial aggregation, but MSHA is required for bacterial attachment to the surface (14). Our data showed that two ChiRP-associated genes, *pilA* and *pilB*, were significantly repressed in wrinkly colonies compared with those in smooth colonies (Table S3), whereas the VP2693–2707 gene locus responsible for MSHA synthesis was expressed at similar levels in the two phenotypes (data not shown). These results suggested that type IV pili may not play a role in the process of colony morphology switching. In addition, the majority of lateral flagellar genes as well as two polar flagellar genes were induced in wrinkly colonies (Table S3). The results of motility assays also showed that the swimming and swarming capacities of wrinkly colony strains were significantly increased relative to those of smooth colony strains at all time points tested, and the diameter of bacterial motility for both phenotypes increased over time (see Fig. S4 in the supplemental material). These results suggested that the wrinkly phenotype may be better able to search for nutrients or to avoid harmful environments. However, Chavez-Dozal et al. have demonstrated that the expression of genes responsible for flagella biosynthesis in V. fischeri was significantly lower in the wrinkly phenotype and argued that the loss of this trait sped up adaptation (26). Thus, although the phenotypic changes were similar, gene expression profiles may differ based on genetic background.

The c-di-GMP is able to regulate multiple cellular pathways in bacteria, including those related to biofilm formation, motility, and virulence (42). c-di-GMP is synthesized by diguanylate cyclase (DGC), which contains a GGDEF domain, and degraded by phosphodiesterase (PDE), which contains either a HD-GYP or EAL domain (42). At least 50 genes may encode proteins involved in c-di-GMP metabolism in V. parahaemolyticus RIMD 2210633 (36), but only a few of them, including *scrG*, *scrC*, *scrO*, *gefA*, *scrJ*, *scrL*, and *lafV*, have been well investigated and shown to be required for the regulation of motility and biofilm formation (43–47). A total of 15 genes that may encode DGC and/or PDF were significantly differentially expressed in wrinkly colonies relative to smooth colonies (Table S3), among which eight were upregulated (VP0376, VP1979, VP2366, VPA0059, VPA0476, VPA0609, VPA0869, and VPA0927) and seven were downregulated (VP0699, VP1483, VP2979, VPA0556, VPA0818, VPA0846, and VPA1176). The wrinkly phenotype strains contained much higher intracellular c-di-GMP levels than the smooth phenotype strains (Fig. 4b). This is consistent with the fact that c-di-GMP promotes the production of EPS, which is highly present within wrinkly spreaders (17, 42). Future studies should focus on uncovering the roles of these c-di-GMP-associated genes and elucidating how the c-di-GMP signal controls wrinkly-smooth phenotype switching.

### Major virulence factor genes.

The V. parahaemolyticus RIMD 2210633 genome harbors two types of T3SS gene clusters, the T3SS1 (VP1656-VP1702) gene cluster and the T3SS2 (VPA1320-1370) gene cluster (36). The T3SS2 gene cluster together with the *tdh* genes are located on a pathogenicity island known as Vp-PAI (VPA1312–1398) (36). Both T3SS1 and Vp-PAI are required for the full virulence of V. parahaemolyticus (7, 48). A total of 18 genes within the T3SS1 gene cluster were significantly differentially expressed in wrinkly colonies compared with smooth colonies; of these, 14 genes, including the *exsA* gene, were downregulated and four (VP1677, VP1678, VP1679, and VP1685) were upregulated (Table S3). The roles of VP1677, VP1678, VP1679, and VP1685 in wrinkly-smooth phenotype switching are worth investigating in the future. ExsA positively regulates the transcription of T3SS1 genes but negatively regulates lateral flagellar genes (49, 50). However, whether ExsA regulates biofilm formation or plays a role in wrinkly-smooth phenotype switching still needs to be further investigated. The regulatory activity of ExsA is controlled by the ExsCDE cascade (51, 52). ExsD binds ExsA to block ExsA-dependent expression of T3SS1 genes, whereas ExsC binds ExsD to prevent ExsD binding, thereby permitting expression of T3SS1 genes. ExsE acts as an anti-antiactivator that interacts with ExsC. H-NS is a transcriptional repressor of *exsA* through direct binding to the *exsA* promoter, whereas HlyU acts as a derepressor by displacing H-NS from the *exsA* promoter (53, 54). However, no differences in expression levels of the genes that regulate *exsA* were detected between the wrinkly and smooth phenotypes (data not shown), suggesting that these genes may not be required for the regulation of ExsA expression during phenotype switching. In addition, a total of 11 genes, including *vtrB* within Vp-PAI were significantly induced in wrinkly colonies compared with smooth colonies (Table S3). VtrB and VtrA are two novel ToxR-like transcriptional regulators encoded by genes in the Vp-PAI locus that act as master activators for the expression of Vp-PAI genes and play critical roles in the pathogenicity of V. parahaemolyticus (55). The roles of VtrB and VtrA in biofilm formation and wrinkly-smooth phenotype switching should be investigated in the future.

The expression of the majority of T6SS1 (VP1386–1420) and T6SS2 (VPA1024–1046) genes was significantly reduced in wrinkly colonies compared with smooth colonies (Table S3). T6SS1 is induced by high salt under warm-temperature growth conditions, whereas T6SS2 is active under low-salt conditions (11). T6SS1 possesses antibacterial activity and is thought to enhance the environmental competitiveness of V. parahaemolyticus (11). In other species of bacteria, the T6SSs were also shown to be required for metal ion uptake, interbacterial interactions, combating diverse stresses, host immunity, and biofilm formation (56–59). Extended studies of V. parahaemolyticus T6SS will not only help us understand the pathogenic and environmental adaptation mechanisms of the bacterium but also may illustrate the roles of T6SS in colony morphology transformation.

Extracellular proteases, including metalloproteases and serine proteases, are thought to be involved in the pathogenicity of V. parahaemolyticus (3). Extracellular proteases are enzymes that directly or indirectly digest many kinds of host proteins into amino acids, aiding the spread of pathogens into host tissues and promoting the occurrence and development of wound infections caused by V. parahaemolyticus (3). The RNA-seq data showed that one metalloprotease gene, *vppC* (VP1340), and three serine protease genes, *prtA* (VPA0227), VPA0449, and VPA1071, were significantly differentially expressed in wrinkly colonies compared with smooth colonies; of these, VPA1071 was downregulated and the other three were upregulated (Table S3). VppC, a zinc metalloprotease, is secreted by V. parahaemolyticus during the early stationary phase at 26°C and possesses strong activity against native collagen (60, 61). PrtA is highly secreted during the late-log growth of V. parahaemolyticus and possesses hemolytic and cytotoxic activities (62). In-depth studies focusing on the biological activities of extracellular proteases should be performed to discover more about their roles in the pathogenic processes of V. parahaemolyticus.

### Putative regulators that may be involved in colony morphology switching.

RNA-seq data also revealed at least 44 significantly altered genes encoding putative regulators in V. parahaemolyticus 2210633 cells during colony morphology switching, of which 33 were upregulated and 11 were downregulated in the wrinkly phenotype (Table S3). Many of these genes encode global regulators, including LysR-type transcriptional regulators, LuxR family transcriptional regulators, AraC-type regulatory proteins, TetR family transcriptional regulators, the PadR-family of regulators, and two-component response regulators, as well as other genes encoding regulators with specific functions, such as the cold shock transcriptional regulator CspA. Of these, the genes encoding the two-component system response regulator RssB (VP2183), which represses motility and colonization in V. cholerae (63); LysR family transcriptional regulators (VP2427 and VPA1687); a LuxR family transcriptional regulator (VPA0358); an AraC transcriptional regulator QsvR (VPA0606), which activates the T3SS1, T6SS2, and Vp-PAI genes but represses biofilm formation by V. parahaemolyticus (15, 64, 65); and a response regulator (VPA1130) showed expression levels in wrinkly colonies more than 20 times higher than those in smooth colonies. In contrast, genes encoding a TetR family transcriptional regulator (VP0040), which might be involved in regulating numerous aspects of bacterial pathways such as metabolism, antibiotic resistance, and quorum sensing (66), and a putative transcriptional regulator (VP0399) showed expression levels in wrinkly colonies more than three times lower than those in smooth colonies. Three regulatory genes, *aphA* (VP2762), *cpsQ* (VPA1446), and *cpsS* (VPA1447), that have been well studied in V. parahaemolyticus were also all significantly induced in wrinkly colonies. AphA, the master quorum-sensing regulator at low cell densities, is a global regulator that regulates various behaviors of V. parahaemolyticus, including motility, virulence factor production, and biofilm formation (20, 29, 64, 67, 68). CpsQ and CpsS, together with CpsR, constitute a regulatory circuit that tightly controls the expression of EPS-associated genes (69, 70). CpsS represses the transcription of *cpsA* and *cpsR*, CpsR activates *cpsQ* transcription, and CpsQ represses *cpsS* transcription (69). The simultaneous activation of CpsS and CpsQ in the wrinkly phenotype may well balance the expression levels of EPS genes. In addition, the transcriptional regulator VPA0593 is induced in the wrinkly phenotype, under low-salt and acidic conditions (71, 72), suggesting that this regulator may function as a global regulator in V. parahaemolyticus. Moreover, many other putative regulators, such as VP1737 and VP1993, that may control multiple bacterial behaviors were also significantly differentially expressed between the two phenotypes.

Small noncoding RNAs (sRNAs) are posttranscriptional regulators in bacteria that play important regulatory roles in gene expression (73). In V. parahaemolyticus, only a few sRNAs have been experimentally studied. For example, the Qrr sRNAs are *trans*-acting sRNAs that affect quorum sensing, CPS production, motility, and metabolism (20, 21, 74). The sRNA Spot42 binds to the ribosomal binding site and initiation codon of VP1682 mRNA, encoding a chaperone protein of T3SS1 to posttranscriptionally repress the translation of this mRNA (75). RNA-seq provides a way to discover novel sRNAs (76). Herein, a total of 636 putative sRNAs were detected during colony morphology switching, of which 624 were *cis*-encoded and 12 were *trans*-encoded (see Table S4 in the supplemental material). However, whether these sRNAs truly exist needs to be experimentally confirmed in the future.

In brief, these results indicated that wrinkly-smooth colony switching was strictly regulated by a complex molecular regulatory network in V. parahaemolyticus RIMD 2210633. However, the roles of most of these regulatory factors are completely unknown. The functions of these regulatory factors should be elucidated in future studies, which will help us to better understand the regulatory mechanisms involved in colony morphology switching.

### Other selected significantly expressed genes.

The *mfp* gene locus (VPA1446–1443) consists of *cpsQ*-*mfpABC* and *mfpABC* (77). CpsQ is a regulator that activates *mfpABC* transcription [3]. MfpA is a potential secreted calcium-binding protein, MfpB is a potential ABC-type transporter, and MfpC is a type I secretion membrane fusion protein (70). The *mfp* gene mutants show severe defects in biofilm formation and display altered colony morphology on Congo red agar (15). Our data showed that *cpsQ* and *mfpABC* were significantly induced and repressed in the wrinkly phenotype, respectively (Table S3). Considering that QsvR and AphA activate and repress the transcription of *cpsQ-mfpABC* and *mfpABC*, respectively (77, 78), and both were induced in the wrinkly phenotype (Table S3), genes encoding Mfp proteins should be strictly regulated and balance changes in colony morphology.

Three antioxidative genes, *katG1* (VPA0768), *katG2* (VPA0453), and *ahpC1* (VPA1683), were significantly induced in wrinkly colonies compared with smooth colonies (Table S3). AhpC is able to rapidly degrade H_2_O_2_ at low concentrations, whereas KatG acts as a scavenger at high doses of H_2_O_2_ (79). The surface morphology of wrinkled colony increases the amount of surface area in contact with air, which may increase exposure to reactive oxygen species (26). High expression of these antioxidative genes would be beneficial for V. parahaemolyticus to resist the reactive oxygen species generated in the wrinkly phenotype colony during colony morphology switching.

A total of nine genes encoding outer membrane proteins were also significantly differentially expressed in the wrinkly phenotype compared with the smooth phenotype, of which VP0636, VP1218, *ompA2* (VPA1186), and VPA1579 were upregulated, while *ompA1* (VP0764), VP1008, *ompW* (VPA0096), VPA0248, and VPA0527 were downregulated (Table S3). These results indicated that the major outer membrane proteins were remodeled during colony morphology changes. However, whether these proteins can form porin channels in the outer membrane and how they influence wrinkly-smooth colony switching need to be further investigated.

### Conclusions and outlook.

Here, we showed that V. parahaemolyticus was able to naturally and reversibly switch between wrinkly and smooth phenotypes. The RNA-seq data showed that more than 1,000 genes were significantly differentially expressed between the two phenotypes, including the major virulence gene loci and key biofilm-related genes. The majority of the genes involved in T3SS1, T6SS1, T6SS2, ChiRP production, and flagellar synthesis were downregulated in the wrinkly spreader phenotype, whereas those involved in Vp-PAI, Syp exopolysaccharide synthesis, and some antioxidative genes were upregulated. In addition, the data also showed that the wrinkly spreader grew faster in nutrient-rich conditions, had greater motility and biofilm capacities, and produced more c-di-GMP that the smooth spreader. Several genes that may be associated with c-di-GMP metabolism were also shown to be significantly differentially expressed, which may account for the different c-di-GMP levels between the two phenotypes. Most importantly, many putative regulators were significantly differentially expressed, and hundreds of sRNAs were detected during colony morphology switching, indicating that the phenotype switching is strictly regulated by a complex molecular regulatory network in V. parahaemolyticus RIMD 2210633. Taken together, the presented work highlighted the gene expression profiles related to wrinkly-smooth switching, showing that significantly differentially expressed genes were involved in various biological behaviors, including virulence factor production, biofilm formation, metabolism, adaptation, and colonization. However, transcriptome analysis is only a preliminary investigation of the mechanisms involved in colony morphology changes, and more studies should be conducted to uncover the molecular mechanisms involved in the transition between wrinkly and smooth phenotypes.

## MATERIALS AND METHODS

### Bacterial growth conditions.

V. parahaemolyticus RIMD 2210633, a pandemic O3:K6 strain isolated from a diarrhea patient in 1996 (36), was used throughout the current study. A monoclonal strain of V. parahaemolyticus was inoculated into 5 mL 2.5% (wt/vol) Bacto heart infusion (HI) (BD Bioscience, USA) broth and incubated with shaking at 200 rpm at 37°C for 12 h. The cell culture was diluted 50-fold into 5 mL 3.74% (wt/vol) Difco marine broth 2216 (M broth; BD Biosciences, USA) and incubated statically at 30°C for 48 h. The resultant cell culture was 10-fold serially diluted into phosphate-buffered saline (PBS) buffer (pH 7.2), and 200 μL of the diluted cells was spread onto an HI plate supplemented with 1.5% (wt/vol) NaCl. The plate was incubated statically at 30°C for 48 h, at which time, the colony morphology varied, demonstrating both wrinkly and smooth colonies.

### RNA extraction and RNA sequencing.

Three wrinkly and three smooth colonies were randomly collected from the HI plate using toothpicks and placed into TRIzol reagent (Invitrogen, USA) for RNA extraction. RNA concentrations were measured using a NanoDrop 2000, and RNA integrity was evaluated using agarose gel electrophoresis (80). The amount of total RNA in each sample was required to be greater than 2 μg with an OD_260_/OD_280_ between 1.8 and 2.2. The rRNA removal and mRNA enrichment were performed using an Illumina/Ribo-Zero rRNA removal kit (bacteria) (Illumina, USA) according to the manufacturer’s instructions. cDNA library construction and sequencing were performed on an Illumina HiSeq platform at Genewiz Biotechnology Co. Ltd. (Suzhou, China). Bioinformatic analysis was performed as previously described (26). Only genes with at least a 2-fold change in the ratio of mRNA levels (test/reference) and a *P* value of <0.05 were considered as significantly differently expressed genes.

### Crystal violet staining.

Crystal violet (CV) staining was performed as previously described (81). Briefly, three smooth and three wrinkly colonies were randomly picked from the HI plate and resuspended in PBS buffer (pH 7.2). The bacterial cell densities were then adjusted to an OD_600_ value of 1.4, defined here as bacterial seeds. The bacterial suspensions were 50-fold diluted into 2 mL of M broth in a 24-well cell culture plate and allowed to grow at 30°C with shaking at 150 rpm. The planktonic cells were collected for measurement of OD_600_ values. The surface-attached cells were stained with 0.1% CV. The bound CV was dissolved with 20% ethanol, and the OD_570_ values were measured as an index of CV staining. Relative biofilm formation was calculated using the following formula: OD_570_/OD_600_.

### Quantification of c-di-GMP.

Quantification of c-di-GMP levels was performed as previously described (82). Briefly, three smooth and three wrinkly colonies were randomly selected from the HI plate, resuspended in 2 mL ice-cold PBS, incubated at 100°C for 5 min, and sonicated for 15 min (power, 100%; frequency, 37 kHz) in an ice-water bath. The supernatant containing extracted c-di-GMP was collected, and the pellet was resuspended in 2 mL ice cold PBS and reextracted another two times. The intracellular c-di-GMP levels were determined using a c-di-GMP enzyme-linked immunosorbent assay (ELISA) kit (Mskbio, Beijing, China). The total protein concentration in the supernatant was determined using a Pierce bicinchoninic acid (BCA) protein assay kit (ThermoFisher Scientific, USA) according to the manufacturer’s instructions. Concentrations of c-di-GMP were expressed as picomole per gram of protein.

### Swimming motility.

Swimming motility assay was performed as previously described (35). Briefly, 2 μL of bacterial seeds was inoculated into semisolid swim plates (1% Oxoid tryptone, 2% NaCl [Merck, Germany], and 0.5% Difco Noble agar (BD Biosciences, USA]). The diameter of the area covered by the swimming bacteria was measured per hour after incubation at 37°C for 2 h. Three biological replicates were performed for each seed sample.

### Swarming motility.

Swarming motility assay was performed as previously described (67). Briefly, 2 μL of bacterial seeds was spotted onto a solid swarm plate (2.5% Bacto heart infusion, 1.5% NaCl [Merck], and 2.0% Difco Noble agar [BD Bioscience]) and incubated statically at 37°C. The diameter of the swarming zone was measured per 12 h. Three biological replicates were performed for each seed sample.

### Quantitative PCR.

The qPCR assay was performed as previously described (83). Briefly, total RNA was extracted from wrinkly and smooth colonies using TRIzol reagent (Invitrogen). The cDNA was generated from 1 μg total RNA using a FastKing First Strand cDNA synthesis kit (Tiangen Biotech, China) according to the manufacturer’s instructions. The relative mRNA levels of each target gene were determined using the classic 2^−ΔΔCt^ method. The 16S rRNA gene was used as the internal control. Primers used in this work are listed in Table S1 in the supplemental material. Experiments were performed three independent times with three biological replicates per experiment.

### Statistical methods.

Numerical results are expressed as mean ± standard deviation (SD). Paired Student's *t* tests were used to calculate statistical significance with a *P *of <0.05 considered significant.

### Data availability.

The original data presented in the study are included in the article/supplementary materials. Further inquiries can be directed to the corresponding authors. The raw data of RNA-seq are deposited in the NCBI repository under accession number PRJNA867419.
