# Supplementary material for: Transcriptomic Analysis of Vibrio parahaemolyticus Underlying the Wrinkly and Smooth Phenotypes
Source: Microbiol Spectr. 2022 Sep 13;10(5):e02188-22. doi: 10.1128/spectrum.02188-22 (PMC9604176; doi:10.1128/spectrum.02188-22)
Supplement: Supplemental file 1 — Supplemental material. Download spectrum.02188-22-s0001.pdf, PDF file, 1.9 MB [file spectrum.02188-22-s0001.pdf]

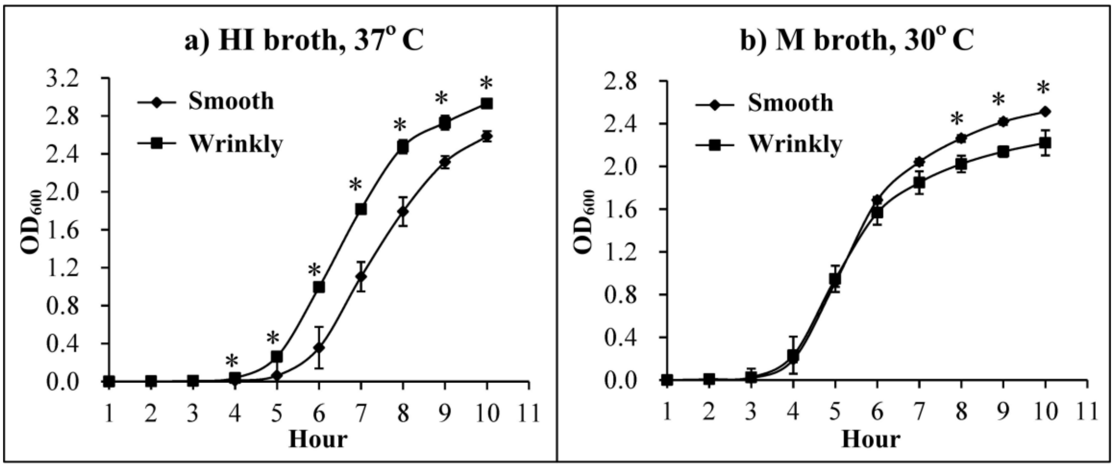

Figure S1. Growth curves of wrinkly and smooth colonies. *V. parahaemolyticus* strains were grown in M (or HI) broth at 30 ℃ (or 37 ℃) with shaking at 200 rpm, and the OD_{600} values of each culture were monitored at 1 h intervals. Experiments were performed three times with three different colonies per experiment.

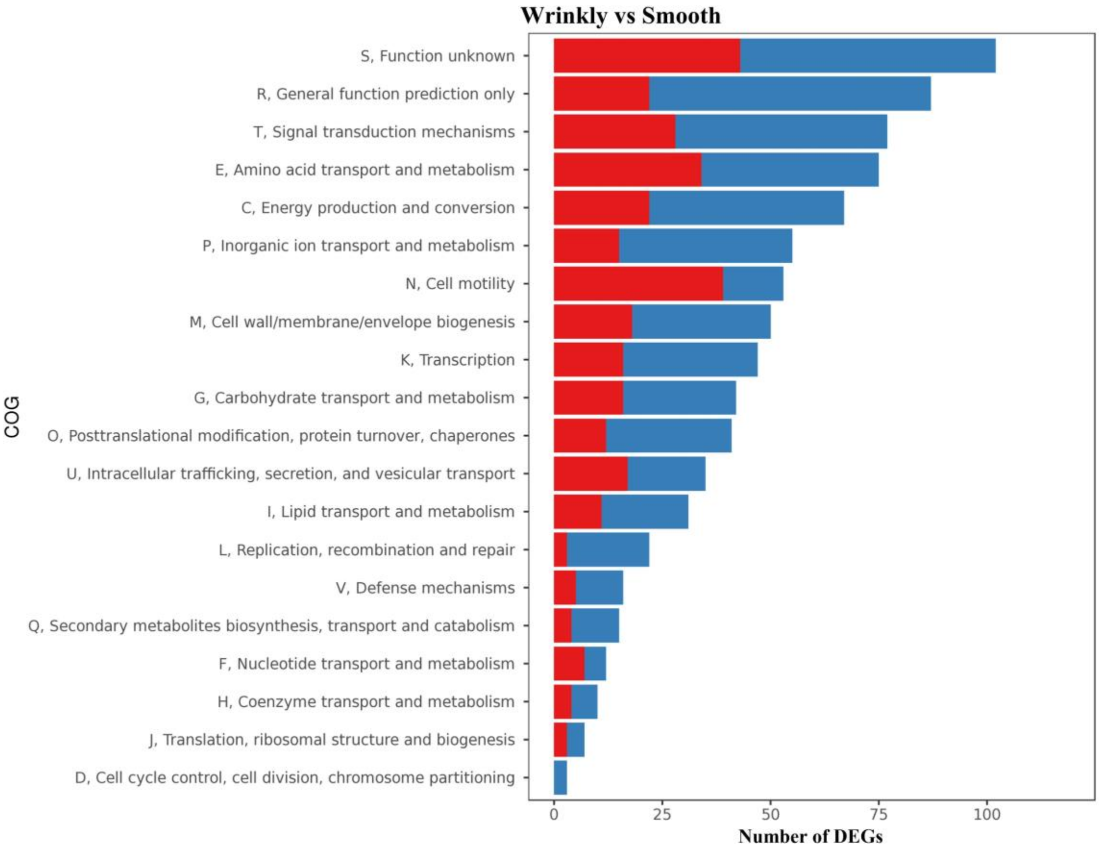

Figure S2. Differentially expressed genes categorized by Cluster of Orthologous Groups of proteins (COG). The vertical axis represents COG classification, and the horizontal axis represents the number of differentially expressed genes. Blue and red bars represent downregulated and upregulated genes, respectively.

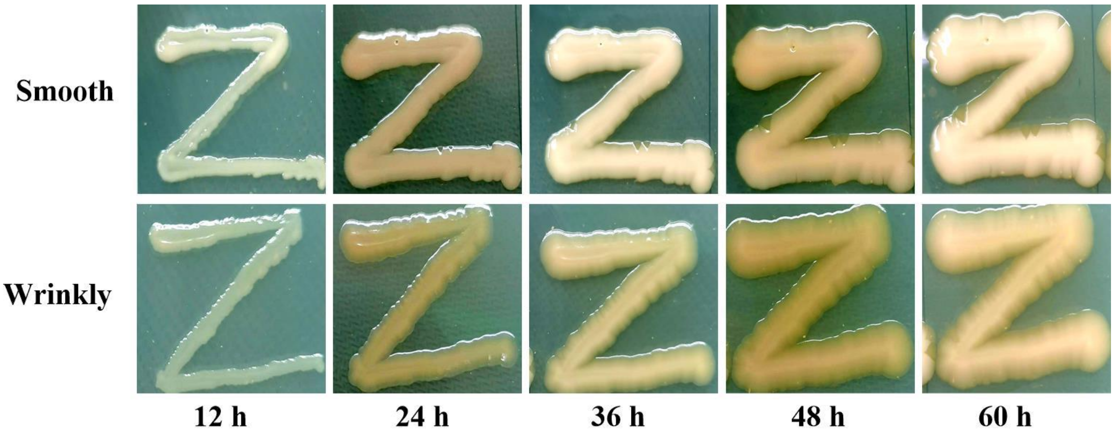

Figure S3. Detection of opaque (OP)-translucent (TR) phenotypes between the wrinkly and smooth spreaders. For detection of TR and OP variations, a small amount of each bacterial seed was taken with an inoculation loop, streaked directly on an HI plate, and statically incubated at 37 ℃.

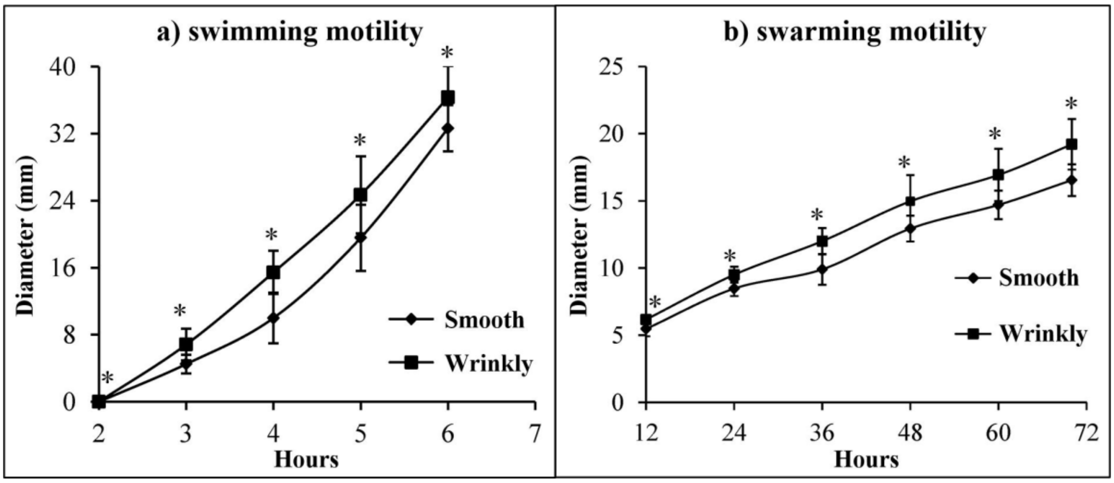

Figure S4. Motor capacity of wrinkly and smooth phenotypes. Swimming or swarming abilities of the wrinkly and smooth phenotypes were evaluated by measuring the diameter of the area covered by swimming or swarming cells in semi-solid swimming plates or swarming agars. Results were analyzed using paired Student's *t*-tests. The asterisk (*) represents $P < 0.05$.

Table S1. Primers used for qPCR in this study

Target	Primers (forward/reverse, 5'-3')
VP0040	GGGCGTTAATGACGACATGC/TTCGCTCAAGTTCCCACGTC
VP0399	AAAGAGATCGGGGTAAGCGTA/CTCTGCCACCCAACAACTCT
VP0877	CAAGCTTGTTCAACGCACCA/TAACGTCGCACTACTCACCG
VP0988	GCGCTGTGTAAAGCAAACCA/GCCAAAATCATGCCGTCGAA
VP1212	ACACTCCGGTCGTGATCCTA/ATGACCGGAGAAGCGTTACC
VP1221	CCTTGAAGGCAGACCCTCAG/CGCGTGGATAGCCTTACCTT
VP1414	GATGACCCAATGCCGTTTCG/AGCACACAAACAGTAGCGGA
VP1468	GGGCAAACTGTGGCTTTACG/TATTGAGCAGTTGCGAGGGG
VP1660	AGAAGTTCTGCTTGGCGGTT/ATGGTTGGTCGCTTTCGTCT
VP1677	GCGAACGCAGCGATTAATGA/GGCGTCGCTGTCTTTTACAC
VPA1344	CGACGCAAAACTTACCCCCA/AATTGATGGGCGATGCAGGT
VP1775	AAGAACTGGGAGCGTTACGG/ATGCCAGTTTAAGCGGGTGA
VP1782	GCTTACGGCTGGGTAACCTT/CAAAGTCCACATCGGTTGCG
VP2183	ATTTTGCCGAACAGCGTGAC/AACGCCAGTCACCTTGAGAG
VP2251	CTCGCACTGATGCGAATGTG/ATGGGCCGTCTTTACGGTTT
VP2259	ACAACGTATGCGTGACCTGT/TCAGCACCGATTTGGAACGA
VP2427	GGGACGGACTTGTTTGTTCG/CGGGTTTGGATCCTGGTTGA
VP2523	AAAGCTCTGATCACACCGGC/ACCTGCAAGGGCAGAATCAA
VP2524	CACAGCGGAAGACCCAGTAG/CATCGGGATCTTGACGCAGA
VPA0556	TGAAGCGGAATTTGTGCGTG/ATCTGGTGTTGTCGCCATGT
VPA0846	TGTGTCGCGAACAAATCGTG/GAACGCATTTCACTCGCTCG
VPA0869	CCCTAGAACACGGGCATCAG/TCCCAAGGCGCTTACGAAAT
VPA1029	CGATCATGCGATGGTGGACT/TGCTAGATCATTCCCACCGC
VPA1130	GAAAATGGCGAGCTACTGCG/GATCGCGTTCATTGGAGTGC
VPA1344	CGACGCAAAACTTACCCCCA/AATTGATGGGCGATGCAGGT
VPA1516	GTCAGTCGAGAATCCAGCGT/CGTCCAATCTTGCCGTTGAC
VPA1687	AGAGTTTTGTCGCCGCTACC/CCTGAACGACACCAGAACGA

Table S2. Significantly and differentially expressed genes in smooth colonies relative to wrinkled colonies

Gene ID	Gene Name	Fold Change	Regulation	Product
gene1679	VP1630	0.0238497	Down	calcium-binding outer membrane-like protein
gene3911	VPA0680	0.0046875	Down	arylsulfatase
gene3631	VPA0401	0.0181602	Down	hypothetical protein
gene2476	VP2421	0.0329313	Down	hypothetical protein
gene4752	VPA1514	0.0401814	Down	superoxide dismutase, Cu-Zn
gene4817	VPA1579	0.0226405	Down	outer membrane protein
gene1034	VP0988	0.0066722	Down	hypothetical protein
gene1518	VP1469	0.0334727	Down	LuxO repressor protein
gene2238	VP2183	0.0417231	Down	response regulator
gene4769	VPA1531	0.0377358	Down	serine protease
gene1033	VP0986	0.0080592	Down	hypothetical protein
gene1804	VP1755	0.0235099	Down	histidine kinase
gene1228	VP1181	0.0150588	Down	lactonizing lipase
gene4441	VPA1203	0.059365	Down	hypothetical protein
gene1523	VP1474	0.0504288	Down	capsule transport protein OtnA
gene2467	VP2412	0.061704	Down	pilus assembly protein
gene4439	VPA1201	0.060763	Down	periplasmic nitrate reductase, cytochrome c-type protein
gene1032	VP0985	0.0108398	Down	SpoVR family protein
gene4467	VPA1229	0.0295877	Down	hypothetical protein
gene4436	VPA1198	0.0227361	Down	NapD protein
gene4440	VPA1202	0.0845837	Down	polyhydroxyalkanoic acid synthase
gene4437	VPA1199	0.046354	Down	nitrate reductase catalytic subunit

gene4443	VPA1205	0.0715276	Down	acetoacetyl-CoA reductase
gene2475	VP2420	0.0287855	Down	pilus assembly protein
gene2005	VP1952	20.065222	Ups	hypothetical protein
gene3916	VPA0685	0.03524	Down	MoxR-like protein
gene3265	VPA0044	0.0129495	Down	hypothetical protein
gene3856	VPA0625	0.0904494	Down	3-hydroxyisobutyrate dehydrogenase
gene2032	VP1979	0.0449138	Down	hypothetical protein
gene2464	VP2409	0.0571253	Down	hypothetical protein
gene3693	VPA0463	0.03339	Down	hypothetical protein
gene3996	VPA0766	0.0135418	Down	hypothetical protein
gene1411	VP1361	0.0049	Down	ABC transport system permease
gene3851	VPA0620	0.091725	Down	acyl-CoA thiolase
gene4064	glgC	0.0503397	Down	glucose-1-phosphate adenylyltransferase
gene1524	VP1475	0.0521519	Down	hypothetical protein
gene4200	VPA0969	0.0769487	Down	hypothetical protein
gene3784	VPA0554	0.0343634	Down	methyl-accepting chemotaxis protein
gene3227	VPA0006	0.0359951	Down	GTP cyclohydrolase II
gene3706	VPA0476	0.0983834	Down	hypothetical protein
gene4832	VPA1594	0.0231088	Down	D-alanyl-D-alanine carboxypeptidase
gene3928	VPA0697	0.0081699	Down	nonspecific acid phosphatase
gene1087	VP1041	0.1316743	Down	gonadoliberin III-like protein
gene1458	VP1409	5.2301237	Ups	hypothetical protein
gene3429	VPA0199	0.0914556	Down	hemolysin secretion protein HylB
gene3823	VPA0593	0.0019146	Down	transcriptional regulator
gene1005	VP0958	0.0234803	Down	inosine monophosphate dehydrogenase-like protein
gene4683	VPA1445	8.7747112	Ups	secreted calcium-binding protein

gene3327	VPA0106	0.0307313	Down	hypothetical protein
gene4682	VPA1444	10.607844	Ups	transport protein
gene2466	VP2411	0.0510155	Down	tight adherence TadB-like transmembrane protein
gene849	VP0809	0.1880223	Down	sugar nucleotide epimerase
gene4742	VPA1504	0.0264086	Down	CsuD protein
gene3874	VPA0643	0.0190801	Down	electron transfer flavoprotein-ubiquinone oxidoreductase
gene4816	VPA1578	0.0165186	Down	hypothetical protein
gene1460	VP1411	9.2120593	Ups	hypothetical protein
gene4442	VPA1204	0.077724	Down	acetyl-CoA acetyltransferase
gene4339	VPA1101	0.0244754	Down	cytochrome c551 peroxidase
gene3822	VPA0592	0.0492076	Down	hypothetical protein
gene1457	VP1408	5.2000301	Ups	IcmF-like protein
gene370	VP0360	0.1139634	Down	hypothetical protein
gene4438	VPA1200	0.0569625	Down	periplasmic nitrate reductase, cytochrome c-type protein
gene2474	VP2419	0.042692	Down	pilus assembly transmembrane protein
gene4821	VPA1583	0.046286	Down	hypothetical protein
gene2673	VP2607	0.0206079	Down	cytochrome c oxidase subunit I
gene4435	VPA1197	0.0127362	Down	(Fe-S)-binding protein
gene4311	VPA1074	0.1699051	Down	hypothetical protein
gene3852	VPA0621	0.0934261	Down	methylmalonate-semialdehyde dehydrogenase
gene1926	VP1877	0.106799	Down	guanylate cyclase-like protein
gene4344	VPA1106	0.025598	Down	stomatin-like protein
gene4743	VPA1505	0.0095305	Down	CsuC protein
gene2030	VP1977	0.0498818	Down	hypothetical protein

gene4745	VPA1507	0.0098368	Down	CsuA protein
gene1267	VP1220	0.0143235	Down	83 kDa decaheme outer membrane cytochrome c
gene2465	VP2410	0.0526718	Down	tight adherence TadC-like transmembrane protein
gene3229	VPA0008	0.0372546	Down	amino acid ABC transporter periplasmic amino acid-binding portion
gene4424	VPA1186	0.2112106	Down	outer membrane protein OmpA
gene3815	VPA0585	4.2709253	Ups	tryptophan synthase subunit beta
gene1220	VP1173	0.1382918	Down	phage shock protein A
gene3677	VPA0447	0.0797313	Down	hypothetical protein
gene1463	VP1414	12.123949	Ups	hypothetical protein
gene3850	VPA0619	0.0296829	Down	transcriptional regulator
gene583	VP0565	0.0765129	Down	hypothetical protein
gene3107	VP2979	3.0436093	Ups	GGDEF family protein
gene4055	VPA0824	22.916671	Ups	regulatory protein UhpC
gene2479	VP2424	0.0099638	Down	hypothetical protein
gene4201	VPA0970	0.1134729	Down	hypothetical protein
gene4286	VPA1049	0.0310051	Down	two-component response regulator
gene2477	VP2422	0.0040718	Down	hypothetical protein
gene1462	VP1413	10.891918	Ups	hypothetical protein
gene4453	VPA1215	0.096742	Down	hypothetical protein
gene2174	VP2120	0.2721545	Down	short chain dehydrogenase
gene3600	VPA0371	0.2596401	Down	hypothetical protein
gene1522	VP1473	0.0820345	Down	capsular polysaccharide biosynthesis
gene3847	VPA0616	0.0342293	Down	enoyl-CoA hydratase/isomerase
gene1648	VP1599	0.2401048	Down	hypothetical protein
gene3853	VPA0622	0.093253	Down	acyl-CoA dehydrogenase

gene3917	VPA0686	0.0436972	Down	hypothetical protein
gene4369	VPA1131	0.0106002	Down	periplasmic binding protein-like protein
gene2727	VP2640	0.0302095	Down	hypothetical protein
gene3037	VP2920	2.6148574	Ups	anti-RNA polymerase sigma 70 factor
gene1268	VP1221	0.0089106	Down	cytochrome subunit of sulfide dehydrogenase
gene2468	VP2413	0.0399363	Down	pilus assembly protein
gene3857	fabG	0.1304828	Down	3-ketoacyl-ACP reductase
gene4100	VPA0869	0.1875232	Down	GGDEF family protein
gene1387	VP1340	0.0298196	Down	collagenase
gene1086	VP1040	0.1059675	Down	hypothetical protein
gene1823	VP1774	8.1745623	Ups	carbon-nitrogen hydrolase
gene4681	VPA1443	6.6681548	Ups	protein secretion protein
gene4819	VPA1581	0.0239411	Down	hypothetical protein
gene3292	VPA0071	0.2387446	Down	alcohol dehydrogenase
gene1805	VP1756	0.0331712	Down	hypothetical protein
gene1831	VP1782	12.932697	Ups	hypothetical protein
gene312	VP0302	0.0795906	Down	hypothetical protein
gene3678	VPA0448	0.094695	Down	hypothetical protein
gene1459	VP1410	10.611353	Ups	hypothetical protein
gene3508	VPA0278	0.1349632	Down	isopentenyl pyrophosphate isomerase
gene1474	VP1425	0.2386786	Down	alcohol dehydrogenase
gene3324	VPA0103	0.1307488	Down	hypothetical protein
gene4368	VPA1130	0.0365621	Down	sensory box sensor histidine kinase/response regulator
gene4049	VPA0818	4.1850362	Ups	hypothetical protein
gene3430	VPA0200	0.2878494	Down	hypothetical protein
gene3849	VPA0618	0.0252512	Down	acyl-CoA dehydrogenase

gene2389	VP2334	0.3563523	Down	hypothetical protein
gene1517	VP1468	0.0596012	Down	hexosyltransferase
gene2741	pyrB	3.7832682	Ups	aspartate carbamoyltransferase
gene1036	VP0989	0.0047485	Down	hypothetical protein
gene2493	VP2439	0.1516078	Down	hydrolase
gene3198	VP3057	13.114624	Ups	ComM-like protein
gene4398	VPA1160	0.1295278	Down	hypothetical protein
gene3683	VPA0453	0.1122581	Down	catalase/peroxidase
gene3966	VPA0735	0.1233883	Down	hypothetical protein
gene4034	VPA0803	2.8881755	Ups	serine hydroxymethyltransferase
gene4287	VPA1050	0.0140877	Down	hypothetical protein
gene4338	VPA1100	0.0418241	Down	sensor histidine kinase
gene1266	VP1219	0.0223642	Down	deca-heme c-type cytochrome
gene3027	VP2910	0.1614083	Down	hypothetical protein
gene4009	VPA0778	0.0505742	Down	Mg transporter MgtE
gene1450	VP1401	8.7043555	Ups	hypothetical protein
gene4762	VPA1524	2.9222653	Ups	hypothetical protein
gene3291	VPA0070	0.2074486	Down	esterase
gene1576	potC	0.2300985	Down	spermidine/putrescine ABC transporter membrane protein
gene1222	VP1175	0.2154862	Down	phage shock protein C
gene1449	VP1400	6.7501579	Ups	hypothetical protein
gene4685	VPA1447	6.4973285	Ups	LuxR family transcriptional regulator
gene4155	VPA0924	0.2315256	Down	hypothetical protein
gene3999	VPA0768	0.1902298	Down	catalase/peroxidase
gene2472	VP2417	0.0536196	Down	hypothetical protein

gene1260	VP1213	0.3665768	Down	hypothetical protein
gene1822	VP1773	9.0767989	Ups	hypothetical protein
gene1308	VP1261	0.1722496	Down	hypothetical protein
gene1437	VP1388	3.6866489	Ups	hypothetical protein
gene4343	VPA1105	0.0447309	Down	hypothetical protein
gene1654	VP1605	0.0414804	Down	hypothetical protein
gene1691	VP1642	0.0721443	Down	trypsin
gene3627	VPA0397	0.0329032	Down	hypothetical protein
gene3848	VPA0617	0.0448568	Down	propionyl-CoA carboxylase subunit beta
gene2421	VP2366	0.0538492	Down	GGDEF family protein
gene3826	VPA0596	0.0587254	Down	methyl-accepting chemotaxis protein
gene1830	VP1781	12.581212	Ups	glutamine synthetase
gene4054	VPA0823	11.045916	Ups	pyruvate kinase
gene3478	VPA0248	6.7909166	Ups	outer membrane protein OmpA
gene1453	VP1404	7.7916371	Ups	hypothetical protein
gene3020	VP2903	0.2448717	Down	hypothetical protein
gene1412	VP1363	0.0155908	Down	ABC transporter ATP-binding protein
gene1698	VP1649	0.1654651	Down	GntR family transcriptional regulator
gene3794	VPA0564	0.1318339	Down	hypothetical protein
gene1465	VP1416	3.6451609	Ups	hypothetical protein
gene1088	VP1043	0.110717	Down	hypothetical protein
gene1454	VP1405	6.6251954	Ups	hypothetical protein
gene1413	VP1364	0.0675633	Down	ABC transporter periplasmic substrate-binding protein
gene1821	VP1772	6.6074706	Ups	succinate-semialdehyde dehydrogenase
gene1078	torD	0.3311809	Down	chaperone protein TorD
gene2463	VP2408	0.0218975	Down	hypothetical protein

gene3412	VPA0183	2.6571515	Ups	C4-dicarboxylate transport transcriptional regulatory protein
gene3266	VPA0045	0.2966925	Down	ada regulatory protein
gene452	VP0441	2.2065502	Ups	ubiquinol-cytochrome c reductase, iron-sulfur subunit
gene1828	VP1779	16.930844	Ups	glutamine amidotransferase
gene3854	VPA0623	0.094945	Down	enoyl-CoA hydratase
gene3411	VPA0182	3.5649481	Ups	C4-dicarboxylate transport sensor protein
gene1373	VP1326	3.5820685	Ups	hypothetical protein
gene3736	VPA0506	0.0902025	Down	hypothetical protein
gene641	VP0623	3.4907268	Ups	D-amino acid dehydrogenase small subunit
gene602	VP0584	0.2821263	Down	isocitrate lyase
gene1452	VP1403	8.8599404	Ups	hypothetical protein
gene640	VP0622	2.148279	Ups	sodium/alanine symporter
gene3920	VPA0689	0.0538617	Down	hypothetical protein
gene1965	VP1916	0.2516129	Down	amidase
gene4381	VPA1143	0.0269699	Down	molybdenum containing oxidoreductase
gene2397	VP2342	0.2110534	Down	DNA polymerase IV
gene4741	VPA1503	0.0509264	Down	CsuE protein
gene1925	VP1876	0.1690435	Down	hypothetical protein
gene3855	VPA0624	0.1260396	Down	enoyl-CoA hydratase/isomerase
gene3878	VPA0647	0.0794193	Down	pyruvate dehydrogenase E1 component, alpha subunit
gene1414	VP1365	0.190887	Down	hypothetical protein
gene3971	VPA0740	0.2403192	Down	transcriptional regulator
gene1726	VP1677	0.1532788	Down	hypothetical protein
gene1699	VP1650	0.4273528	Down	hypothetical protein
gene4766	VPA1528	0.0346708	Down	hypothetical protein

gene1979	VP1930	0.1882559	Down	hypothetical protein
gene2742	VP2655	4.405	Ups	aspartate carbamoyltransferase
gene551	VP0540	3.5150662	Ups	carbon starvation protein A
gene4268	VPA1031	5.1017323	Ups	hypothetical protein
gene1456	VP1407	5.2341871	Ups	transcriptional regulator
gene572	VP0561	0.3902252	Down	ClpB protein
gene4724	napG	0.0579378	Down	quinol dehydrogenase periplasmic subunit
gene1826	VP1777	6.0489351	Ups	aldehyde dehydrogenase
gene3796	VPA0566	0.4267138	Down	alcohol dehydrogenase
gene1239	VP1192	0.0834877	Down	outer membrane lipoprotein Pcp
gene1028	VP0981	0.2025151	Down	Rec2-like protein
gene3714	VPA0484	0.3294573	Down	hypothetical protein
gene4228	VPA0995	0.0420428	Down	hypothetical protein
gene3686	VPA0456	0.1894588	Down	transcriptional regulator
gene1520	VP1472	0.1067175	Down	phosphorelay protein
gene4492	VPA1254	0.1453612	Down	IS1328 transposase
gene373	gldA	3.0740679	Ups	glycerol dehydrogenase
gene4032	VPA0801	2.4397779	Ups	glycine dehydrogenase
gene4876	VPA1638	3.6385289	Ups	pullulanase
gene1271	VP1224	3.0313761	Ups	adhesin
gene2724	VP2638	0.3792905	Down	deacetylase DA1
gene4820	VPA1582	0.0397123	Down	hypothetical protein
gene3871	VPA0640	0.0525382	Down	protocatechuate 3,4-dioxygenase beta chain protein
gene2488	deoD	0.3955196	Down	purine nucleoside phosphorylase
gene1824	VP1775	9.9123215	Ups	oxidoreductase
gene1810	VP1761	0.1056097	Down	PRS2 protein

gene1164	VP1117	0.3916337	Down	hypothetical protein
gene1451	VP1402	8.6537497	Ups	hypothetical protein
gene1697	prpB	0.2181722	Down	2-methylisocitrate lyase
gene3337	VPA0116	3.5763557	Ups	permease
gene45	VP0040	0.3716163	Down	TetR family transcriptional regulator
gene1820	VP1771	6.2344388	Ups	4-aminobutyrate transaminase
gene4833	VPA1595	2.2264955	Ups	prolyl aminopeptidase
gene2708	VP2630	0.444483	Down	succinate-semialdehyde dehydrogenase
gene646	VP0628	0.2854143	Down	hypothetical protein
gene2261	VP2206	0.3429363	Down	peptidase insulinase family protein
gene563	VP0552	0.2393045	Down	soluble lytic murein transglycosylase
gene4865	VPA1627	2.1798862	Ups	hypothetical protein
gene4240	VPA1005	2.3703436	Ups	D-lactate dehydrogenase
gene3899	VPA0668	0.3723562	Down	hypothetical protein
gene1223	VP1176	0.3528708	Down	multidrug resistance protein
gene3575	VPA0345	0.0521321	Down	hypothetical protein
gene1503	VP1454	3.1082704	Ups	hypothetical protein
gene1466	VP1417	3.3294899	Ups	hypothetical protein
gene1793	VP1744	2.3760674	Ups	thermostable carboxypeptidase 1
gene3877	VPA0646	0.0846931	Down	pyruvate dehydrogenase E1 component subunit beta
gene311	VP0301	0.0929375	Down	ABC transporter ATP-binding protein
gene416	dnaG	0.3412471	Down	DNA primase
gene805	VP0766	0.0929094	Down	hypothetical protein
gene3554	VPA0324	0.1476666	Down	hypothetical protein
gene3986	VPA0755	0.1570161	Down	metalloproteinase
gene4507	VPA1269	0.3770443	Down	hypothetical protein

gene1676	VP1627	0.1064531	Down	acylphosphatase
gene2194	VP2139	0.2210185	Down	hypothetical protein
gene4158	VPA0927	0.2910606	Down	regulatory components of sensory transduction system
gene1227	VP1180	0.0376166	Down	lipase chaperone
gene2069	VP2016	3.8260866	Ups	hypothetical protein
gene1492	VP1443	0.232011	Down	hypothetical protein
gene3280	VPA0059	0.2886874	Down	GGDEF family protein
gene4937	VPA1699	0.2875504	Down	DEAD-box ATP dependent DNA helicase
gene4036	VPA0805	2.1363154	Ups	glycine cleavage system protein T2
gene3921	VPA0690	0.1312372	Down	hypothetical protein
gene2223	VP2168	0.4136846	Down	hypothetical protein
gene4266	VPA1029	5.5897893	Ups	hypothetical protein
gene1225	VP1178	0.4304758	Down	AcrB/AcrD/AcrF family transporter
gene4157	VPA0926	0.3896439	Down	hypothetical protein
gene601	VP0583	0.2472608	Down	malate synthase
gene1441	VP1392	6.8268832	Ups	ClpA/B-type protease
gene3684	VPA0454	0.0288896	Down	DNA-binding stress protein
gene3193	VP3052	2.440162	Ups	FixG-like protein
gene3206	VP3065	0.2911766	Down	cyclohexadienyl dehydratase signal peptide protein
gene2769	VP2682	0.3139438	Down	ATP-dependent helicase HepA
gene1440	VP1391	5.272981	Ups	transcriptional regulator
gene4530	VPA1292	0.2256526	Down	adenosine deaminase
gene94	VP0084	0.1135252	Down	hypothetical protein
gene1265	VP1218	0.0238301	Down	outer membrane protein
gene2469	VP2414	0.051372	Down	hypothetical protein
gene1483	VP1434	6.3461528	Ups	V10 pilin

gene1461	VP1412	6.9626509	Ups	hypothetical protein
gene1649	VP1601	2.4706944	Ups	dihydroorotate dehydrogenase 2
gene1480	VP1431	2.1929367	Ups	ATP-binding protein of a transport system
gene2770	VP2683	0.1633212	Down	hypothetical protein
gene3804	VPA0574	2.5057272	Ups	methyltransferase
gene2443	glpD	0.2053236	Down	glycerol-3-phosphate dehydrogenase
gene1224	VP1177	0.3460899	Down	periplasmic linker protein
gene1638	VP1589	0.419515	Down	hypothetical protein
gene4232	VPA0999	3.7497371	Ups	cytoplasmic alpha-amylase
gene4008	VPA0777	0.2011707	Down	hypothetical protein
gene1563	VP1514	5.5745439	Ups	formate dehydrogenase, iron-sulfur subunit
gene1401	VP1354	0.047634	Down	hypothetical protein
gene1269	VP1222	0.1240087	Down	cytochrome c-type protein
gene3766	VPA0536	0.0686466	Down	cytochrome c oxidase subunit II
gene3786	VPA0556	3.5152128	Ups	GGDEF family protein
gene3025	VP2908	0.2228355	Down	hypothetical protein
gene2431	VP2376	0.1179967	Down	hypothetical protein
gene3876	VPA0645	0.1005814	Down	branched-chain alpha-keto acid dehydrogenase subunit E2
gene4052	VPA0821	0.326359	Down	amino acid ABC transporter permease
gene1455	VP1406	2.8575103	Ups	hypothetical protein
gene2057	VP2004	0.0892483	Down	hypothetical protein
gene4723	napH	0.0685045	Down	quinol dehydrogenase membrane subunit
gene2962	cpxP	0.3131411	Down	periplasmic repressor CpxP
gene3757	VPA0527	7.5003512	Ups	outer membrane protein N
gene1099	VP1053	0.3888098	Down	cytochrome d ubiquinol oxidase subunit I

gene122	VP0112	0.425316	Down	methyltransferase-like protein
gene4725	VPA1487	3.0891165	Ups	acetyltransferase
gene1939	VP1890	0.2846025	Down	virulence-associated protein VacB/RNase R
gene4744	VPA1506	0.015931	Down	CsuB protein
gene453	VP0442	2.3199048	Ups	ubiquinol-cytochrome c reductase, cytochrome b
gene207	VP0197	0.4826701	Down	capsular polysaccharide biosynthesis protein D
gene4245	yieM	3.0799624	Ups	hypothetical protein
gene3477	VPA0247	5.8369119	Ups	hypothetical protein
gene4657	VPA1419	2.5576589	Ups	3-hydroxyisobutyrate dehydrogenase
gene4873	VPA1635	5.4913947	Ups	ornithine decarboxylase
gene4736	VPA1498	2.5915274	Ups	L-lactate permease
gene2034	VP1981	0.2562968	Down	methyl-accepting chemotaxis protein
gene1100	VP1054	0.3921333	Down	cytochrome d ubiquinol oxidase subunit II
gene3421	tnaA	0.451269	Down	tryptophanase
gene87	VP0077	0.4028908	Down	ferritin
gene154	VP0144	2.0831986	Ups	3'(2'),5'-bisphosphate nucleotidase CysQ
gene1669	VP1620	0.1051302	Down	amino acid ABC transporter substrate-binding protein
gene310	VP0300	0.2341861	Down	hypothetical protein
gene3257	VPA0036	2.2455171	Ups	hypothetical protein
gene3919	VPA0688	0.0779549	Down	hypothetical protein
gene95	VP0085	0.1116302	Down	hypothetical protein
gene454	VP0443	2.225109	Ups	ubiquinol-cytochrome c reductase, cytochrome c1
gene537	VP0526	5.4640128	Ups	NptA protein
gene3402	VPA0173	0.3749824	Down	ribosomal protein S6 modification protein
gene2860	VP2768	0.1487336	Down	bacterioferritin
gene1366	VP1319	5.0872735	Ups	hypothetical protein

gene1727	VP1678	0.2415901	Down	dienelactone hydrolase
gene4227	VPA0994	0.0312815	Down	hypothetical protein
gene3745	VPA0515	0.1612995	Down	hypothetical protein
gene2027	VP1974	4.7268263	Ups	5-methyltetrahydropteroyltriglutamate--homocysteine S-methyltransferase
gene1423	VP1374	2.3696828	Ups	transporter
gene4499	VPA1261	2.7181197	Ups	ATP-binding protein
gene3997	VPA0765	0.0152953	Down	glutamate synthetase
gene2629	mazG	0.1110466	Down	nucleoside triphosphate pyrophosphohydrolase
gene2089	VP2036	0.2855269	Down	DNA polymerase III alpha chain
gene4539	VPA1301	0.4714872	Down	hypothetical protein
gene377	VP0367	2.6127616	Ups	DNA-binding transcriptional regulator DhaR
gene2619	VP2553	2.1688635	Ups	RNA polymerase sigma factor RpoS
gene3621	VPA0391	0.0427491	Down	hypothetical protein
gene528	VP0517	0.2040646	Down	hypothetical protein
gene2424	mltA	0.4501797	Down	murein transglycosylase A
gene3556	VPA0326	0.0162901	Down	hypothetical protein
gene316	VP0306	2.2698462	Ups	methionine sulfoxide reductase A
gene445	VP0434	2.2909569	Ups	cytochrome d ubiquinol oxidase subunit III
gene2719	VP2633	0.3279047	Down	hypothetical protein
gene1825	VP1776	10.668803	Ups	aldehyde dehydrogenase
gene3342	VPA0121	6.09938	Ups	hypothetical protein
gene1579	VP1530	0.2744838	Down	hypothetical protein
gene3705	VPA0475	5.3105048	Ups	hypothetical protein
gene1642	VP1593	0.4295081	Down	ribosome modulation factor
gene3981	VPA0750	2.1658412	Ups	chloramphenicol-sensitive protein RarD

gene2262	VP2207	0.0959492	Down	CG2 omega domain-containing protein
gene1290	VP1243	2.0673382	Ups	hypothetical protein
gene2437	glpT	4.3036245	Ups	sn-glycerol-3-phosphate transporter
gene3256	VPA0035	0.1447645	Down	sodium/glutamate symporter
gene4265	VPA1028	5.9667452	Ups	ClpA/B-type chaperone
gene3560	VPA0330	0.2747786	Down	hypothetical protein
gene4957	VPA1719	2.4828173	Ups	hypothetical protein
gene4388	VPA1150	2.1006172	Ups	high-affinity branched-chain amino acid transport permease
gene2817	pspG	0.0709495	Down	phage shock protein G
gene2079	VP2026	2.0419904	Ups	orotidine 5'-phosphate decarboxylase
gene1920	VP1871	0.3965389	Down	hypothetical protein
gene4404	VPA1166	0.17754	Down	chloride channel protein
gene4684	VPA1446	2.9995349	Ups	LuxR family transcriptional regulator
gene1442	VP1393	5.2208891	Ups	BfdA protein
gene3918	VPA0687	0.0889334	Down	hypothetical protein
gene410	VP0400	3.2278102	Ups	transmembrane protein
gene863	hemH	2.0156869	Ups	ferrochelatase
gene2125	nhaB	2.0097124	Ups	sodium/proton antiporter
gene3112	VP2984	2.1860401	Ups	diaminopimelate decarboxylase
gene1946	VP1897	0.0359337	Down	hypothetical protein
gene3770	VPA0541	0.0707377	Down	hypothetical protein
gene1372	VP1325	3.502353	Ups	hypothetical protein
gene4811	VPA1573	2.6970784	Ups	hypothetical protein
gene1365	VP1318	11.45855	Ups	flippase
gene2446	VP2391	0.3025883	Down	hypothetical protein

gene4271	VPA1034	5.1204768	Ups	hypothetical protein
gene504	VP0493	0.4015144	Down	hypothetical protein
gene4214	VPA0983	0.1831647	Down	hypothetical protein
gene4971	VPA1733	0.2815408	Down	HlyD family secretion protein
gene3506	VPA0276	0.4652884	Down	integral membrane protein
gene3912	VPA0681	0.0373403	Down	hypothetical protein
gene4345	VPA1107	0.2295556	Down	hypothetical protein
gene115	VP0105	0.4071976	Down	hypothetical protein
gene3309	VPA0088	0.3210069	Down	oligogalacturonate lyase
gene1049	VP1002	2.5579721	Ups	hypothetical protein
gene3867	artP	0.4039822	Down	arginine transporter ATP-binding subunit
gene4508	VPA1270	0.3490122	Down	hypothetical protein
gene4272	VPA1035	4.4273161	Ups	hypothetical protein
gene4107	VPA0876	2.0174138	Ups	hypothetical protein
gene2815	VP2723	0.0170714	Down	hypothetical protein
gene1376	VP1329	3.5498011	Ups	fatty aldehyde dehydrogenase
gene1767	VP1718	4.1624935	Ups	cytochrome c551 peroxidase
gene1557	VP1508	2.5173865	Ups	hypothetical protein
gene3980	VPA0749	2.6690767	Ups	hypothetical protein
gene4044	VPA0813	0.310411	Down	bifunctional PTS system fructose-specific transporter subunit IIA/HPr protein
gene3401	VPA0172	0.431683	Down	hypothetical protein
gene1807	VP1758	0.323748	Down	galactoside O-acetyltransferase
gene4689	VPA1450	2.4721346	Ups	MoxR-like protein
gene3567	VPA0337	0.4331614	Down	hypothetical protein
gene2717	VP2631	0.4405675	Down	HD-GYP domain-containing protein

gene3740	VPA0510	0.1654153	Down	hypothetical protein
gene4490	VPA1252	0.0918477	Down	hypothetical protein
gene2660	VP2594	0.3017841	Down	hypothetical protein
gene1696	VP1647	0.2157843	Down	methylcitrate synthase
gene4261	VPA1024	4.5125838	Ups	hypothetical protein
gene1530	VP1481	0.3317664	Down	hypothetical protein
gene2225	VP2170	0.0553871	Down	transglycosylase associated gene
gene3915	VPA0684	0.1667231	Down	arylsulfatase regulator
gene803	VP0764	5.0848499	Ups	outer membrane protein OmpA
gene4925	VPA1687	0.4768726	Down	transcriptional regulator
gene3016	VP2899	2.2542405	Ups	hypothetical protein
gene1381	VP1333	2.4503743	Ups	ornithine cyclodeaminase
gene3932	VPA0701	2.4514061	Ups	oxalate/formate antiporter
gene717	VP0699	2.354	Ups	GGDEF family protein
gene4269	VPA1032	4.4764247	Ups	hypothetical protein
gene76	VP0066	3.5389108	Ups	purine nucleoside phosphorylase
gene3622	VPA0392	0.4295467	Down	hypothetical protein
gene2533	VP2476	2.4769265	Ups	permease
gene1047	VP1000	2.0352882	Ups	amino acid ABC transporter permease
gene2126	VP2073	2.6040137	Ups	disulfide bond formation protein B
gene799	VP0760	2.5453312	Ups	chitoporin
gene4872	potE	5.151058	Ups	putrescine transporter
gene372	VP0362	0.4318826	Down	two component sensor protein
gene3252	VPA0031	0.468773	Down	sodium/glutamate symporter
gene4450	VPA1212	0.0126823	Down	hypothetical protein
gene1367	VP1320	3.6007484	Ups	CDP-ribitol pyrophosphorylase

gene3880	VPA0649	0.1032901	Down	hypothetical protein
gene1927	VP1878	0.406727	Down	MutT/nudix family protein
gene3614	VPA0384	2.5830957	Ups	hypothetical protein
gene4011	VPA0780	2.142042	Ups	hypothetical protein
gene3431	VPA0201	0.1673315	Down	malate synthase
gene4335	VPA1097	2.2657243	Ups	hypothetical protein
gene2882	VP2790	0.4709077	Down	hydrolase
gene2436	glpQ	3.5928727	Ups	glycerophosphodiester phosphodiesterase
gene1481	VP1432	2.3019067	Ups	ATP-binding protein of a transport system
gene4382	VPA1144	0.0583708	Down	hypothetical protein
gene949	VP0908	3.0074922	Ups	sugar transport system permease
gene3408	VPA0179	0.2545557	Down	hypothetical protein
gene3846	VPA0615	0.3467464	Down	hydroxymethylglutaryl-CoA lyase
gene4542	VPA1304	0.2081722	Down	3-oxoacyl-ACP reductase
gene4738	VPA1500	0.3259489	Down	hypothetical protein
gene1141	VP1094	2.466053	Ups	hypothetical protein
gene4654	VPA1416	0.4599994	Down	oxidoreductase
gene3521	VPA0291	0.1796889	Down	hypothetical protein
gene3348	VPA0127	0.4634992	Down	cytochrome c-type protein YecK
gene3326	VPA0105	0.1194833	Down	LysR family transcriptional regulator
gene2045	VP1992	2.9990937	Ups	hypothetical protein
gene1382	VP1335	3.771881	Ups	dihydrodipicolinate synthetase
gene4039	VPA0808	0.2996051	Down	periplasmic linker protein
gene4043	VPA0812	0.3162047	Down	1-phosphofructokinase
gene4397	VPA1159	2.4678825	Ups	guanosine 5'-monophosphate oxidoreductase
gene4267	VPA1030	4.7397916	Ups	hypothetical protein

gene568	VP0557	0.4200486	Down	glycosylase
gene3716	VPA0486	0.0948736	Down	hypothetical protein
gene2068	VP2015	2.7924886	Ups	cytochrome c
gene1263	VP1216	0.4928455	Down	bicyclomycin/multidrug efflux system
gene3302	VPA0081	3.4397334	Ups	hypothetical protein
gene2355	VP2300	4.9772739	Ups	cytochrome c554
gene1486	VP1437	0.0416103	Down	hypothetical protein
gene3007	VP2890	0.1781862	Down	lysine decarboxylase
gene3476	VPA0246	0.46342	Down	hypothetical protein
gene2470	VP2415	0.0792506	Down	hypothetical protein
gene3153	VP3019	0.4967985	Down	multidrug resistance protein
gene3335	VPA0114	0.0181451	Down	hypothetical protein
gene3767	VPA0537	0.2868871	Down	cytochrome c oxidase subunit I
gene2580	VP2523	6.3749946	Ups	type IV pilin PilA
gene4726	VPA1488	0.4869921	Down	hypothetical protein
gene4911	VPA1673	3.6434652	Ups	L-arabinose-binding periplasmic protein
gene4848	VPA1610	0.4106248	Down	hypothetical protein
gene1221	pspB	0.217295	Down	phage shock protein B
gene4525	VPA1287	0.368072	Down	transporter
gene4218	VPA0987	0.2080269	Down	nitrite reductase large subunit
gene2203	VP2147	0.0564967	Down	hypothetical protein
gene4480	codB	0.3064134	Down	cytosine permease
gene2519	VP2462	2.1996508	Ups	dihydropteroate synthase
gene1415	VP1366	0.2919786	Down	pyridoxine kinase
gene3840	VPA0609	0.1571179	Down	hypothetical protein
gene4864	VPA1626	2.0863664	Ups	Sco1-like protein

gene368	VP0358	2.3948319	Ups	DeoR family transcriptional regulator
gene3884	VPA0654	2.1967366	Ups	hypothetical protein
gene3290	VPA0069	0.4919904	Down	hypothetical protein
gene3317	VPA0096	2.2268564	Ups	outer membrane protein W
gene3618	VPA0388	3.3149491	Ups	regulatory protein
gene1472	VP1423	0.4269212	Down	hypothetical protein
gene3550	VPA0320	0.3018574	Down	lipoprotein
gene3420	VPA0191	0.052034	Down	hypothetical protein
gene4262	VPA1025	4.2585773	Ups	hypothetical protein
gene1101	VP1055	0.3881438	Down	hypothetical protein
gene2088	VP2035	0.2869756	Down	hypothetical protein
gene4510	VPA1272	2.6664948	Ups	hypothetical protein
gene4370	VPA1132	0.274352	Down	hypothetical protein
gene2536	VP2479	2.0439333	Ups	peptide ABC transporter periplasmic peptide-binding protein
gene2482	VP2427	0.4407233	Down	LysR family transcriptional regulator
gene4226	VPA0993	0.4319213	Down	hypothetical protein
gene1801	VP1752	2.2919545	Ups	fimbrial biogenesis and twitching motility protein
gene477	VP0466	0.4890737	Down	hypothetical protein
gene3507	VPA0277	0.4151812	Down	hypothetical protein
gene3459	ulaA	3.0641901	Ups	PTS system ascorbate-specific transporter subunit IIC
gene582	mepA	0.3623616	Down	penicillin-insensitive murein endopeptidase
gene2046	VP1993	0.2967912	Down	transcriptional regulator
gene1496	VP1447	0.187933	Down	anaerobic dimethyl sulfoxide reductase subunit A
gene644	VP0626	3.168819	Ups	hypothetical protein
gene3571	VPA0341	0.3892389	Down	hypothetical protein

gene4421	VPA1183	0.1038315	Down	hypothetical protein
gene4230	VPA0997	0.124306	Down	lipase-like protein
gene4263	VPA1026	3.5566835	Ups	hypothetical protein
gene2276	VP2221	2.0810197	Ups	heme exporter protein C
gene2269	VP2214	0.3846938	Down	VacJ lipoprotein
gene2800	VP2713	2.1758896	Ups	hypothetical protein
gene3907	VPA0676	0.0509658	Down	hypothetical protein
gene3030	VP2913	0.4630552	Down	hypothetical protein
gene3260	VPA0039	0.071761	Down	hypothetical protein
gene3579	VPA0349	0.3709604	Down	hypothetical protein
gene2044	VP1991	3.2174342	Ups	5-methyltetrahydropteroyltriglutamate--homocysteine S-methyltransferase
gene1532	VP1483	2.2850477	Ups	hypothetical protein
gene3457	VPA0227	0.2719394	Down	alkaline serine protease
gene1443	VP1394	2.5966396	Ups	VgrG protein
gene2100	VP2047	0.4776202	Down	deoxyribonuclease
gene4021	VPA0790	0.1754546	Down	YhfP protein
gene2427	VP2372	0.433828	Down	hypothetical protein
gene3604	ansB	2.9155831	Ups	L-asparaginase II
gene3247	VPA0026	0.3350882	Down	hypothetical protein
gene4709	VPA1471	0.4626491	Down	deoxyribodipyrimidine photolyase
gene4288	VPA1051	0.2766293	Down	glutathione S-transferase
gene1933	VP1884	0.2671547	Down	hypothetical protein
gene1922	VP1873	2.1647625	Ups	fumarate hydratase, class I
gene2940	VP2835	2.0830419	Ups	hypothetical protein
gene3263	VPA0042	0.2377685	Down	hypothetical protein

gene4154	VPA0923	0.2673259	Down	hypothetical protein
gene4042	VPA0811	0.4698351	Down	PTS system fructose-specific transporter subunit IIBC
gene4308	VPA1071	2.1699875	Ups	extracellular serine protease
gene3818	VPA0588	3.0292884	Ups	hypothetical protein
gene56	VP0046	0.1203081	Down	hypothetical protein
gene1251	VP1204	0.3258373	Down	hypothetical protein
gene3721	VPA0491	3.1949267	Ups	methyl-accepting chemotaxis protein
gene4385	VPA1147	2.2250478	Ups	phenylacetate-CoA ligase
gene3150	VP3016	5.9689717	Ups	hypothetical protein
gene4558	VPA1319	0.2310881	Down	IS1655 transposase
gene1055	VP1008	3.653114	Ups	outer membrane porin protein
gene2231	VP2176	0.442844	Down	aquaporin Z
gene1165	VP1118	0.4808728	Down	heat shock protein HtpX
gene1392	VP1345	3.1320264	Ups	oligopeptide ABC transporter permease
gene1110	VP1064	2.172998	Ups	potassium channels
gene3228	VPA0007	0.0351171	Down	hypothetical protein
gene4763	VPA1525	2.3367438	Ups	hypothetical protein
gene4371	VPA1133	0.1361031	Down	integral membrane protein
gene4285	VPA1048	0.097877	Down	anti-sigma F factor antagonist
gene1309	VP1262	0.2522168	Down	alkaline phosphatase
gene3679	VPA0449	0.3309847	Down	serine proteinase
gene1368	VP1321	2.5612147	Ups	dTDP-glucose 4-6-dehydratase
gene2949	VP2844	0.3965292	Down	hypothetical protein
gene4274	VPA1037	2.4321345	Ups	phosphoprotein phosphatase
gene3308	VPA0087	0.3139052	Down	transporter
gene4986	VPA1748	0.3811273	Down	chloramphenicol acetyltransferase

gene1784	VP1735	0.2911022	Down	two-component sensor
gene4489	VPA1251	0.2478705	Down	ABC transporter
gene1322	VP1275	0.3706186	Down	formimidoylglutamase
gene1706	VP1657	5.2325382	Ups	translocator protein PopB
gene2211	VP2156	0.4647125	Down	methionine sulfoxide reductase B
gene3765	VPA0535	0.3874154	Down	phosphomannomutase
gene384	VP0374	0.4405116	Down	hypothetical protein
gene3264	VPA0043	0.2830765	Down	hypothetical protein
gene917	VP0877	0.2874602	Down	LysR family transcriptional regulator
gene1562	VP1513	4.0239638	Ups	formate dehydrogenase large subunit
gene2031	VP1978	0.4055686	Down	hypothetical protein
gene1064	VP1017	0.4364203	Down	arginyl-tRNA-protein transferase
gene3172	VP3031	0.4259024	Down	carbonic anhydrase
gene1475	VP1426	0.3543148	Down	tyrosine-specific transport protein
gene320	VP0310	0.3148586	Down	hypothetical protein
gene4531	VPA1293	0.4286624	Down	antioxidant
gene252	VP0242	0.3311252	Down	hypothetical protein
gene4264	VPA1027	3.4708483	Ups	hypothetical protein
gene2055	VP2002	0.3845292	Down	hypothetical protein
gene1024	VP0977	2.2317119	Ups	hypothetical protein
gene2273	VP2218	2.1144906	Ups	cytochrome c-type biogenesis protein CcmF
gene4976	VPA1738	0.2216023	Down	tryptophan repressor binding protein
gene4040	VPA0809	0.3365469	Down	AcrB/AcrD/AcrF family transporter
gene3703	VPA0473	0.2028948	Down	hypothetical protein
gene22	VP0023	0.4706535	Down	hypothetical protein
gene2077	VP2024	0.4147148	Down	hypothetical protein

gene3755	VPA0525	2.1941828	Ups	DME family transporter
gene3834	VPA0603	0.4689593	Down	NodN-like protein
gene895	VP0855	0.0792564	Down	hypothetical protein
gene1342	VP1295	0.4232243	Down	hypothetical protein
gene3542	VPA0312	0.2998109	Down	hypothetical protein
gene1278	VP1231	2.2647732	Ups	trans-2-enoyl-CoA reductase
gene4803	VPA1564	0.3665071	Down	hypothetical protein
gene2798	VP2711	2.4285539	Ups	UTP-glucose-1-phosphate uridylyltransferase
gene4414	VPA1176	2.1390452	Ups	hypothetical protein
gene2017	VP1964	2.429211	Ups	long-chain-fatty-acid-CoA ligase
gene3687	VPA0457	0.3889784	Down	hypothetical protein
gene1369	VP1322	2.1237646	Ups	LicD1 protein
gene2113	maf	0.3880778	Down	Maf-like protein
gene2473	VP2418	0.0327265	Down	hypothetical protein
gene4038	VPA0807	0.2111421	Down	multidrug resistance protein
gene3332	VPA0111	2.0073439	Ups	hypothetical protein
gene3580	VPA0350	0.3696188	Down	ABC transporter ATP-binding protein
gene4751	VPA1513	2.8939535	Ups	aminotransferase ScrA
gene3984	VPA0753	3.2746529	Ups	hypothetical protein
gene3338	VPA0117	2.3016525	Ups	hypothetical protein
gene4804	VPA1566	0.3509452	Down	integral membrane protein
gene4099	VPA0868	0.4926723	Down	hypothetical protein
gene1705	VP1656	4.3327901	Ups	translocator protein PopD
gene1904	VP1855	2.0566543	Ups	hypothetical protein
gene4298	VPA1061	0.3316073	Down	hypothetical protein
gene1783	VP1734	0.3241714	Down	DNA-binding response regulator

gene2414	VP2359	0.3606059	Down	hypothetical protein
gene2478	VP2423	0.0215845	Down	fimbrial protein
gene1879	VP1830	0.1582156	Down	hypothetical protein
gene1323	VP1276	0.435868	Down	imidazolonepropionase
gene4556	VPA1318	0.4635556	Down	hypothetical protein
gene1728	VP1679	0.3094822	Down	hypothetical protein
gene4276	VPA1039	2.1088348	Ups	hypothetical protein
gene1393	VP1346	2.6657826	Ups	oligopeptide ABC transporter permease
gene597	VP0579	0.4661987	Down	copper homeostasis protein
gene2554	VP2497	0.4894226	Down	penicillin-binding protein 1B
gene1604	VP1555	0.0477898	Down	hypothetical protein
gene3505	VPA0275	2.6060486	Ups	flagellin
gene1641	VP1591	2.2309629	Ups	3-hydroxydecanoyl-ACP dehydratase
gene1575	VP1526	0.4698571	Down	spermidine/putrescine ABC transporter periplasmic spermidine/putrescine-binding protein
gene2264	fadI	0.4879172	Down	3-ketoacyl-CoA thiolase
gene16	VP0017	4.9892923	Ups	hypothetical protein
gene2861	VP2769	0.2436142	Down	bacterioferritin-associated ferredoxin
gene4739	VPA1501	2.098263	Ups	hypothetical protein
gene2226	VP2171	0.3999666	Down	proteinase inhibitor
gene2306	VP2251	2.1166663	Ups	FlaM
gene4270	VPA1033	3.3943054	Ups	hypothetical protein
gene4403	VPA1165	0.2440652	Down	hypothetical protein
gene3026	VP2909	0.3574713	Down	hypothetical protein
gene1326	VP1279	0.4804489	Down	hypothetical protein
gene1385	VP1338	2.6185691	Ups	ABC transporter permease

gene680	VP0662	0.4684325	Down	hypothetical protein
gene1670	VP1621	0.1416694	Down	amino acid ABC transporter permease
gene4836	VPA1598	2.4757842	Ups	N-acetylglucosamine-binding protein A
gene17	VP0018	0.4419441	Down	16 kDa heat shock protein A
gene4279	VPA1042	2.2697868	Ups	hypothetical protein
gene4838	VPA1600	0.4935682	Down	hypothetical protein
gene643	VP0625	2.8784843	Ups	hypothetical protein
gene2662	VP2596	0.3406818	Down	LysE/YggA family protein
gene852	VP0812	0.3380105	Down	hypothetical protein
gene4063	VPA0832	0.3125002	Down	chitodextrinase
gene1264	VP1217	0.4160563	Down	hypothetical protein
gene3234	VPA0013	0.0918171	Down	hypothetical protein
gene3717	VPA0487	0.2008487	Down	hypothetical protein
gene4842	VPA1604	2.0257617	Ups	tyrosine kinase
gene1282	VP1235	2.0062129	Ups	iron-containing alcohol dehydrogenase
gene1704	VP1655	0.3887075	Down	hypothetical protein
gene1002	VP0955	0.3434221	Down	ferredoxin
gene3737	VPA0507	0.110709	Down	hypothetical protein
gene3862	VPA0631	0.1420014	Down	protoheme IX farnesyltransferase
gene3688	VPA0458	2.3663318	Ups	hypothetical protein
gene385	VP0375	0.4282764	Down	lipoprotein
gene3024	VP2907	0.3221652	Down	hypothetical protein
gene3623	VPA0393	0.4019305	Down	hypothetical protein
gene654	VP0636	0.1525511	Down	outer membrane protein A
gene1962	VP1913	0.3715554	Down	hypothetical protein
gene4278	VPA1041	2.0883726	Ups	hypothetical protein

gene2056	VP2003	0.495958	Down	hypothetical protein
gene1695	VP1646	0.2822273	Down	aconitate hydratase
gene2481	VP2426	0.2851424	Down	hypothetical protein
gene4281	VPA1044	2.2029105	Ups	hypothetical protein
gene4841	VPA1603	2.1231019	Ups	phosphatase
gene3682	VPA0452	0.3249585	Down	cytochrome b561
gene3837	VPA0606	2.9854189	Ups	AraC-type regulatory protein
gene1206	VP1159	0.4379863	Down	hypothetical protein
gene406	VP0396	0.2636963	Down	hypothetical protein
gene4931	VPA1693	2.4907445	Ups	hypothetical protein
gene1516	VP1467	0.196366	Down	galactosyltransferase
gene3821	VPA0591	0.3975492	Down	hypothetical protein
gene3149	VP3015	4.4331079	Ups	hypothetical protein
gene4280	VPA1043	2.0748558	Ups	hypothetical protein
gene1590	VP1541	2.050731	Ups	cytochrome c oxidase subunit CcoP
gene2526	VP2469	0.3559158	Down	hypothetical protein
gene4077	VPA0846	2.3421986	Ups	hypothetical protein
gene4687	VPA1449	3.2927227	Ups	methyl-accepting chemotaxis protein
gene1384	VP1337	2.4158837	Ups	permease
gene3707	VPA0477	0.4032913	Down	beta-lactamase
gene3035	VP2918	2.9061277	Ups	hypothetical protein
gene3386	VPA0157	0.4003218	Down	hypothetical protein
gene3313	VPA0092	0.3788784	Down	spindolin-like protein
gene4275	VPA1038	2.094684	Ups	hypothetical protein
gene1125	VP1078	0.43966	Down	hypothetical protein
gene3835	VPA0604	3.0245774	Ups	ABC transporter periplasmic substrate-binding protein

gene101	VP0091	0.4095174	Down	permease
gene1515	VP1466	0.2786909	Down	hypothetical protein
gene309	VP0299	0.2204453	Down	hypothetical protein
gene4910	araG	2.7012705	Ups	L-arabinose transporter ATP-binding protein
gene3769	VPA0539	0.3065999	Down	cytochrome c oxidase subunit III
gene1748	VP1699	8.4789575	Ups	transcriptional regulator ExsA
gene512	VP0501	0.3545727	Down	hypothetical protein
gene1074	VP1027	2.3385141	Ups	hypothetical protein
gene2458	ebgA	0.4160349	Down	cryptic beta-D-galactosidase subunit alpha
gene3023	VP2906	0.3384944	Down	hypothetical protein
gene1935	VP1886	0.2237151	Down	hypothetical protein
gene4273	VPA1036	2.4743337	Ups	hypothetical protein
gene4257	VPA1020	0.3873743	Down	hypothetical protein
gene4607	VPA1369	0.3511544	Down	hypothetical protein
gene4829	VPA1591	2.6108278	Ups	dihydropteridine reductase
gene4956	VPA1718	0.4252042	Down	hypothetical protein
gene4315	VPA1078	0.0460952	Down	hypothetical protein
gene1991	VP1942	0.3685463	Down	diaminobutyrate-pyruvate transaminase %26 L-2,4-diaminobutyrate decarboxylase
gene4961	VPA1723	2.522677	Ups	ribosomal protein N-acetyltransferase
gene3768	VPA0538	0.3149456	Down	cytochrome C oxidase assembly protein
gene3898	VPA0667	0.2204826	Down	hypothetical protein
gene3381	VPA0152	0.4928166	Down	biopolymer transport protein ExbB-like protein
gene4235	VPA1000	0.3591632	Down	methyl-accepting chemotaxis protein
gene4541	VPA1303	0.3061549	Down	hypothetical protein
gene1259	VP1212	0.4667872	Down	DNA-binding response regulator

gene1444	VP1395	2.1411577	Ups	hypothetical protein
gene1608	VP1559	0.0655644	Down	hypothetical protein
gene4944	VPA1706	0.4476316	Down	glucuronate isomerase
gene1908	VP1859	0.0395366	Down	hypothetical protein
gene1730	VP1682	8.5618663	Ups	hypothetical protein
gene4504	VPA1266	0.4789229	Down	ATP-dependent exoDNAse subunit alpha
gene1434	VP1385	2.7240609	Ups	hypothetical protein
gene1102	VP1056	2.0812787	Ups	hypothetical protein
gene3964	VPA0733	0.3915957	Down	LysR family transcriptional regulator
gene2082	ihfB	0.2373911	Down	integration host factor subunit beta
gene3792	VPA0562	0.4730073	Down	chemotaxis transducer
gene2797	VP2710	2.0958543	Ups	LuxR family transcriptional regulator
gene4498	VPA1260	2.2846808	Ups	hypothetical protein
gene4495	VPA1257	0.1790543	Down	hypothetical protein
gene4795	VPA1557	13.701654	Ups	chemotaxis LafU protein
gene3318	VPA0097	0.3772828	Down	secretion protein
gene1377	VP1330	2.2233229	Ups	hydroxyproline-2-epimerase
gene1394	VP1347	2.6819199	Ups	oligopeptide ABC transporter periplasmic oligopeptide-binding protein
gene3793	VPA0563	0.3135038	Down	DPS family protein
gene4788	VPA1550	18.893361	Ups	flagellar distal capping protein LafB
gene3551	VPA0322	0.1531745	Down	hypothetical protein
gene1183	VP1136	0.3192648	Down	transcription regulator TxR
gene1707	VP1658	4.835797	Ups	low calcium response locus protein H
gene3235	VPA0014	0.143004	Down	hypothetical protein
gene3034	VP2917	2.4582687	Ups	hypothetical protein

gene1635	VP1586	0.3761925	Down	hypothetical protein
gene3524	VPA0294	0.4397797	Down	hypothetical protein
gene4546	VPA1308	0.3142204	Down	hypothetical protein
gene1118	VP1071	0.3266872	Down	hypothetical protein
gene1561	VP1512	3.5474761	Ups	hypothetical protein
gene3238	VPA0017	0.2080139	Down	hypothetical protein
gene4277	VPA1040	2.10709	Ups	hypothetical protein
gene4780	fliP	6.39422	Ups	flagellar biosynthesis protein FliP
gene2097	VP2044	0.3229514	Down	hypothetical protein
gene3390	VPA0161	0.4195845	Down	hypothetical protein
gene1940	VP1891	0.3478471	Down	hypothetical protein
gene3094	VP2966	0.4856503	Down	inner membrane protein
gene3497	flgE	17.340945	Ups	flagellar hook protein FlgE
gene3267	VPA0046	0.4407713	Down	methylated-DNA-protein-cysteine S-methyltransferase
gene1989	VP1940	0.3728558	Down	carboxynorspermidine decarboxylase
gene3334	VPA0113	0.3377434	Down	hypothetical protein
gene3498	VPA0268	16.899854	Ups	flagellar basal-body rod protein
gene1612	VP1563	0.2755469	Down	hypothetical protein
gene552	VP0541	0.3849791	Down	hypothetical protein
gene4545	VPA1307	0.3732615	Down	adhesion protein
gene3495	flgC	16.971178	Ups	flagellar basal body rod protein FlgC
gene4012	VPA0781	2.1937708	Ups	hypothetical protein
gene3191	VP3050	0.4762868	Down	hypothetical protein
gene3239	VPA0018	0.2426465	Down	hypothetical protein
gene1609	VP1560	0.14441	Down	hypothetical protein
gene3380	VPA0151	0.457028	Down	hypothetical protein

gene3808	VPA0579	0.4211258	Down	alkaline phosphatase
gene3433	VPA0203	0.4675649	Down	deoxyribodipyrimidine photolyase
gene1535	VP1486	0.4561727	Down	methyl-accepting chemotaxis protein
gene4351	VPA1113	0.3156876	Down	betaine aldehyde dehydrogenase
gene4544	VPA1306	0.3257716	Down	hypothetical protein
gene3379	VPA0150	0.4648995	Down	ferrichrome-iron receptor
gene3279	VPA0058	0.3476162	Down	hypothetical protein
gene2267	VP2212	2.1053313	Ups	long-chain fatty acid transport protein
gene4740	VPA1502	0.3774969	Down	carbonic anhydrase
gene1709	VP1660	6.6019257	Ups	type III secretion regulator
gene3489	VPA0259	0.3093501	Down	hypothetical protein
gene4540	VPA1302	0.4738353	Down	hypothetical protein
gene4411	VPA1173	0.2532247	Down	hypothetical protein
gene1025	VP0978	2.081	Ups	ABC transporter ATP-binding protein
gene3712	VPA0482	0.1260746	Down	outer membrane cation efflux protein
gene388	VP0378	0.4011808	Down	hypothetical protein
gene3355	VPA0134	0.2193891	Down	ABC transporter permease
gene3391	VPA0162	0.428748	Down	adhesion protein
gene4284	VPA1047	0.078503	Down	hypothetical protein
gene4699	VPA1461	2.7819699	Ups	phosphate ABC transporter periplasmic phosphate-binding protein
gene3563	VPA0333	0.4086828	Down	hypothetical protein
gene645	VP0627	3.5824465	Ups	hypothetical protein
gene4696	VPA1458	0.4136967	Down	phosphate ABC transporter ATP-binding protein
gene1878	VP1829	0.2399029	Down	RelB protein/Vco27A protein
gene3223	VPA0002	0.175903	Down	hypothetical protein

gene70	VP0060	0.4275374	Down	multidrug transmembrane resistance signal peptide protein
gene2087	VP2034	0.2883548	Down	hypothetical protein
gene4613	VPA1375	0.1804164	Down	hypothetical protein
gene4700	VPA1462	2.1000572	Ups	methyl-accepting chemotaxis protein
gene4775	VPA1537	5.6329278	Ups	flagellar hook-basal body complex protein
gene4943	VPA1705	0.4766772	Down	mannonate oxidoreductase
gene3701	VPA0471	0.4007212	Down	multidrug efflux membrane fusion protein
gene3246	VPA0025	0.4155104	Down	hypothetical protein
gene3388	VPA0159	0.4837312	Down	hypothetical protein
gene1720	VP1671	4.8717887	Ups	type III secretion system protein
gene1525	VP1476	0.1439895	Down	anti-sigma F factor antagonist
gene1383	VP1336	2.1980057	Ups	ABC transporter ATP-binding protein
gene2053	VP2000	0.4287363	Down	ribosomal-protein-alanine N-acetyltransferase
gene3802	VPA0572	0.4341427	Down	cation efflux system component
gene4794	VPA1556	13.084652	Ups	flagellar motor protein MotA
gene4082	VPA0851	0.3438098	Down	formate transporter 1
gene2192	VP2137	0.3175668	Down	hypothetical protein
gene561	VP0550	0.227561	Down	hypothetical protein
gene4791	VPA1553	12.712673	Ups	LafE protein
gene1598	VP1548	0.1378413	Down	hypothetical protein
gene4893	fecB	2.2199028	Ups	iron-dicitrate transporter substrate-binding subunit
gene2407	VP2352	0.4796444	Down	cell division protein BolA
gene4754	VPA1516	2.901609	Ups	two-component response regulator
gene3771	VPA0540	0.3442329	Down	transmembrane cytochrome oxidase complex biogenesis factor transmembrane protein

gene1607	VP1558	0.0919019	Down	bacteriophage f237 ORF7
gene73	VP0063	0.4643336	Down	thermoresistant gluconokinase
gene2039	VP1986	0.4999908	Down	hypothetical protein
gene1790	VP1741	0.4548492	Down	sodium/alanine symporter
gene4586	VPA1348	0.3768618	Down	transcriptional activator ToxR
gene3866	VPA0635	0.2656806	Down	Oxidoreductase, oxygen dependent, FAD-dependent protein
gene4302	VPA1065	0.2775811	Down	nitrate reductase, large subunit protein
gene1841	VP1792	0.4489051	Down	hypothetical protein
gene1785	VP1736	0.4418895	Down	hypothetical protein
gene1599	VP1550	0.0726906	Down	hypothetical protein
gene15	VP0016	4.8141951	Ups	hypothetical protein
gene3460	VPA0230	5.2168381	Ups	sugar phosphotransferase component II B
gene1386	VP1339	0.444314	Down	hypothetical protein
gene3281	VPA0060	0.1633673	Down	phage PP7 lysis protein
gene1605	VP1556	0.0879931	Down	bacteriophage f237 ORF5
gene1937	VP1888	0.297484	Down	hypothetical protein
gene4189	VPA0958	0.2681355	Down	NADH oxidase
gene3500	flgH	10.301863	Ups	flagellar basal body L-ring protein
gene3502	VPA0272	8.885594	Ups	flagellar protein
gene3967	VPA0736	0.4599112	Down	sensory box sensor histidine kinase/response regulator VieS
gene3491	VPA0261	2.9341649	Ups	LfgN protein
gene3499	VPA0269	13.58167	Ups	flagellar basal-body rod protein
gene4555	VPA1317	0.1674518	Down	hypothetical protein
gene4140	VPA0909	0.2385263	Down	hypothetical protein

gene3383	VPA0154	0.4881789	Down	TonB system transport protein ExbD2
gene386	VP0376	0.4434016	Down	hypothetical protein
gene71	VP0061	0.3008944	Down	multidrug transmembrane resistance signal peptide protein
gene2581	VP2524	2.1978363	Ups	type IV pilin assembly protein PilB
gene1990	VP1941	0.3033014	Down	carboxynorspermidine dehydrogenase
gene2393	VP2338	0.4736697	Down	chitinase
gene401	VP0391	0.0981805	Down	hypothetical protein
gene4947	VPA1709	0.2102516	Down	hypothetical protein
gene2412	VP2357	0.346818	Down	transcriptional activator ChrR
gene1491	VP1442	0.479173	Down	hypothetical protein
gene2275	VP2220	3.6665398	Ups	heme exporter protein D
gene4347	VPA1109	0.4238371	Down	ABC transporter ATP-binding protein
gene4283	VPA1046	0.4735798	Down	hypothetical protein
gene1796	VP1747	0.3918149	Down	amino acid transporter
gene4818	VPA1580	0.072785	Down	hypothetical protein
gene3496	flgD	12.191612	Ups	flagellar basal body rod modification protein
gene2663	VP2597	0.3977297	Down	DNA-binding protein
gene97	VP0087	0.0915996	Down	hypothetical protein
gene3240	VPA0019	0.3218878	Down	hypothetical protein
gene4779	VPA1541	8.0106595	Ups	flagellar motor switch protein
gene2314	VP2259	2.2435169	Ups	flagellin
gene1232	VP1185	0.4749882	Down	chemotaxis transducer
gene1717	VP1668	4.0249077	Ups	type III secretion system ATPase
gene1446	VP1397	2.0051012	Ups	hypothetical protein
gene1675	VP1626	0.4695396	Down	sulfite reductase, gamma subunit-like protein

gene1611	VP1562	0.2642807	Down	bacteriophage f237 ORF9
gene194	radC	2.4711881	Ups	DNA repair protein RadC
gene2416	VP2361	0.0777127	Down	hypothetical protein
gene4786	VPA1548	10.123165	Ups	lateral flagellin LafA
gene3860	VPA0629	0.0790959	Down	cytochrome o ubiquinol oxidase subunit III
gene4793	VPA1555	10.282593	Ups	flagellar-specific transcription initiation factor sigma, LafS
gene1708	VP1659	2.4277113	Ups	hypothetical protein
gene4582	VPA1344	0.0797966	Down	hypothetical protein
gene1770	VP1721	0.4236596	Down	diaminobutyrate--2-oxoglutarate aminotransferase
gene3248	VPA0027	0.0796243	Down	hypothetical protein
gene3536	VPA0306	0.1731214	Down	AnkB protein
gene3325	VPA0104	0.4285891	Down	ring-cleaving dioxygenase
gene3151	VP3017	7.8252653	Ups	transmembrane protein
gene2385	VP2330	0.3126204	Down	hypothetical protein
gene3710	VPA0480	0.3882039	Down	cation efflux system transmembrane protein
gene2854	VP2762	0.3338502	Down	hypothetical protein
gene1405	VP1356	0.398331	Down	hypothetical protein
gene3148	VP3014	5.8385842	Ups	signal peptide protein
gene4410	VPA1172	0.3439595	Down	hypothetical protein
gene1558	VP1509	2.0854819	Ups	hypothetical protein
gene3801	VPA0571	0.1309528	Down	hypothetical protein
gene3863	VPA0632	0.4546196	Down	hypothetical protein
gene3251	VPA0030	0.4230594	Down	hypothetical protein
gene3713	VPA0483	0.0840117	Down	hypothetical protein
gene3310	VPA0089	0.4903846	Down	siderophore utilization protein

gene672	VP0654	0.4516177	Down	molecular chaperone DnaJ
gene1560	VP1511	2.0614736	Ups	formate dehydrogenase-specific chaperone
gene1334	VP1287	0.3113495	Down	hypothetical protein
gene3356	VPA0135	0.1912362	Down	ABC transporter ATP-binding protein
gene3503	VPA0273	9.0558513	Ups	flagellar hook-associated protein
gene4148	VPA0917	0.304309	Down	hypothetical protein
gene3588	VPA0358	2.3294709	Ups	LuxR family transcriptional regulator
gene4479	VPA1241	0.1340662	Down	hypothetical protein
gene3204	VP3063	0.4093381	Down	DNA-binding transcriptional regulator
gene4457	VPA1219	0.4179466	Down	transcriptional regulator
gene4022	VPA0791	0.154287	Down	hypothetical protein
gene4799	VPA1561	0.1913004	Down	hypothetical protein
gene91	VP0081	0.1680596	Down	hyperosmotically inducible periplasmic protein
gene3501	flgI	7.7264301	Ups	flagellar basal body P-ring biosynthesis protein FlgA
gene3363	VPA0142	0.3148197	Down	hypothetical protein
gene1723	VP1674	4.2867215	Ups	translocation protein in type III secretion
gene850	VP0811	0.0896542	Down	hypothetical protein
gene1256	VP1209	0.3556479	Down	transcriptional regulator
gene1938	VP1889	0.3676747	Down	cold shock transcriptional regulator CspA
gene4611	VPA1373	0.4888452	Down	hypothetical protein
gene3258	VPA0037	6.7038194	Ups	hypothetical protein
gene3930	VPA0699	0.3549018	Down	hypothetical protein
gene4941	VPA1703	0.4434056	Down	small integral C4-dicarboxylate membrane transport protein
gene1473	VP1424	0.1231711	Down	hypothetical protein
gene4017	VPA0786	0.2140544	Down	hypothetical protein

gene4783	VPA1545	4.0303877	Ups	flagellar biosynthetic protein
gene4604	VPA1366	0.3263046	Down	hypothetical protein
gene3299	VPA0078	0.3131377	Down	hypothetical protein
gene3276	VPA0055	0.3441	Down	chitinase
gene2138	VP2085	0.4180809	Down	hypothetical protein
gene3504	flgL	6.5933316	Ups	flagellar hook-associated protein FlgL
gene1527	VP1478	0.4718948	Down	hypothetical protein
gene315	VP0305	0.3119107	Down	hypothetical protein
gene3488	VPA0258	0.4731828	Down	MutT/nudix family protein
gene1973	VP1924	0.27882	Down	thiol:disulfide interchange protein DsbE
gene4003	VPA0772	0.3722091	Down	hypothetical protein
gene3628	VPA0398	0.1718144	Down	hypothetical protein
gene1410	VP1362	0.1010862	Down	hypothetical protein
gene4892	VPA1654	2.5238486	Ups	ferrichrome ABC transporter permease
gene3423	VPAt06	0.1018428	Down	tRNA-Leu
gene4770	VPA1532	3.7958543	Ups	hypothetical protein
gene3512	VPA0282	0.4058502	Down	hypothetical protein
gene1591	VP1542	2.0382566	Ups	cytochrome c oxidase subunit CcoQ
gene954	VP0913	0.1615479	Down	hypothetical protein
gene4778	VPA1540	5.9349878	Ups	flagellar motor switch protein
gene1096	VP1050	0.4022306	Down	hypothetical protein
gene3465	VPA0235	0.3260604	Down	2-aminoethylphosphonate--pyruvate transaminase
gene3901	VPA0670	0.3442436	Down	hypothetical protein
gene1855	VP1806	0.1014301	Down	hypothetical protein
gene4529	VPA1291	0.1890199	Down	hypothetical protein
gene3700	VPA0470	0.2455845	Down	multidrug efflux system transmembrane protein

gene3639	VPA0409	0.2916684	Down	plasma membrane protein involved in salt tolerance
gene4789	VPA1551	8.580954	Ups	LafC protein
gene1729	VP1680	2.9726504	Ups	hypothetical protein
gene4258	VPA1021	0.4664497	Down	hypothetical protein
gene1544	VP1495	2.1575028	Ups	hypothetical protein
gene1716	VP1667	3.3273194	Ups	outer membrane protein PopN
gene3702	VPA0472	0.4689015	Down	long-chain fatty acid transport protein
gene4909	araH	2.3488315	Ups	L-arabinose transporter permease
gene1640	VP1592	0.3152256	Down	hypothetical protein
gene3613	VPA0383	2.4253467	Ups	hypothetical protein
gene2094	VP2041	0.1613232	Down	hypothetical protein
gene3772	VPA0542	0.401333	Down	hypothetical protein
gene4921	VPA1683	0.4641441	Down	alkyl hydroperoxide reductase
gene304	VP0294	0.2106585	Down	hypothetical protein
gene4356	VPA1118	2.0115963	Ups	3-hydroxyisobutyrate dehydrogenase
gene3237	VPA0015	0.2425241	Down	ABC transporter ATP-binding protein
gene1834	VP1785	0.2740825	Down	hypothetical protein
gene4782	VPA1544	3.3433555	Ups	flagellar biosynthetic protein FliR
gene4908	VPA1670	0.4641759	Down	collagenase family protease
gene2842	VP2750	2.5328466	Ups	fimbrial assembly protein PilM
gene1565	VP1516	3.4914032	Ups	hypothetical protein
gene4532	VPA1294	3.3092355	Ups	DamX-like protein
gene3410	VPA0181	7.2240269	Ups	hypothetical protein
gene1166	VP1119	0.3024658	Down	transcriptional regulator
gene3305	VPA0084	0.4196952	Down	transporter protein
gene2459	ebgC	0.4791535	Down	cryptic beta-D-galactosidase subunit beta

gene3091	VP2963	0.1647734	Down	hypothetical protein
gene1600	VP1551	0.1216491	Down	bacteriophage f237 ORF1
gene1497	VP1448	0.3520145	Down	anaerobic dimethyl sulfoxide reductase subunit B
gene1144	VP1097	0.2845717	Down	ATP-dependent dsDNA exonuclease SbcC
gene851	VP0810	0.448223	Down	PTS system mannose-specific, factor IIC
gene4843	VPA1605	0.4112085	Down	hypothetical protein
gene714	VP0696	2.3317634	Ups	minor nuclease C1B isoform
gene3456	VPA0226	2.1817389	Ups	lecithin-dependent hemolysin (LDH)
gene1602	VP1553	0.1308215	Down	bacteriophage f237 ORF3
gene2043	VP1990	0.3403114	Down	hypothetical protein
gene3494	flgB	8.5541977	Ups	flagellar basal-body rod protein FlgB
gene1732	VP1683	3.3114302	Ups	hypothetical protein
gene4548	VPA1310	0.4140972	Down	hypothetical protein
gene3774	VPA0544	0.4942396	Down	protoheme IX farnesyltransferase
gene4784	VPA1546	3.4815337	Ups	flagellar biosynthesis protein
gene1597	VP1549	0.3488041	Down	bacteriophage f237 ORF10
gene993	VP0946	0.389997	Down	cysteine synthase/cystathionine beta-synthase family protein
gene1089	VP1042	0.1659179	Down	hypothetical protein
gene2952	VP2847	0.3097204	Down	hypothetical protein
gene3741	VPA0511	2.0778711	Ups	methyl-accepting chemotaxis protein
gene394	VP0384	3.1895611	Ups	hypothetical protein
gene2413	VP2358	0.497117	Down	RNA polymerase sigma factor
gene4297	VPA1060	0.2848549	Down	two-component response regulatory protein
gene1719	VP1670	3.5075901	Ups	translocation protein in type III secretion
gene1499	VP1450	0.2783064	Down	component of anaerobic dehydrogenase

gene976	VP0935	0.3944656	Down	hypothetical protein
gene3929	VPA0698	0.4603197	Down	arylsulfatase regulator
gene4350	VPA1112	0.371382	Down	choline dehydrogenase
gene1205	VP1158	0.4373576	Down	hypothetical protein
gene1477	VP1428	0.2046014	Down	hypothetical protein
gene3593	VPA0363	0.4861012	Down	efflux protein
gene381	VP0371	0.3850331	Down	hypothetical protein
gene4516	VPA1278	0.4806299	Down	hypothetical protein
gene656	VP0638	0.3136137	Down	resolvase
gene1095	VP1049	0.4848751	Down	hypothetical protein
gene4773	fliG	4.479819	Ups	flagellar motor switch protein G
gene4601	VPA1363	0.1354357	Down	chaperone
gene4913	araD	2.7151063	Ups	L-ribulose-5-phosphate 4-epimerase
gene1202	VP1155	0.4287752	Down	hypothetical protein
gene4211	VPA0980	0.436849	Down	hypothetical protein
gene4491	VPA1253	0.4343891	Down	hypothetical protein
gene1734	VP1685	0.1536333	Down	hypothetical protein
gene798	VP0759	0.1381168	Down	hypothetical protein
gene3865	VPA0634	0.4619491	Down	NADH-dependent flavin oxidoreductase
gene4844	VPA1606	0.1383967	Down	hypothetical protein
gene4806	VPA1568	0.4868316	Down	pyruvate formate-lyase 3 activating enzyme
gene4355	fabG	2.2781662	Ups	3-ketoacyl-ACP reductase
gene1971	VP1922	0.4531864	Down	hypothetical protein
gene1408	VP1359	0.1421957	Down	hypothetical protein
gene1288	VP1242	0.1419963	Down	hypothetical protein
gene3859	VPA0628	0.4793222	Down	cytochrome o ubiquinol oxidase subunit I

gene3978	VPA0747	2.0267247	Ups	MSHA pilin protein MshA
gene4798	VPA1560	0.1757629	Down	hypothetical protein
gene4774	fliF	4.3034206	Ups	flagellar MS-ring protein
gene4792	VPA1554	6.3640593	Ups	LafF protein
gene1603	VP1554	0.1495186	Down	bacteriophage f237 ORF4
gene4652	VPA1414	0.3399414	Down	hypothetical protein
gene4895	VPA1657	2.8122612	Ups	ferric siderophore receptor-like protein
gene409	VP0399	0.1727475	Down	transcriptional regulator
gene4567	VPA1329	0.2808877	Down	traA protein
gene1620	VP1571	0.147583	Down	hypothetical protein
gene1671	VP1622	0.396136	Down	amino acid ABC transporter permease
gene1835	VP1786	0.451696	Down	hypothetical protein

Table S3. Differentially expressed genes involved in biofilm formation, virulence, and regulatory functions

Gene ID	Gene name	Fold change (smooth/wrinkly)	Product
**Synthesis of Syp exopolysaccharide**			
VP1466	*sypJ*	0.279	hypothetical protein
VP1467	*sypI*	0.196	galactosyltransferase
VP1468	*sypH*	0.060	hexosyltransferase
VP1469	*sypG*	0.033	LuxO repressor protein
VP1472	*sypE*	0.107	phosphorelay protein
VP1473	*sypD*	0.082	capsular polysaccharide biosynthesis
VP1474	*sypC*	0.050	capsule transport protein OtnA
VP1475	*sypB*	0.052	hypothetical protein
VP1476	*sypA*	0.144	anti-sigma F factor antagonist
**Type IV pilus**			
VP2523	*pilA*	6.375	type IV pilin PilA
VP2524	*pilB*	2.198	type IV pilin assembly protein PilB
**c-di-GMP metabolism**			
VP0376		0.443	EAL-only
VP0699		2.354	GGDEF-only
VP1483		2.285	GGDEF-only
VP1979		0.045	EAL-only
VP2366		0.054	GGDEF-only
VP2979		3.044	EAL-only
VPA0059		0.289	GGDEF-only
VPA0476		0.098	GGDEF-only
VPA0556		3.515	GGDEF-only
VPA0609		0.157	GGDEF-EAL
VPA0818		4.185	EAL-only
VPA0846		2.342	EAL-only
VPA0869		0.188	GGDEF-EAL

VPA0927		0.291	GGDEF-only
VPA1176		2.139	GGDEF-EAL
**Flagellar synthesis**			
Lateral flagella			
VPA0261	*flgN*	2.934	FlgN protein
VPA0264	*flgB*	8.554	flagellar basal-body rod protein FlgB
VPA0265	*flgC*	16.971	flagellar basal body rod protein FlgC
VPA0266	*flgD*	12.192	flagellar basal body rod modification protein
VPA0267	*flgE*	17.341	flagellar hook protein FlgE
VPA0268	*flgF*	16.900	flagellar basal-body rod protein
VPA0269	*flgG*	13.582	flagellar basal-body rod protein
VPA0270	*flgH*	10.302	flagellar basal body L-ring protein
VPA0271	*flgI*	7.726	flagellar basal body P-ring biosynthesis protein FlgA
VPA0272	*flgJ*	8.886	flagellar protein
VPA0273	*flgK*	9.056	flagellar hook-associated protein
VPA0274	*flgL*	6.593	flagellar hook-associated protein FlgL
VPA0275	*flgU*	2.606	flagellin
VPA1532	*fliJ*	3.796	hypothetical protein
VPA1535	*fliG*	4.480	flagellar motor switch protein G
VPA1536	*fliF*	4.303	flagellar MS-ring protein
VPA1537	*fliE*	5.633	flagellar hook-basal body complex protein
VPA1540	*fliM*	5.935	flagellar motor switch protein
VPA1541	*fliN*	8.011	flagellar motor switch protein
VPA1542	*fliP*	6.394	flagellar biosynthesis protein FliP
VPA1544	*fliR*	3.343	flagellar biosynthetic protein FliR
VPA1545	*flhB*	4.030	flagellar biosynthetic protein
VPA1546	*flhA*	3.482	flagellar biosynthesis protein
VPA1548	*lafA*	10.123	lateral flagellin LafA
VPA1550	*fliD*	18.893	flagellar distal capping protein LafB
VPA1551	*fliS*	8.581	LafC protein

VPA1553	*fliK*	12.713	LafE protein
VPA1554	*fliL*	6.364	LafF protein
VPA1555	*fliA*	10.283	flagellar-specific transcription initiation factor sigma, LafS
VPA1556	*motA*	13.085	flagellar motor protein MotA
VPA1557	*motB*	13.702	chemotaxis LafU protein
Polar flagellum			
VP2251	*flaM*	2.117	FlaM
VP2259	*flaB*	2.244	flagellin
**T3SS1**			
VP1656	*vopD*	4.333	translocator protein PopD
VP1657	*vopB*	5.233	translocator protein PopB
VP1658	*vcrH*	4.836	low calcium response locus protein H
VP1659	*vcrV*	2.428	hypothetical protein
VP1660	*vcrG*	6.602	type III secretion regulator
VP1667	*vopN*	3.327	outer membrane protein PopN
VP1668	*vscN*	4.025	type III secretion system ATPase
VP1670	*vscP*	3.508	translocation protein in type III secretion
VP1671	*vscQ*	4.872	type III secretion system protein
VP1674	*vscT*	4.287	translocation protein in type III secretion
VP1677		0.153	hypothetical protein
VP1678		0.242	dienelactone hydrolase
VP1679		0.309	hypothetical protein
VP1680	*vopQ*	2.973	hypothetical protein
VP1682	*vecA*	8.562	hypothetical protein
VP1683	*vopR*	3.311	hypothetical protein
VP1685		0.154	hypothetical protein
VP1699	*exsA*	8.479	transcriptional regulator ExsA
**Vp-PAI**			
VPA1317		0.167	hypothetical protein
VPA1318		0.464	hypothetical protein
VPA1319		0.231	IS1655 transposase

VPA1329		0.281	traA protein
VPA1344		0.080	hypothetical protein
VPA1348	*vtrB*	0.377	transcriptional activator ToxR
VPA1363		0.135	chaperone
VPA1366		0.326	hypothetical protein
VPA1369		0.351	hypothetical protein
VPA1373		0.489	hypothetical protein
VPA1375		0.180	hypothetical protein
**T6SS1**			
VP1388		3.687	hypothetical protein
VP1391	*vasH*	5.273	transcriptional regulator
VP1392	*clpV1*	6.827	ClpA/B-type protease
VP1393	*hcp1*	5.221	BfdA protein
VP1394	*vgrG1*	2.597	VgrG protein
VP1395		2.141	hypothetical protein
VP1397		2.005	hypothetical protein
VP1400		6.750	hypothetical protein
VP1401		8.704	hypothetical protein
VP1402	*vipA1*	8.654	hypothetical protein
VP1403	*vipB1*	8.860	hypothetical protein
VP1404		7.792	hypothetical protein
VP1405		6.625	hypothetical protein
VP1406		2.858	hypothetical protein
VP1407		5.234	transcriptional regulator
VP1408	*icmf*	5.200	IcmF-like protein
VP1409		5.230	hypothetical protein
VP1410		10.611	hypothetical protein
VP1411	*fha1*	9.212	hypothetical protein
VP1412		6.963	hypothetical protein
VP1413		10.892	hypothetical protein
VP1414	*ompA*	12.124	hypothetical protein
VP1416		3.645	hypothetical protein

VP1417		3.329	hypothetical protein
**T6SS2**			
VPA1024		4.513	hypothetical protein
VPA1025		4.259	hypothetical protein
VPA1026	*vgrG2*	3.557	hypothetical protein
VPA1027	*hcp2*	3.471	hypothetical protein
VPA1028	*clpV2*	5.967	ClpA/B-type chaperone
VPA1029		5.590	hypothetical protein
VPA1030		4.740	hypothetical protein
VPA1031		5.102	hypothetical protein
VPA1032		4.476	hypothetical protein
VPA1033		3.394	hypothetical protein
VPA1034	*vipB2*	5.120	hypothetical protein
VPA1035	*vipA2*	4.427	hypothetical protein
VPA1036		2.474	hypothetical protein
VPA1037		2.432	phosphoprotein phosphatase
VPA1038		2.095	hypothetical protein
VPA1039		2.109	hypothetical protein
VPA1040		2.107	hypothetical protein
VPA1041		2.088	hypothetical protein
VPA1042		2.270	hypothetical protein
VPA1043		2.075	hypothetical protein
VPA1044		2.203	hypothetical protein
VPA1046		0.474	hypothetical protein
**Extracellular proteases**			
VP1340	*vppC*	0.030	collagenase
VPA0227	*prtA*	0.272	alkaline serine protease
VPA0449		0.331	serine proteinase
VPA1071		2.170	extracellular serine protease
**Putative regulators**			
VP0040		3.315	TetR family of transcriptional regulator
VP0358		2.395	DeoR family transcriptional regulator

VP0367		2.613	DNA-binding transcriptional regulator DhaR
VP0399		6.497	LuxR family transcriptional regulator
VP0877		2.985	AraC-type regulatory protein
VP1119		0.302	transcriptional regulator
VP1136		0.319	transcription regulator TxR
VP1209		0.356	transcriptional regulator
VP1212		2.902	two-component response regulator
VP1649		2.329	LuxR family transcriptional regulator
VP1734		3.000	LuxR family transcriptional regulator
VP1889		2.096	LuxR family transcriptional regulator
VP1993		0.165	GntR family transcriptional regulator
VP2139		0.111	hypothetical protein
VP2183		0.042	response regulator
VP2424		0.173	transcriptional regulator
VP2427		0.031	LysR family transcriptional regulator
VP2710		0.441	LysR family transcriptional regulator
VP2762	*aphA*	0.221	PadR-family DNA-binding regulator
VP3063		0.334	hypothetical protein
VPA0105		0.477	transcriptional regulator
VPA0183		2.657	C4-dicarboxylate transport transcriptional regulatory protein
VPA0200		0.189	transcriptional regulator
VPA0358		0.010	LuxR family transcriptional regulator
VPA0388		0.287	LysR family transcriptional regulator
VPA0456		0.467	DNA-binding response regulator
VPA0507		0.324	DNA-binding response regulator
VPA0593		0.240	transcriptional regulator
VPA0606	*qsvR*	0.030	AraC family transcriptional regulator
VPA0619		0.368	cold shock transcriptional regulator CspA
VPA0684		0.167	arylsulfatase regulator
VPA0698		0.460	arylsulfatase regulator

VPA0733		0.372	TetR family transcriptional regulator
VPA0736		0.460	sensory box sensor histidine kinase/response regulator VieS
VPA0740		0.392	LysR family transcriptional regulator
VPA0824		22.917	regulatory protein UhpC
VPA1049		0.288	hypothetical protein
VPA1060		0.285	two-component response regulatory protein
VPA1130		0.037	sensory box sensor histidine kinase/response regulator
VPA1219		0.409	DNA-binding transcriptional regulator
VPA1446	*cpsQ*	0.418	transcriptional regulator
VPA1447	*cpsS*	0.297	transcriptional regulator
VPA1516		0.119	LysR family transcriptional regulator
VPA1687		0.002	LysR family transcriptional regulator
**Mfp proteins**			
VPA1445	*mfpA*	8.774	secreted calcium-binding protein
VPA1444	*mfpB*	10.608	transport protein
VPA1443	*mfpC*	6.668	protein secretion protein
**Anti-oxidative genes**			
VPA0768	*katG1*	0.190	catalase/peroxidase
VPA0453	*katG2*	0.112	catalase/peroxidase
VPA1683	*ahpC1*	0.464	alkyl hydroperoxide reductase
**Outer membrane proteins**			
VP0636		0.153	outer membrane protein A
VP0764	*ompA1*	5.085	outer membrane protein OmpA
VP1008		3.653	outer membrane porin protein
VP1218		0.024	outer membrane protein
VPA0096	*ompW*	2.227	outer membrane protein W
VPA0248		6.791	outer membrane protein OmpA
VPA0527		7.500	outer membrane protein N
VPA1186	*ompA2*	0.211	outer membrane protein OmpA
VPA1579		0.023	outer membrane protein

Table S4. Small non-coding RNA identified in this study

sRNA_ID	Chr	Start	End	Strand	Type	Antisense
sRNA1	NC_004603.1	2576	2953	+	cis-encoded	antisense: VP_RS00015
sRNA2	NC_004603.1	3104	3424	+	cis-encoded	antisense: VP_RS00015
sRNA3	NC_004603.1	3529	4029	+	cis-encoded	antisense: VP_RS00020 VP_RS00015
sRNA4	NC_004603.1	4252	4432	+	cis-encoded	antisense: VP_RS00025
sRNA5	NC_004603.1	4493	4909	+	cis-encoded	antisense: VP_RS00025 VP_RS00030
sRNA6	NC_004603.1	6799	7235	+	cis-encoded	antisense: VP_RS00045
sRNA7	NC_004603.1	19501	19751	+	cis-encoded	antisense: VP_RS00090
sRNA8	NC_004603.1	20012	20084	+	cis-encoded	antisense: VP_RS00090
sRNA9	NC_004603.1	20340	20544	+	cis-encoded	antisense: VP_RS00090
sRNA10	NC_004603.1	20974	21068	+	cis-encoded	antisense: VP_RS00090
sRNA11	NC_004603.1	21645	21938	+	cis-encoded	antisense: VP_RS00095
sRNA12	NC_004603.1	22038	22330	+	cis-encoded	antisense: VP_RS00095
sRNA13	NC_004603.1	24871	24544	-	cis-encoded	antisense: VP_RS00120
sRNA14	NC_004603.1	26952	27867	+	cis-encoded	antisense: VP_RS00130
sRNA15	NC_004603.1	27926	30246	+	cis-encoded	antisense: VP_RS00130 VP_RS00135
sRNA16	NC_004603.1	33757	33620	-	cis-encoded	antisense: VP_RS00155
sRNA17	NC_004603.1	43169	43450	+	cis-encoded	antisense: VP_RS00200
sRNA18	NC_004603.1	44033	44154	+	cis-encoded	antisense: VP_RS00200
sRNA19	NC_004603.1	44209	44358	+	cis-encoded	antisense: VP_RS00200
sRNA20	NC_004603.1	44638	45119	+	cis-encoded	antisense:

						VP_RS00200
sRNA21	NC_004603.1	45570	45942	+	cis-encoded	antisense: VP_RS00205
sRNA22	NC_004603.1	53670	52486	-	cis-encoded	antisense: VP_RS00255
sRNA23	NC_004603.1	56101	53917	-	cis-encoded	antisense: VP_RS00265 VP_RS00255 VP_RS00260
sRNA24	NC_004603.1	76780	77087	+	cis-encoded	antisense: VP_RS00355
sRNA25	NC_004603.1	77145	77619	+	cis-encoded	antisense: VP_RS00355
sRNA26	NC_004603.1	87694	88286	+	cis-encoded	antisense: VP_RS00395 VP_RS00390
sRNA27	NC_004603.1	89219	88767	-	cis-encoded	antisense: VP_RS00400 VP_RS00405
sRNA28	NC_004603.1	90819	91047	+	cis-encoded	antisense: VP_RS00415
sRNA29	NC_004603.1	91578	92121	+	cis-encoded	antisense: VP_RS00415
sRNA30	NC_004603.1	109003	108642	-	cis-encoded	antisense: VP_RS00510 VP_RS00515
sRNA31	NC_004603.1	112181	111391	-	cis-encoded	antisense: VP_RS00530 VP_RS00525
sRNA32	NC_004603.1	118478	117731	-	cis-encoded	antisense: VP_RS00545
sRNA33	NC_004603.1	129593	130882	+	cis-encoded	antisense: VP_RS00595
sRNA34	NC_004603.1	139298	140924	+	cis-encoded	antisense: VP_RS00635
sRNA35	NC_004603.1	144622	144402	-	cis-encoded	antisense: VP_RS00655
sRNA36	NC_004603.1	156055	156474	+	cis-encoded	antisense: VP_RS00720
sRNA37	NC_004603.1	173604	173907	+	cis-encoded	antisense: VP_RS00790
sRNA38	NC_004603.1	180265	180512	+	cis-encoded	antisense: VP_RS00830
sRNA39	NC_004603.1	180771	180982	+	cis-encoded	antisense:

						VP_RS00830
sRNA40	NC_004603.1	181483	182249	+	cis-encoded	antisense: VP_RS00830
sRNA41	NC_004603.1	199010	198479	-	cis-encoded	antisense: VP_RS00910 VP_RS00915
sRNA42	NC_004603.1	232226	232102	-	cis-encoded	antisense: VP_RS01080
sRNA43	NC_004603.1	232925	232705	-	cis-encoded	antisense: VP_RS01080
sRNA44	NC_004603.1	235990	235716	-	cis-encoded	antisense: VP_RS01090
sRNA45	NC_004603.1	236338	236153	-	cis-encoded	antisense: VP_RS01095
sRNA46	NC_004603.1	253623	254369	+	cis-encoded	antisense: VP_RS01175
sRNA47	NC_004603.1	258506	258614	+	cis-encoded	antisense: VP_RS01210
sRNA48	NC_004603.1	263223	263877	+	cis-encoded	antisense: VP_RS01235
sRNA49	NC_004603.1	282077	282440	+	cis-encoded	antisense: VP_RS01395
sRNA50	NC_004603.1	292031	288685	-	cis-encoded	antisense: VP_RS01430 VP_RS01440 VP_RS01435 VP_RS01425
sRNA51	NC_004603.1	307679	308110	+	cis-encoded	antisense: VP_RS01515
sRNA52	NC_004603.1	308228	309225	+	cis-encoded	antisense: VP_RS01520
sRNA53	NC_004603.1	318634	319482	+	cis-encoded	antisense: VP_RS01560
sRNA54	NC_004603.1	322694	322963	+	cis-encoded	antisense: VP_RS01580
sRNA55	NC_004603.1	324230	323631	-	cis-encoded	antisense: VP_RS01595 VP_RS01590
sRNA56	NC_004603.1	332792	332994	+	cis-encoded	antisense: VP_RS01640
sRNA57	NC_004603.1	333306	333475	+	cis-encoded	antisense: VP_RS01640
sRNA58	NC_004603.1	346140	345060	-	cis-encoded	antisense: VP_RS01695

						VP_RS01690
sRNA59	NC_004603.1	349222	349055	-	cis-encoded	antisense: VP_RS01700
sRNA60	NC_004603.1	353639	354723	+	cis-encoded	antisense: VP_RS01720
sRNA61	NC_004603.1	361518	361576	+	cis-encoded	antisense: VP_RS01750
sRNA62	NC_004603.1	393905	394089	+	cis-encoded	antisense: VP_RS01890
sRNA63	NC_004603.1	395548	395779	+	cis-encoded	antisense: VP_RS01890
sRNA64	NC_004603.1	404116	405206	+	cis-encoded	antisense: VP_RS01930
sRNA65	NC_004603.1	405413	405995	+	cis-encoded	antisense: VP_RS01930 VP_RS01935
sRNA66	NC_004603.1	407904	408534	+	cis-encoded	antisense: VP_RS01940 VP_RS01945
sRNA67	NC_004603.1	438600	438713	+	cis-encoded	antisense: VP_RS02075
sRNA68	NC_004603.1	439677	439795	+	cis-encoded	antisense: VP_RS02075
sRNA69	NC_004603.1	440183	440565	+	cis-encoded	antisense: VP_RS02080
sRNA70	NC_004603.1	452626	452130	-	cis-encoded	antisense: VP_RS02155 VP_RS02150
sRNA71	NC_004603.1	471191	471067	-	cis-encoded	antisense: VP_RS02230
sRNA72	NC_004603.1	475324	475084	-	cis-encoded	antisense: VP_RS02245
sRNA73	NC_004603.1	478924	475584	-	cis-encoded	antisense: VP_RS02245 VP_RS02250
sRNA74	NC_004603.1	488703	488796	+	cis-encoded	antisense: VP_RS02305
sRNA75	NC_004603.1	489701	489808	+	cis-encoded	antisense: VP_RS02305
sRNA76	NC_004603.1	490708	491026	+	cis-encoded	antisense: VP_RS02305
sRNA77	NC_004603.1	491249	491652	+	cis-encoded	antisense: VP_RS02305

sRNA78	NC_004603.1	491890	491991	+	cis-encoded	antisense: VP_RS02305
sRNA79	NC_004603.1	505844	505964	+	cis-encoded	antisense: VP_RS02340
sRNA80	NC_004603.1	513523	513660	+	cis-encoded	antisense: VP_RS02375
sRNA81	NC_004603.1	529667	529556	-	cis-encoded	antisense: VP_RS02450
sRNA82	NC_004603.1	531094	530560	-	cis-encoded	antisense: VP_RS02450
sRNA83	NC_004603.1	531916	531687	-	cis-encoded	antisense: VP_RS02455
sRNA84	NC_004603.1	546422	546662	+	cis-encoded	antisense: VP_RS02525
sRNA85	NC_004603.1	551093	550887	-	cis-encoded	antisense: VP_RS02540
sRNA86	NC_004603.1	552310	551405	-	cis-encoded	antisense: VP_RS02540
sRNA87	NC_004603.1	557369	557746	+	cis-encoded	antisense: VP_RS02570
sRNA88	NC_004603.1	558111	558701	+	cis-encoded	antisense: VP_RS02570
sRNA89	NC_004603.1	567477	567835	+	cis-encoded	antisense: VP_RS02620
sRNA90	NC_004603.1	568232	568856	+	cis-encoded	antisense: VP_RS02620
sRNA91	NC_004603.1	573657	573906	+	cis-encoded	antisense: VP_RS02645
sRNA92	NC_004603.1	576258	576495	+	cis-encoded	antisense: VP_RS02655
sRNA93	NC_004603.1	581335	580814	-	cis-encoded	antisense: VP_RS02670
sRNA94	NC_004603.1	581843	581613	-	cis-encoded	antisense: VP_RS02675
sRNA95	NC_004603.1	606415	607028	+	cis-encoded	antisense: VP_RS02795
sRNA96	NC_004603.1	609001	608796	-	cis-encoded	antisense: VP_RS02805
sRNA97	NC_004603.1	620491	620784	+	cis-encoded	antisense: VP_RS02850
sRNA98	NC_004603.1	623546	622402	-	cis-encoded	antisense: VP_RS02865

sRNA99	NC_004603.1	624593	623648	-	cis-encoded	antisense: VP_RS02870 VP_RS02880 VP_RS02875
sRNA100	NC_004603.1	628929	628449	-	cis-encoded	antisense: VP_RS02900
sRNA101	NC_004603.1	632998	632593	-	cis-encoded	antisense: VP_RS02920
sRNA102	NC_004603.1	634564	633900	-	cis-encoded	antisense: VP_RS02925
sRNA103	NC_004603.1	636174	635837	-	cis-encoded	antisense: VP_RS02935
sRNA104	NC_004603.1	644470	644142	-	cis-encoded	antisense: VP_RS02965
sRNA105	NC_004603.1	648364	648174	-	cis-encoded	antisense: VP_RS02980
sRNA106	NC_004603.1	650152	651707	+	cis-encoded	antisense: VP_RS02990
sRNA107	NC_004603.1	652391	653247	+	cis-encoded	antisense: VP_RS02995
sRNA108	NC_004603.1	656931	657362	+	cis-encoded	antisense: VP_RS03015
sRNA109	NC_004603.1	657521	657850	+	cis-encoded	antisense: VP_RS03015
sRNA110	NC_004603.1	659806	659569	-	cis-encoded	antisense: VP_RS03020
sRNA111	NC_004603.1	674401	675287	+	cis-encoded	antisense: VP_RS03090 VP_RS03095
sRNA112	NC_004603.1	685966	684441	-	cis-encoded	antisense: VP_RS03140
sRNA113	NC_004603.1	700944	701728	+	cis-encoded	antisense: VP_RS03205
sRNA114	NC_004603.1	702725	702998	+	cis-encoded	antisense: VP_RS03215
sRNA115	NC_004603.1	703342	703587	+	cis-encoded	antisense: VP_RS03215
sRNA116	NC_004603.1	703903	704003	+	cis-encoded	antisense: VP_RS03215
sRNA117	NC_004603.1	711143	710983	-	cis-encoded	antisense: VP_RS03245
sRNA118	NC_004603.1	716242	715838	-	cis-encoded	antisense: VP_RS03270 VP_RS03275

sRNA119	NC_004603.1	716604	716419	-	cis-encoded	antisense: VP_RS03280
sRNA120	NC_004603.1	738595	739051	+	cis-encoded	antisense: VP_RS03380
sRNA121	NC_004603.1	751888	752312	+	cis-encoded	antisense: VP_RS03435
sRNA122	NC_004603.1	752555	752665	+	cis-encoded	antisense: VP_RS03440
sRNA123	NC_004603.1	753413	753545	+	cis-encoded	antisense: VP_RS03445
sRNA124	NC_004603.1	762402	762482	+	cis-encoded	antisense: VP_RS03490
sRNA125	NC_004603.1	762653	762838	+	cis-encoded	antisense: VP_RS03490
sRNA126	NC_004603.1	762962	763189	+	cis-encoded	antisense: VP_RS03490
sRNA127	NC_004603.1	766369	766584	+	cis-encoded	antisense: VP_RS03505
sRNA128	NC_004603.1	766821	767188	+	cis-encoded	antisense: VP_RS03505
sRNA129	NC_004603.1	767606	767855	+	cis-encoded	antisense: VP_RS03510 VP_RS03515
sRNA130	NC_004603.1	768486	768605	+	cis-encoded	antisense: VP_RS03515
sRNA131	NC_004603.1	768901	769019	+	cis-encoded	antisense: VP_RS03520
sRNA132	NC_004603.1	769435	770151	+	cis-encoded	antisense: VP_RS03520
sRNA133	NC_004603.1	774992	775518	+	cis-encoded	antisense: VP_RS03640
sRNA134	NC_004603.1	776194	776501	+	cis-encoded	antisense: VP_RS03645
sRNA135	NC_004603.1	776814	777858	+	cis-encoded	antisense: VP_RS03650 VP_RS03655
sRNA136	NC_004603.1	802088	801917	-	cis-encoded	antisense: VP_RS03760
sRNA137	NC_004603.1	807911	808112	+	cis-encoded	antisense: VP_RS03795
sRNA138	NC_004603.1	813017	811806	-	cis-encoded	antisense: VP_RS03820 VP_RS03830 VP_RS03825

sRNA139	NC_004603.1	814368	813642	-	cis-encoded	antisense: VP_RS03835
sRNA140	NC_004603.1	824900	823981	-	cis-encoded	antisense: VP_RS03875 VP_RS03880
sRNA141	NC_004603.1	827117	827715	+	cis-encoded	antisense: VP_RS03890
sRNA142	NC_004603.1	828065	828523	+	cis-encoded	antisense: VP_RS03895
sRNA143	NC_004603.1	828712	829107	+	cis-encoded	antisense: VP_RS03895
sRNA144	NC_004603.1	829205	829361	+	cis-encoded	antisense: VP_RS03895
sRNA145	NC_004603.1	829612	829862	+	cis-encoded	antisense: VP_RS03900
sRNA146	NC_004603.1	830276	831143	+	cis-encoded	antisense: VP_RS03905
sRNA147	NC_004603.1	833521	833354	-	cis-encoded	antisense: VP_RS03920
sRNA148	NC_004603.1	839049	839183	+	cis-encoded	antisense: VP_RS03940
sRNA149	NC_004603.1	852276	851672	-	cis-encoded	antisense: VP_RS04015 VP_RS04010
sRNA150	NC_004603.1	855585	856347	+	cis-encoded	antisense: VP_RS04030
sRNA151	NC_004603.1	856481	857079	+	cis-encoded	antisense: VP_RS04030
sRNA152	NC_004603.1	859187	859471	+	cis-encoded	antisense: VP_RS04040
sRNA153	NC_004603.1	859830	860419	+	cis-encoded	antisense: VP_RS04045 VP_RS04040
sRNA154	NC_004603.1	866813	867119	+	cis-encoded	antisense: VP_RS04070
sRNA155	NC_004603.1	867188	867388	+	cis-encoded	antisense: VP_RS04075
sRNA156	NC_004603.1	872575	873811	+	cis-encoded	antisense: VP_RS04105
sRNA157	NC_004603.1	908678	908243	-	cis-encoded	antisense: VP_RS04260
sRNA158	NC_004603.1	946107	945667	-	cis-encoded	antisense: VP_RS04435 VP_RS04430

sRNA159	NC_004603.1	953286	952022	-	cis-encoded	antisense: VP_RS04465 VP_RS04470
sRNA160	NC_004603.1	954083	953512	-	cis-encoded	antisense: VP_RS04475 VP_RS04470
sRNA161	NC_004603.1	954894	954479	-	cis-encoded	antisense: VP_RS04475
sRNA162	NC_004603.1	959142	959048	-	cis-encoded	antisense: VP_RS04490
sRNA163	NC_004603.1	1005858	1006048	+	cis-encoded	antisense: VP_RS04715
sRNA164	NC_004603.1	1029229	1031587	+	cis-encoded	antisense: VP_RS04820 VP_RS04825
sRNA165	NC_004603.1	1031744	1034060	+	cis-encoded	antisense: VP_RS04830 VP_RS04825
sRNA166	NC_004603.1	1038185	1040203	+	cis-encoded	antisense: VP_RS04855
sRNA167	NC_004603.1	1060607	1060832	+	cis-encoded	antisense: VP_RS04940
sRNA168	NC_004603.1	1061993	1061887	-	cis-encoded	antisense: VP_RS04950
sRNA169	NC_004603.1	1063579	1062431	-	cis-encoded	antisense: VP_RS04950
sRNA170	NC_004603.1	1064007	1063824	-	cis-encoded	antisense: VP_RS04950
sRNA171	NC_004603.1	1065462	1065642	+	cis-encoded	antisense: VP_RS04960
sRNA172	NC_004603.1	1078201	1078364	+	cis-encoded	antisense: VP_RS05020
sRNA173	NC_004603.1	1079275	1079413	+	cis-encoded	antisense: VP_RS05025
sRNA174	NC_004603.1	1091192	1091418	+	cis-encoded	antisense: VP_RS05065
sRNA175	NC_004603.1	1091644	1091833	+	cis-encoded	antisense: VP_RS05065
sRNA176	NC_004603.1	1092976	1093189	+	cis-encoded	antisense: VP_RS05070
sRNA177	NC_004603.1	1099065	1097985	-	cis-encoded	antisense: VP_RS05095
sRNA178	NC_004603.1	1104430	1103946	-	cis-encoded	antisense: VP_RS05120

sRNA179	NC_004603.1	1104789	1104527	-	cis-encoded	antisense: VP_RS05120
sRNA180	NC_004603.1	1106325	1104849	-	cis-encoded	antisense: VP_RS05125 VP_RS05120
sRNA181	NC_004603.1	1106923	1106454	-	cis-encoded	antisense: VP_RS05135 VP_RS05130
sRNA182	NC_004603.1	1112575	1109852	-	cis-encoded	antisense: VP_RS05170 VP_RS05175 VP_RS05165 VP_RS05160
sRNA183	NC_004603.1	1126507	1127147	+	cis-encoded	antisense: VP_RS05245
sRNA184	NC_004603.1	1135176	1135001	-	cis-encoded	antisense: VP_RS05280
sRNA185	NC_004603.1	1148081	1147910	-	cis-encoded	antisense: VP_RS05320
sRNA186	NC_004603.1	1152910	1152738	-	cis-encoded	antisense: VP_RS05335
sRNA187	NC_004603.1	1160729	1161809	+	cis-encoded	antisense: VP_RS05370
sRNA188	NC_004603.1	1169302	1168657	-	cis-encoded	antisense: VP_RS05395
sRNA189	NC_004603.1	1177923	1177508	-	cis-encoded	antisense: VP_RS05440
sRNA190	NC_004603.1	1178291	1178023	-	cis-encoded	antisense: VP_RS05445
sRNA191	NC_004603.1	1184699	1185266	+	cis-encoded	antisense: VP_RS05485
sRNA192	NC_004603.1	1185433	1185822	+	cis-encoded	antisense: VP_RS05485
sRNA193	NC_004603.1	1189893	1189999	+	cis-encoded	antisense: VP_RS05510
sRNA194	NC_004603.1	1206773	1207195	+	cis-encoded	antisense: VP_RS05585
sRNA195	NC_004603.1	1222250	1222504	+	cis-encoded	antisense: VP_RS05655
sRNA196	NC_004603.1	1232293	1231627	-	cis-encoded	antisense: VP_RS05700 VP_RS05710 VP_RS05705
sRNA197	NC_004603.1	1239344	1240151	+	cis-encoded	antisense:

						VP_RS05740
sRNA198	NC_004603.1	1266578	1267007	+	cis-encoded	antisense: VP_RS05855
sRNA199	NC_004603.1	1267340	1267766	+	cis-encoded	antisense: VP_RS05860
sRNA200	NC_004603.1	1268013	1268694	+	cis-encoded	antisense: VP_RS05860
sRNA201	NC_004603.1	1272325	1272486	+	cis-encoded	antisense: VP_RS05880
sRNA202	NC_004603.1	1272709	1273141	+	cis-encoded	antisense: VP_RS05885
sRNA203	NC_004603.1	1273194	1273605	+	cis-encoded	antisense: VP_RS05890
sRNA204	NC_004603.1	1303862	1304086	+	cis-encoded	antisense: VP_RS06000
sRNA205	NC_004603.1	1306254	1306591	+	cis-encoded	antisense: VP_RS06010
sRNA206	NC_004603.1	1310277	1310769	+	cis-encoded	antisense: VP_RS06035 VP_RS06030
sRNA207	NC_004603.1	1311152	1311295	+	cis-encoded	antisense: VP_RS06035 VP_RS06040
sRNA208	NC_004603.1	1312922	1313111	+	cis-encoded	antisense: VP_RS06040
sRNA209	NC_004603.1	1316029	1315624	-	cis-encoded	antisense: VP_RS06050 VP_RS06055
sRNA210	NC_004603.1	1321180	1321756	+	cis-encoded	antisense: VP_RS06070
sRNA211	NC_004603.1	1327109	1328369	+	cis-encoded	antisense: VP_RS06095
sRNA212	NC_004603.1	1337791	1338310	+	cis-encoded	antisense: VP_RS06130
sRNA213	NC_004603.1	1349559	1349707	+	cis-encoded	antisense: VP_RS06180
sRNA214	NC_004603.1	1358517	1357535	-	cis-encoded	antisense: VP_RS06215
sRNA215	NC_004603.1	1359296	1359019	-	cis-encoded	antisense: VP_RS06215 VP_RS06220

sRNA216	NC_004603.1	1361095	1359391	-	cis-encoded	antisense: VP_RS06225 VP_RS06235 VP_RS06220 VP_RS06230
sRNA217	NC_004603.1	1374994	1375897	+	cis-encoded	antisense: VP_RS06290
sRNA218	NC_004603.1	1391361	1390717	-	cis-encoded	antisense: VP_RS06360
sRNA219	NC_004603.1	1404925	1405197	+	cis-encoded	antisense: VP_RS06440
sRNA220	NC_004603.1	1408410	1409780	+	cis-encoded	antisense: VP_RS06455
sRNA221	NC_004603.1	1410268	1410496	+	cis-encoded	antisense: VP_RS06460
sRNA222	NC_004603.1	1410601	1410906	+	cis-encoded	antisense: VP_RS06460
sRNA223	NC_004603.1	1415954	1412994	-	cis-encoded	antisense: VP_RS06485 VP_RS06475 VP_RS06470 VP_RS06480
sRNA224	NC_004603.1	1422424	1419908	-	cis-encoded	antisense: VP_RS06505
sRNA225	NC_004603.1	1422405	1426193	+	cis-encoded	antisense: VP_RS06515 VP_RS06510 VP_RS06520
sRNA226	NC_004603.1	1426277	1427090	+	cis-encoded	antisense: VP_RS06525
sRNA227	NC_004603.1	1427221	1427824	+	cis-encoded	antisense: VP_RS06530
sRNA228	NC_004603.1	1427927	1429871	+	cis-encoded	antisense: VP_RS06535 VP_RS06530
sRNA229	NC_004603.1	1436550	1432282	-	cis-encoded	antisense: VP_RS06555 VP_RS06565 VP_RS06545 VP_RS06550 VP_RS06560
sRNA230	NC_004603.1	1444703	1444408	-	cis-encoded	antisense: VP_RS06615
sRNA231	NC_004603.1	1492901	1493014	+	cis-encoded	antisense:

						VP_RS06765
sRNA232	NC_004603.1	1493269	1493365	+	cis-encoded	antisense: VP_RS06765
sRNA233	NC_004603.1	1494629	1494809	+	cis-encoded	antisense: VP_RS06765
sRNA234	NC_004603.1	1496687	1496187	-	cis-encoded	antisense: VP_RS06775 VP_RS06770
sRNA235	NC_004603.1	1507049	1506112	-	cis-encoded	antisense: VP_RS06810 VP_RS06805 VP_RS06815
sRNA236	NC_004603.1	1508680	1507102	-	cis-encoded	antisense: VP_RS06820 VP_RS06815
sRNA237	NC_004603.1	1516346	1516558	+	cis-encoded	antisense: VP_RS06845
sRNA238	NC_004603.1	1521207	1521154	-	cis-encoded	antisense: VP_RS06865
sRNA239	NC_004603.1	1529755	1529894	+	cis-encoded	antisense: VP_RS06915
sRNA240	NC_004603.1	1534672	1533782	-	cis-encoded	antisense: VP_RS06930
sRNA241	NC_004603.1	1567002	1566773	-	cis-encoded	antisense: VP_RS07055
sRNA242	NC_004603.1	1568036	1567644	-	cis-encoded	antisense: VP_RS07065
sRNA243	NC_004603.1	1594318	1594552	+	cis-encoded	antisense: VP_RS07180
sRNA244	NC_004603.1	1595447	1595344	-	cis-encoded	antisense: VP_RS07185
sRNA245	NC_004603.1	1597692	1597797	+	cis-encoded	antisense: VP_RS07200
sRNA246	NC_004603.1	1606733	1607045	+	cis-encoded	antisense: VP_RS07255
sRNA247	NC_004603.1	1608699	1609006	+	cis-encoded	antisense: VP_RS07270 VP_RS07265
sRNA248	NC_004603.1	1609264	1610510	+	cis-encoded	antisense: VP_RS07270 VP_RS07275
sRNA249	NC_004603.1	1616441	1616561	+	cis-encoded	antisense: VP_RS07295
sRNA250	NC_004603.1	1617003	1617065	+	cis-encoded	antisense:

						VP_RS07295
sRNA251	NC_004603.1	1618466	1618103	-	cis-encoded	antisense: VP_RS07305
sRNA252	NC_004603.1	1620075	1619246	-	cis-encoded	antisense: VP_RS07310
sRNA253	NC_004603.1	1621435	1620365	-	cis-encoded	antisense: VP_RS07315 VP_RS07310 VP_RS07320
sRNA254	NC_004603.1	1626862	1621703	-	cis-encoded	antisense: VP_RS07325 VP_RS07335 VP_RS07330 VP_RS07340
sRNA255	NC_004603.1	1637347	1637873	+	cis-encoded	antisense: VP_RS07380
sRNA256	NC_004603.1	1638438	1639184	+	cis-encoded	antisense: VP_RS07385
sRNA257	NC_004603.1	1639438	1639786	+	cis-encoded	antisense: VP_RS07390
sRNA258	NC_004603.1	1646162	1646850	+	cis-encoded	antisense: VP_RS07425
sRNA259	NC_004603.1	1647315	1647854	+	cis-encoded	antisense: VP_RS07430
sRNA260	NC_004603.1	1651853	1655052	+	cis-encoded	antisense: VP_RS07455 VP_RS07465 VP_RS07460 VP_RS07470
sRNA261	NC_004603.1	1663453	1659516	-	cis-encoded	antisense: VP_RS07505 VP_RS07515 VP_RS07500 VP_RS07525 VP_RS07510 VP_RS07520
sRNA262	NC_004603.1	1665249	1663507	-	cis-encoded	antisense: VP_RS23550 VP_RS07530
sRNA263	NC_004603.1	1684720	1684954	+	cis-encoded	antisense: VP_RS07680
sRNA264	NC_004603.1	1685598	1686287	+	cis-encoded	antisense: VP_RS07680
sRNA265	NC_004603.1	1690365	1690455	+	cis-encoded	antisense:

						VP_RS07695
sRNA266	NC_004603.1	1691310	1691692	+	cis-encoded	antisense: VP_RS07700
sRNA267	NC_004603.1	1692386	1692607	+	cis-encoded	antisense: VP_RS07705
sRNA268	NC_004603.1	1693277	1698089	+	cis-encoded	antisense: VP_RS07710
sRNA269	NC_004603.1	1702256	1703858	+	cis-encoded	antisense: VP_RS07730
sRNA270	NC_004603.1	1704158	1704364	+	cis-encoded	antisense: VP_RS07735 VP_RS07730
sRNA271	NC_004603.1	1704439	1704814	+	cis-encoded	antisense: VP_RS07735
sRNA272	NC_004603.1	1730663	1731008	+	cis-encoded	antisense: VP_RS07845
sRNA273	NC_004603.1	1731594	1731692	+	cis-encoded	antisense: VP_RS07845
sRNA274	NC_004603.1	1731892	1732378	+	cis-encoded	antisense: VP_RS07845
sRNA275	NC_004603.1	1732604	1733247	+	cis-encoded	antisense: VP_RS07845
sRNA276	NC_004603.1	1733342	1734706	+	cis-encoded	antisense: VP_RS07845
sRNA277	NC_004603.1	1737380	1736776	-	cis-encoded	antisense: VP_RS07850
sRNA278	NC_004603.1	1738130	1737448	-	cis-encoded	antisense: VP_RS07855
sRNA279	NC_004603.1	1760015	1760093	+	cis-encoded	antisense: VP_RS07915
sRNA280	NC_004603.1	1760630	1760872	+	cis-encoded	antisense: VP_RS07915
sRNA281	NC_004603.1	1761681	1761749	+	cis-encoded	antisense: VP_RS07920
sRNA282	NC_004603.1	1763286	1763518	+	cis-encoded	antisense: VP_RS07925
sRNA283	NC_004603.1	1763670	1763989	+	cis-encoded	antisense: VP_RS07925
sRNA284	NC_004603.1	1764197	1764463	+	cis-encoded	antisense: VP_RS07925
sRNA285	NC_004603.1	1765464	1766653	+	cis-encoded	antisense: VP_RS07930
sRNA286	NC_004603.1	1766862	1766933	+	cis-encoded	antisense: VP_RS07935

sRNA287	NC_004603.1	1766998	1768210	+	cis-encoded	antisense: VP_RS07935 VP_RS07940
sRNA288	NC_004603.1	1768638	1768876	+	cis-encoded	antisense: VP_RS07945
sRNA289	NC_004603.1	1812852	1814396	+	cis-encoded	antisense: VP_RS08205
sRNA290	NC_004603.1	1818770	1819007	+	cis-encoded	antisense: VP_RS08225
sRNA291	NC_004603.1	1819172	1820062	+	cis-encoded	antisense: VP_RS08225
sRNA292	NC_004603.1	1820299	1820601	+	cis-encoded	antisense: VP_RS08230
sRNA293	NC_004603.1	1820675	1821064	+	cis-encoded	antisense: VP_RS08235 VP_RS08230
sRNA294	NC_004603.1	1821211	1821489	+	cis-encoded	antisense: VP_RS08235
sRNA295	NC_004603.1	1826210	1826089	-	cis-encoded	antisense: VP_RS08255
sRNA296	NC_004603.1	1894422	1894665	+	cis-encoded	antisense: VP_RS08545 VP_RS08540
sRNA297	NC_004603.1	1954046	1954211	+	cis-encoded	antisense: VP_RS09115
sRNA298	NC_004603.1	1954753	1955003	+	cis-encoded	antisense: VP_RS09115
sRNA299	NC_004603.1	1956215	1956556	+	cis-encoded	antisense: VP_RS09125
sRNA300	NC_004603.1	1956753	1957256	+	cis-encoded	antisense: VP_RS09125
sRNA301	NC_004603.1	1960966	1961146	+	cis-encoded	antisense: VP_RS09140
sRNA302	NC_004603.1	1962958	1962707	-	cis-encoded	antisense: VP_RS09150
sRNA303	NC_004603.1	1964077	1963100	-	cis-encoded	antisense: VP_RS09150 VP_RS09155
sRNA304	NC_004603.1	1964701	1964565	-	cis-encoded	antisense: VP_RS09155
sRNA305	NC_004603.1	2005883	2006806	+	cis-encoded	antisense: VP_RS09330
sRNA306	NC_004603.1	2006893	2007142	+	cis-encoded	antisense: VP_RS09330

sRNA307	NC_004603.1	2016959	2018682	+	cis-encoded	antisense: VP_RS09385
sRNA308	NC_004603.1	2019008	2019354	+	cis-encoded	antisense: VP_RS09385
sRNA309	NC_004603.1	2029356	2029500	+	cis-encoded	antisense: VP_RS09430
sRNA310	NC_004603.1	2029563	2029950	+	cis-encoded	antisense: VP_RS09430
sRNA311	NC_004603.1	2030150	2030486	+	cis-encoded	antisense: VP_RS09430
sRNA312	NC_004603.1	2031783	2032112	+	cis-encoded	antisense: VP_RS09435
sRNA313	NC_004603.1	2037483	2037780	+	cis-encoded	antisense: VP_RS09470
sRNA314	NC_004603.1	2069873	2070248	+	cis-encoded	antisense: VP_RS09620
sRNA315	NC_004603.1	2070700	2070963	+	cis-encoded	antisense: VP_RS09620
sRNA316	NC_004603.1	2071437	2071766	+	cis-encoded	antisense: VP_RS09620
sRNA317	NC_004603.1	2086370	2086696	+	cis-encoded	antisense: VP_RS09675
sRNA318	NC_004603.1	2087151	2088181	+	cis-encoded	antisense: VP_RS09680
sRNA319	NC_004603.1	2107153	2106585	-	cis-encoded	antisense: VP_RS09775 VP_RS09780
sRNA320	NC_004603.1	2111369	2111093	-	cis-encoded	antisense: VP_RS09790
sRNA321	NC_004603.1	2112141	2111490	-	cis-encoded	antisense: VP_RS09790 VP_RS09795
sRNA322	NC_004603.1	2112714	2112343	-	cis-encoded	antisense: VP_RS09795
sRNA323	NC_004603.1	2113054	2112848	-	cis-encoded	antisense: VP_RS09795
sRNA324	NC_004603.1	2126764	2126969	+	cis-encoded	antisense: VP_RS09860
sRNA325	NC_004603.1	2127231	2128968	+	cis-encoded	antisense: VP_RS09865 VP_RS09870
sRNA326	NC_004603.1	2139310	2139484	+	cis-encoded	antisense: VP_RS09905
sRNA327	NC_004603.1	2142223	2142482	+	cis-encoded	antisense:

						VP_RS09915
sRNA328	NC_004603.1	2142702	2142947	+	cis-encoded	antisense: VP_RS09915 VP_RS09920
sRNA329	NC_004603.1	2143584	2143484	-	cis-encoded	antisense: VP_RS09925
sRNA330	NC_004603.1	2146796	2147191	+	cis-encoded	antisense: VP_RS09945
sRNA331	NC_004603.1	2147245	2147896	+	cis-encoded	antisense: VP_RS09945
sRNA332	NC_004603.1	2153125	2154243	+	cis-encoded	antisense: VP_RS09975 VP_RS09980
sRNA333	NC_004603.1	2154904	2155893	+	cis-encoded	antisense: VP_RS09985 VP_RS09990
sRNA334	NC_004603.1	2158316	2159008	+	cis-encoded	antisense: VP_RS10010 VP_RS10005
sRNA335	NC_004603.1	2161716	2161356	-	cis-encoded	antisense: VP_RS10025
sRNA336	NC_004603.1	2163160	2162312	-	cis-encoded	antisense: VP_RS10025
sRNA337	NC_004603.1	2169546	2169621	+	cis-encoded	antisense: VP_RS10045
sRNA338	NC_004603.1	2172783	2172485	-	cis-encoded	antisense: VP_RS10060
sRNA339	NC_004603.1	2183001	2183099	+	cis-encoded	antisense: VP_RS10100
sRNA340	NC_004603.1	2187826	2188071	+	cis-encoded	antisense: VP_RS10120
sRNA341	NC_004603.1	2196398	2196873	+	cis-encoded	antisense: VP_RS10155
sRNA342	NC_004603.1	2199277	2200987	+	cis-encoded	antisense: VP_RS10170
sRNA343	NC_004603.1	2222917	2223097	+	cis-encoded	antisense: VP_RS10290
sRNA344	NC_004603.1	2224548	2223709	-	cis-encoded	antisense: VP_RS10295 VP_RS10300
sRNA345	NC_004603.1	2225536	2225125	-	cis-encoded	antisense: VP_RS10305
sRNA346	NC_004603.1	2225754	2228406	+	cis-encoded	antisense: VP_RS10310

sRNA347	NC_004603.1	2230880	2231886	+	cis-encoded	antisense: VP_RS10325
sRNA348	NC_004603.1	2252070	2251852	-	cis-encoded	antisense: VP_RS10410
sRNA349	NC_004603.1	2254079	2253899	-	cis-encoded	antisense: VP_RS10415
sRNA350	NC_004603.1	2263270	2263526	+	cis-encoded	antisense: VP_RS10450
sRNA351	NC_004603.1	2270497	2269434	-	cis-encoded	antisense: VP_RS10485 VP_RS10480
sRNA352	NC_004603.1	2275258	2275010	-	cis-encoded	antisense: VP_RS10500
sRNA353	NC_004603.1	2291637	2291824	+	cis-encoded	antisense: VP_RS10595
sRNA354	NC_004603.1	2292862	2293221	+	cis-encoded	antisense: VP_RS10600
sRNA355	NC_004603.1	2294978	2295233	+	cis-encoded	antisense: VP_RS10610
sRNA356	NC_004603.1	2295557	2295906	+	cis-encoded	antisense: VP_RS10610
sRNA357	NC_004603.1	2298833	2299361	+	cis-encoded	antisense: VP_RS10630
sRNA358	NC_004603.1	2304324	2304509	+	cis-encoded	antisense: VP_RS10640
sRNA359	NC_004603.1	2305333	2305721	+	cis-encoded	antisense: VP_RS10645
sRNA360	NC_004603.1	2307259	2308170	+	cis-encoded	antisense: VP_RS10655
sRNA361	NC_004603.1	2314226	2314494	+	cis-encoded	antisense: VP_RS10685
sRNA362	NC_004603.1	2320558	2320775	+	cis-encoded	antisense: VP_RS10715
sRNA363	NC_004603.1	2321291	2321595	+	cis-encoded	antisense: VP_RS10715
sRNA364	NC_004603.1	2321689	2322343	+	cis-encoded	antisense: VP_RS10715
sRNA365	NC_004603.1	2322967	2323128	+	cis-encoded	antisense: VP_RS10720
sRNA366	NC_004603.1	2323337	2323843	+	cis-encoded	antisense: VP_RS10720
sRNA367	NC_004603.1	2327618	2327465	-	cis-encoded	antisense: VP_RS10735
sRNA368	NC_004603.1	2331933	2332012	+	cis-encoded	antisense:

						VP_RS10760
sRNA369	NC_004603.1	2338023	2338399	+	cis-encoded	antisense: VP_RS10800
sRNA370	NC_004603.1	2371015	2371418	+	cis-encoded	antisense: VP_RS10960 VP_RS10955
sRNA371	NC_004603.1	2371629	2372186	+	cis-encoded	antisense: VP_RS10960 VP_RS10965
sRNA372	NC_004603.1	2372581	2372881	+	cis-encoded	antisense: VP_RS10965
sRNA373	NC_004603.1	2376414	2376700	+	cis-encoded	antisense: VP_RS10980
sRNA374	NC_004603.1	2381627	2381856	+	cis-encoded	antisense: VP_RS11025
sRNA375	NC_004603.1	2382222	2382366	+	cis-encoded	antisense: VP_RS11025
sRNA376	NC_004603.1	2382471	2382719	+	cis-encoded	antisense: VP_RS11030
sRNA377	NC_004603.1	2392794	2393046	+	cis-encoded	antisense: VP_RS11085
sRNA378	NC_004603.1	2395349	2395574	+	cis-encoded	antisense: VP_RS11100
sRNA379	NC_004603.1	2411676	2411833	+	cis-encoded	antisense: VP_RS11175
sRNA380	NC_004603.1	2418386	2418626	+	cis-encoded	antisense: VP_RS11190
sRNA381	NC_004603.1	2419652	2419897	+	cis-encoded	antisense: VP_RS11200
sRNA382	NC_004603.1	2420088	2420301	+	cis-encoded	antisense: VP_RS11200
sRNA383	NC_004603.1	2420587	2420806	+	cis-encoded	antisense: VP_RS11205
sRNA384	NC_004603.1	2420984	2421451	+	cis-encoded	antisense: VP_RS11210
sRNA385	NC_004603.1	2421535	2421831	+	cis-encoded	antisense: VP_RS11210
sRNA386	NC_004603.1	2422339	2422623	+	cis-encoded	antisense: VP_RS11210
sRNA387	NC_004603.1	2427923	2428233	+	cis-encoded	antisense: VP_RS11235
sRNA388	NC_004603.1	2429314	2431059	+	cis-encoded	antisense: VP_RS11245 VP_RS11250

sRNA389	NC_004603.1	2432384	2432253	-	cis-encoded	antisense: VP_RS11260
sRNA390	NC_004603.1	2457604	2463391	+	cis-encoded	antisense: VP_RS11395 VP_RS11410 VP_RS11400 VP_RS11405 VP_RS11415 VP_RS11390
sRNA391	NC_004603.1	2475429	2475605	+	cis-encoded	antisense: VP_RS11490
sRNA392	NC_004603.1	2529801	2529888	+	cis-encoded	antisense: VP_RS11690
sRNA393	NC_004603.1	2540882	2541116	+	cis-encoded	antisense: VP_RS11750
sRNA394	NC_004603.1	2541270	2541501	+	cis-encoded	antisense: VP_RS11750 VP_RS23610
sRNA395	NC_004603.1	2546012	2548105	+	cis-encoded	antisense: VP_RS11775
sRNA396	NC_004603.1	2554014	2554587	+	cis-encoded	antisense: VP_RS11800
sRNA397	NC_004603.1	2555011	2555464	+	cis-encoded	antisense: VP_RS11805
sRNA398	NC_004603.1	2556419	2556943	+	cis-encoded	antisense: VP_RS11810
sRNA399	NC_004603.1	2557044	2557285	+	cis-encoded	antisense: VP_RS11810
sRNA400	NC_004603.1	2557562	2558341	+	cis-encoded	antisense: VP_RS11815
sRNA401	NC_004603.1	2558974	2560451	+	cis-encoded	antisense: VP_RS11820 VP_RS11825
sRNA402	NC_004603.1	2573163	2575335	+	cis-encoded	antisense: VP_RS11895
sRNA403	NC_004603.1	2575664	2575951	+	cis-encoded	antisense: VP_RS11900
sRNA404	NC_004603.1	2577708	2578028	+	cis-encoded	antisense: VP_RS11915
sRNA405	NC_004603.1	2578396	2579293	+	cis-encoded	antisense: VP_RS11915
sRNA406	NC_004603.1	2580548	2580904	+	cis-encoded	antisense: VP_RS11920
sRNA407	NC_004603.1	2581959	2582212	+	cis-encoded	antisense:

						VP_RS11925
sRNA408	NC_004603.1	2585664	2586865	+	cis-encoded	antisense: VP_RS11955
sRNA409	NC_004603.1	2587090	2587412	+	cis-encoded	antisense: VP_RS11955
sRNA410	NC_004603.1	2587465	2587682	+	cis-encoded	antisense: VP_RS11960 VP_RS11955
sRNA411	NC_004603.1	2591300	2589893	-	cis-encoded	antisense: VP_RS11980 VP_RS11975
sRNA412	NC_004603.1	2602254	2601749	-	cis-encoded	antisense: VP_RS12015
sRNA413	NC_004603.1	2602515	2602378	-	cis-encoded	antisense: VP_RS12015
sRNA414	NC_004603.1	2609973	2609870	-	cis-encoded	antisense: VP_RS12035
sRNA415	NC_004603.1	2611519	2610116	-	cis-encoded	antisense: VP_RS12035 VP_RS12040
sRNA416	NC_004603.1	2627081	2628016	+	cis-encoded	antisense: VP_RS12095
sRNA417	NC_004603.1	2630390	2633128	+	cis-encoded	antisense: VP_RS12115
sRNA418	NC_004603.1	2641936	2641644	-	cis-encoded	antisense: VP_RS12140
sRNA419	NC_004603.1	2654047	2655793	+	cis-encoded	antisense: VP_RS12210 VP_RS12205
sRNA420	NC_004603.1	2656906	2657377	+	cis-encoded	antisense: VP_RS12210
sRNA421	NC_004603.1	2657567	2657865	+	cis-encoded	antisense: VP_RS12215
sRNA422	NC_004603.1	2658145	2659939	+	cis-encoded	antisense: VP_RS12215
sRNA423	NC_004603.1	2660025	2660182	+	cis-encoded	antisense: VP_RS12215
sRNA424	NC_004603.1	2660240	2660386	+	cis-encoded	antisense: VP_RS12220
sRNA425	NC_004603.1	2669654	2671567	+	cis-encoded	antisense: VP_RS12275 VP_RS12285 VP_RS12280 VP_RS12270

sRNA426	NC_004603.1	2672490	2672550	+	cis-encoded	antisense: VP_RS12290
sRNA427	NC_004603.1	2672877	2673119	+	cis-encoded	antisense: VP_RS12290
sRNA428	NC_004603.1	2683577	2683822	+	cis-encoded	antisense: VP_RS12335
sRNA429	NC_004603.1	2684741	2684999	+	cis-encoded	antisense: VP_RS12340
sRNA430	NC_004603.1	2685153	2685755	+	cis-encoded	antisense: VP_RS12340
sRNA431	NC_004603.1	2685905	2686042	+	cis-encoded	antisense: VP_RS12340 VP_RS12345
sRNA432	NC_004603.1	2687944	2687998	+	cis-encoded	antisense: VP_RS12385 VP_RS12390
sRNA433	NC_004603.1	2688361	2688758	+	cis-encoded	antisense: VP_RS12395 VP_RS12400
sRNA434	NC_004603.1	2689355	2689663	+	cis-encoded	antisense: VP_RS12400
sRNA435	NC_004603.1	2693598	2693975	+	cis-encoded	antisense: VP_RS12415
sRNA436	NC_004603.1	2697799	2698759	+	cis-encoded	antisense: VP_RS12430
sRNA437	NC_004603.1	2698814	2699211	+	cis-encoded	antisense: VP_RS12435
sRNA438	NC_004603.1	2704462	2705681	+	cis-encoded	antisense: VP_RS12470
sRNA439	NC_004603.1	2706024	2706126	+	cis-encoded	antisense: VP_RS12475
sRNA440	NC_004603.1	2706448	2706674	+	cis-encoded	antisense: VP_RS12475
sRNA441	NC_004603.1	2706791	2707477	+	cis-encoded	antisense: VP_RS12475
sRNA442	NC_004603.1	2708808	2709052	+	cis-encoded	antisense: VP_RS12485
sRNA443	NC_004603.1	2716226	2716360	+	cis-encoded	antisense: VP_RS12505
sRNA444	NC_004603.1	2717572	2717818	+	cis-encoded	antisense: VP_RS12515
sRNA445	NC_004603.1	2718002	2718302	+	cis-encoded	antisense: VP_RS12520 VP_RS12515

sRNA446	NC_004603.1	2720803	2720874	+	cis-encoded	antisense: VP_RS12530
sRNA447	NC_004603.1	2722524	2723055	+	cis-encoded	antisense: VP_RS12540
sRNA448	NC_004603.1	2723113	2724539	+	cis-encoded	antisense: VP_RS12550 VP_RS12540 VP_RS12545
sRNA449	NC_004603.1	2726387	2726167	-	cis-encoded	antisense: VP_RS12555
sRNA450	NC_004603.1	2737154	2737664	+	cis-encoded	antisense: VP_RS12620
sRNA451	NC_004603.1	2740194	2740855	+	cis-encoded	antisense: VP_RS12640
sRNA452	NC_004603.1	2741479	2742496	+	cis-encoded	antisense: VP_RS12645
sRNA453	NC_004603.1	2742596	2743377	+	cis-encoded	antisense: VP_RS12650
sRNA454	NC_004603.1	2743453	2743818	+	cis-encoded	antisense: VP_RS12650
sRNA455	NC_004603.1	2748279	2750132	+	cis-encoded	antisense: VP_RS12670
sRNA456	NC_004603.1	2751390	2751005	-	cis-encoded	antisense: VP_RS12675
sRNA457	NC_004603.1	2762170	2761952	-	cis-encoded	antisense: VP_RS12745
sRNA458	NC_004603.1	2797847	2796320	-	cis-encoded	antisense: VP_RS12970 VP_RS12975
sRNA459	NC_004603.1	2805308	2804992	-	cis-encoded	antisense: VP_RS13005
sRNA460	NC_004603.1	2811658	2812403	+	cis-encoded	antisense: VP_RS13055 VP_RS13050
sRNA461	NC_004603.1	2812811	2812906	+	cis-encoded	antisense: VP_RS13060
sRNA462	NC_004603.1	2833520	2833050	-	cis-encoded	antisense: VP_RS13160
sRNA463	NC_004603.1	2844395	2844456	+	cis-encoded	antisense: VP_RS13200
sRNA464	NC_004603.1	2844561	2844614	+	cis-encoded	antisense: VP_RS13200
sRNA465	NC_004603.1	2844922	2845052	+	cis-encoded	antisense: VP_RS13200

sRNA466	NC_004603.1	2852404	2852930	+	cis-encoded	antisense: VP_RS13235
sRNA467	NC_004603.1	2864811	2865276	+	cis-encoded	antisense: VP_RS13290
sRNA468	NC_004603.1	2867674	2867195	-	cis-encoded	antisense: VP_RS13300 VP_RS13305
sRNA469	NC_004603.1	2872848	2873149	+	cis-encoded	antisense: VP_RS13315
sRNA470	NC_004603.1	2873432	2873617	+	cis-encoded	antisense: VP_RS13315
sRNA471	NC_004603.1	2896113	2897702	+	cis-encoded	antisense: VP_RS13410
sRNA472	NC_004603.1	2903282	2904722	+	cis-encoded	antisense: VP_RS13440 VP_RS13455 VP_RS13445 VP_RS13450
sRNA473	NC_004603.1	2905385	2905586	+	cis-encoded	antisense: VP_RS13460
sRNA474	NC_004603.1	2922150	2922492	+	cis-encoded	antisense: VP_RS13535
sRNA475	NC_004603.1	2922769	2922996	+	cis-encoded	antisense: VP_RS13535
sRNA476	NC_004603.1	2923924	2924150	+	cis-encoded	antisense: VP_RS13540
sRNA477	NC_004603.1	2936202	2936437	+	cis-encoded	antisense: VP_RS13585
sRNA478	NC_004603.1	2938542	2940574	+	cis-encoded	antisense: VP_RS13600 VP_RS13605
sRNA479	NC_004603.1	2940677	2940932	+	cis-encoded	antisense: VP_RS13605
sRNA480	NC_004603.1	2941087	2941447	+	cis-encoded	antisense: VP_RS13605
sRNA481	NC_004603.1	2941517	2941800	+	cis-encoded	antisense: VP_RS13605
sRNA482	NC_004603.1	2942127	2943096	+	cis-encoded	antisense: VP_RS13610 VP_RS13615
sRNA483	NC_004603.1	2948631	2948946	+	cis-encoded	antisense: VP_RS13660
sRNA484	NC_004603.1	2954537	2954340	-	cis-encoded	antisense: VP_RS13690

sRNA485	NC_004603.1	2957632	2960052	+	cis-encoded	antisense: VP_RS13710 VP_RS13715
sRNA486	NC_004603.1	2960115	2962478	+	cis-encoded	antisense: VP_RS13720 VP_RS13725
sRNA487	NC_004603.1	2967838	2968178	+	cis-encoded	antisense: VP_RS13745
sRNA488	NC_004603.1	2968286	2968366	+	cis-encoded	antisense: VP_RS13745
sRNA489	NC_004603.1	2971601	2971824	+	cis-encoded	antisense: VP_RS13760
sRNA490	NC_004603.1	2971890	2972118	+	cis-encoded	antisense: VP_RS13760
sRNA491	NC_004603.1	2975631	2976822	+	cis-encoded	antisense: VP_RS13780
sRNA492	NC_004603.1	2977597	2979302	+	cis-encoded	antisense: VP_RS13795 VP_RS13790
sRNA493	NC_004603.1	2980038	2980133	+	cis-encoded	antisense: VP_RS13800
sRNA494	NC_004603.1	2980690	2980769	+	cis-encoded	antisense: VP_RS13800
sRNA495	NC_004603.1	2980879	2981116	+	cis-encoded	antisense: VP_RS13805
sRNA496	NC_004603.1	2981191	2981830	+	cis-encoded	antisense: VP_RS13810
sRNA497	NC_004603.1	2989856	2989972	+	cis-encoded	antisense: VP_RS13885 VP_RS13890
sRNA498	NC_004603.1	2997948	2998225	+	cis-encoded	antisense: VP_RS13930
sRNA499	NC_004603.1	2998412	2999131	+	cis-encoded	antisense: VP_RS13930
sRNA500	NC_004603.1	2999207	2999393	+	cis-encoded	antisense: VP_RS13930
sRNA501	NC_004603.1	3000412	3000254	-	cis-encoded	antisense: VP_RS13935
sRNA502	NC_004603.1	3013361	3013567	+	cis-encoded	antisense: VP_RS14005
sRNA503	NC_004603.1	3017476	3017974	+	cis-encoded	antisense: VP_RS14025
sRNA504	NC_004603.1	3018881	3019160	+	cis-encoded	antisense: VP_RS14030

sRNA505	NC_004603.1	3022368	3022914	+	cis-encoded	antisense: VP_RS14045
sRNA506	NC_004603.1	3032684	3029612	-	cis-encoded	antisense: VP_RS14090 VP_RS14095 VP_RS14085
sRNA507	NC_004603.1	3040317	3037519	-	cis-encoded	antisense: VP_RS14125 VP_RS14115 VP_RS14120 VP_RS14110
sRNA508	NC_004603.1	3041614	3041125	-	cis-encoded	antisense: VP_RS14130
sRNA509	NC_004603.1	3090335	3090066	-	cis-encoded	antisense: VP_RS14325
sRNA510	NC_004603.1	3100324	3100485	+	cis-encoded	antisense: VP_RS14360
sRNA511	NC_004603.1	3105050	3105259	+	cis-encoded	antisense: VP_RS14385
sRNA512	NC_004603.1	3105853	3106174	+	cis-encoded	antisense: VP_RS14390
sRNA513	NC_004603.1	3106604	3106796	+	cis-encoded	antisense: VP_RS14395
sRNA514	NC_004603.1	3108723	3119593	+	cis-encoded	antisense: VP_RS14410 VP_RS14430 VP_RS14420 VP_RS14425 VP_RS14415 VP_RS14435
sRNA515	NC_004603.1	3119928	3120403	+	cis-encoded	antisense: VP_RS14445 VP_RS14440
sRNA516	NC_004603.1	3120966	3122436	+	cis-encoded	antisense: VP_RS14465 VP_RS14455 VP_RS14450 VP_RS14460
sRNA517	NC_004603.1	3144547	3144801	+	cis-encoded	antisense: VP_RS14560
sRNA518	NC_004603.1	3155793	3155981	+	cis-encoded	antisense: VP_RS14610
sRNA519	NC_004603.1	3157037	3157675	+	cis-encoded	antisense: VP_RS14620

sRNA520	NC_004603.1	3180837	3181125	+	cis-encoded	antisense: VP_RS14755
sRNA521	NC_004603.1	3182018	3182215	+	cis-encoded	antisense: VP_RS14760
sRNA522	NC_004603.1	3182879	3183047	+	cis-encoded	antisense: VP_RS14765
sRNA523	NC_004603.1	3183268	3183677	+	cis-encoded	antisense: VP_RS14770
sRNA524	NC_004603.1	3183959	3184499	+	cis-encoded	antisense: VP_RS14770
sRNA525	NC_004603.1	3186375	3186714	+	cis-encoded	antisense: VP_RS14785
sRNA526	NC_004603.1	3187541	3187744	+	cis-encoded	antisense: VP_RS14785
sRNA527	NC_004603.1	3221889	3222376	+	cis-encoded	antisense: VP_RS14945
sRNA528	NC_004603.1	3222644	3222787	+	cis-encoded	antisense: VP_RS14945
sRNA529	NC_004603.1	3245965	3246077	+	cis-encoded	antisense: VP_RS15070 VP_RS15075
sRNA530	NC_004603.1	3249670	3249515	-	cis-encoded	antisense: VP_RS15095
sRNA531	NC_004603.1	3256189	3256339	+	cis-encoded	antisense: VP_RS15125
sRNA532	NC_004603.1	3256404	3256613	+	cis-encoded	antisense: VP_RS15125
sRNA533	NC_004603.1	3257556	3257175	-	cis-encoded	antisense: VP_RS23630 VP_RS15130
sRNA534	NC_004605.1	24870	24544	-	cis-encoded	antisense: VP_RS15395
sRNA535	NC_004605.1	27069	27350	+	cis-encoded	antisense: VP_RS15405
sRNA536	NC_004605.1	89217	88767	-	cis-encoded	antisense: VP_RS15715
sRNA537	NC_004605.1	118485	117729	-	cis-encoded	antisense: VP_RS15860
sRNA538	NC_004605.1	118784	118581	-	cis-encoded	antisense: VP_RS15860
sRNA539	NC_004605.1	144620	144409	-	cis-encoded	antisense: VP_RS16000
sRNA540	NC_004605.1	159140	158707	-	cis-encoded	antisense: VP_RS16080

sRNA541	NC_004605.1	258527	258613	+	cis-encoded	antisense: VP_RS16495
sRNA542	NC_004605.1	263223	263876	+	cis-encoded	antisense: VP_RS16520
sRNA543	NC_004605.1	291860	291772	-	cis-encoded	antisense: VP_RS16670
sRNA544	NC_004605.1	318634	318818	+	cis-encoded	antisense: VP_RS16805
sRNA545	NC_004605.1	322694	322967	+	cis-encoded	antisense: VP_RS16835
sRNA546	NC_004605.1	332789	332998	+	cis-encoded	antisense: VP_RS16885
sRNA547	NC_004605.1	353636	354662	+	cis-encoded	antisense: VP_RS16970
sRNA548	NC_004605.1	361488	361595	+	cis-encoded	antisense: VP_RS16995
sRNA549	NC_004605.1	393905	394089	+	cis-encoded	antisense: VP_RS17135
sRNA550	NC_004605.1	465971	465477	-	cis-encoded	antisense: VP_RS17475
sRNA551	NC_004605.1	469544	467068	-	cis-encoded	antisense: VP_RS17490 VP_RS17480 VP_RS17485
sRNA552	NC_004605.1	471199	471042	-	cis-encoded	antisense: VP_RS17490
sRNA553	NC_004605.1	606416	607019	+	cis-encoded	antisense: VP_RS18105
sRNA554	NC_004605.1	620491	620783	+	cis-encoded	antisense: VP_RS18160
sRNA555	NC_004605.1	628943	628432	-	cis-encoded	antisense: VP_RS18190 VP_RS18195
sRNA556	NC_004605.1	632978	632599	-	cis-encoded	antisense: VP_RS18215
sRNA557	NC_004605.1	634541	633909	-	cis-encoded	antisense: VP_RS18220
sRNA558	NC_004605.1	636181	635833	-	cis-encoded	antisense: VP_RS18225 VP_RS18220
sRNA559	NC_004605.1	644487	644139	-	cis-encoded	antisense: VP_RS18265
sRNA560	NC_004605.1	650152	651706	+	cis-encoded	antisense: VP_RS18300

						VP_RS18295
sRNA561	NC_004605.1	652391	653247	+	cis-encoded	antisense: VP_RS18305
sRNA562	NC_004605.1	659807	659565	-	cis-encoded	antisense: VP_RS18340
sRNA563	NC_004605.1	685952	684448	-	cis-encoded	antisense: VP_RS18390
sRNA564	NC_004605.1	720603	720677	+	cis-encoded	antisense: VP_RS18525
sRNA565	NC_004605.1	738595	739045	+	cis-encoded	antisense: VP_RS18595
sRNA566	NC_004605.1	827117	827706	+	cis-encoded	antisense: VP_RS19010
sRNA567	NC_004605.1	828065	828515	+	cis-encoded	antisense: VP_RS19015
sRNA568	NC_004605.1	828712	829107	+	cis-encoded	antisense: VP_RS19015
sRNA569	NC_004605.1	829617	829862	+	cis-encoded	antisense: VP_RS19015
sRNA570	NC_004605.1	830283	831143	+	cis-encoded	antisense: VP_RS19015 VP_RS19020
sRNA571	NC_004605.1	837108	836811	-	cis-encoded	antisense: VP_RS19045
sRNA572	NC_004605.1	855586	856343	+	cis-encoded	antisense: VP_RS19125
sRNA573	NC_004605.1	856477	857079	+	cis-encoded	antisense: VP_RS19125
sRNA574	NC_004605.1	872575	873811	+	cis-encoded	antisense: VP_RS19175
sRNA575	NC_004605.1	877965	876515	-	cis-encoded	antisense: VP_RS19195 VP_RS19190
sRNA576	NC_004605.1	882251	879964	-	cis-encoded	antisense: VP_RS19205 VP_RS19210
sRNA577	NC_004605.1	1038185	1040199	+	cis-encoded	antisense: VP_RS19915 VP_RS19910
sRNA578	NC_004605.1	1060609	1060832	+	cis-encoded	antisense: VP_RS19995
sRNA579	NC_004605.1	1078201	1078372	+	cis-encoded	antisense: VP_RS20080

sRNA580	NC_004605.1	1079297	1079404	+	cis-encoded	antisense: VP_RS20090 VP_RS20085
sRNA581	NC_004605.1	1091198	1091403	+	cis-encoded	antisense: VP_RS20135
sRNA582	NC_004605.1	1093037	1093182	+	cis-encoded	antisense: VP_RS20145
sRNA583	NC_004605.1	1104422	1103950	-	cis-encoded	antisense: VP_RS20185
sRNA584	NC_004605.1	1104789	1104529	-	cis-encoded	antisense: VP_RS20185
sRNA585	NC_004605.1	1106302	1104868	-	cis-encoded	antisense: VP_RS20190 VP_RS20185
sRNA586	NC_004605.1	1106914	1106457	-	cis-encoded	antisense: VP_RS20190
sRNA587	NC_004605.1	1111403	1111012	-	cis-encoded	antisense: VP_RS20225 VP_RS20230
sRNA588	NC_004605.1	1152934	1152737	-	cis-encoded	antisense: VP_RS20400
sRNA589	NC_004605.1	1184699	1185271	+	cis-encoded	antisense: VP_RS20545
sRNA590	NC_004605.1	1185432	1185822	+	cis-encoded	antisense: VP_RS20545
sRNA591	NC_004605.1	1231977	1231628	-	cis-encoded	antisense: VP_RS20755
sRNA592	NC_004605.1	1232291	1232034	-	cis-encoded	antisense: VP_RS20755
sRNA593	NC_004605.1	1266578	1267007	+	cis-encoded	antisense: VP_RS20875 VP_RS20880
sRNA594	NC_004605.1	1267340	1267766	+	cis-encoded	antisense: VP_RS20880
sRNA595	NC_004605.1	1303862	1304080	+	cis-encoded	antisense: VP_RS21060
sRNA596	NC_004605.1	1324328	1322768	-	cis-encoded	antisense: VP_RS21150 VP_RS21145 VP_RS21140
sRNA597	NC_004605.1	1358530	1357534	-	cis-encoded	antisense: VP_RS21290 VP_RS21295
sRNA598	NC_004605.1	1359291	1359043	-	cis-encoded	antisense:

sRNA599	NC_004605.1	1361099	1359401	-	cis-encoded	VP_RS21300 antisense: VP_RS21305 VP_RS21300
sRNA600	NC_004605.1	1374996	1375892	+	cis-encoded	antisense: VP_RS21390 VP_RS21385
sRNA601	NC_004605.1	1408410	1409794	+	cis-encoded	antisense: VP_RS21555 VP_RS21565 VP_RS21560
sRNA602	NC_004605.1	1410268	1410542	+	cis-encoded	VP_RS21570 antisense: VP_RS21575
sRNA603	NC_004605.1	1410600	1410910	+	cis-encoded	antisense: VP_RS21575
sRNA604	NC_004605.1	1422405	1426214	+	cis-encoded	antisense: VP_RS21645 VP_RS21655 VP_RS21650
sRNA605	NC_004605.1	1426276	1427102	+	cis-encoded	VP_RS21660 antisense: VP_RS21665
sRNA606	NC_004605.1	1427221	1427829	+	cis-encoded	antisense: VP_RS21665
sRNA607	NC_004605.1	1427927	1429871	+	cis-encoded	antisense: VP_RS21665
sRNA608	NC_004605.1	1492901	1493015	+	cis-encoded	antisense: VP_RS21955
sRNA609	NC_004605.1	1493269	1493369	+	cis-encoded	antisense: VP_RS21955
sRNA610	NC_004605.1	1529756	1529876	+	cis-encoded	antisense: VP_RS22065
sRNA611	NC_004605.1	1594318	1594550	+	cis-encoded	antisense: VP_RS22305
sRNA612	NC_004605.1	1616441	1616563	+	cis-encoded	antisense: VP_RS22390
sRNA613	NC_004605.1	1621151	1620709	-	cis-encoded	antisense: VP_RS22405
sRNA614	NC_004605.1	1637348	1637863	+	cis-encoded	antisense: VP_RS22505 VP_RS22500
sRNA615	NC_004605.1	1638442	1639183	+	cis-encoded	antisense: VP_RS22505

sRNA616	NC_004605.1	1639438	1639782	+	cis-encoded	antisense: VP_RS22505 VP_RS22510
sRNA617	NC_004605.1	1691310	1691692	+	cis-encoded	antisense: VP_RS22785
sRNA618	NC_004605.1	1694168	1694284	+	cis-encoded	antisense: VP_RS22795
sRNA619	NC_004605.1	1760632	1760868	+	cis-encoded	antisense: VP_RS23040
sRNA620	NC_004605.1	1763286	1763515	+	cis-encoded	antisense: VP_RS23055
sRNA621	NC_004605.1	1763670	1763986	+	cis-encoded	antisense: VP_RS23055
sRNA622	NC_004605.1	1764197	1764458	+	cis-encoded	antisense: VP_RS23055
sRNA623	NC_004605.1	1768640	1768875	+	cis-encoded	antisense: VP_RS23070
sRNA624	NC_004605.1	1826211	1826088	-	cis-encoded	antisense: VP_RS23315
sRNA625	NC_004603.1	113063	112731	-	trans-encoded	
sRNA626	NC_004603.1	118781	118581	-	trans-encoded	
sRNA627	NC_004603.1	1335819	1335623	-	trans-encoded	
sRNA628	NC_004603.1	3138584	3138692	+	trans-encoded	
sRNA629	NC_004603.1	3216339	3215949	-	trans-encoded	
sRNA630	NC_004605.1	333306	333484	+	trans-encoded	
sRNA631	NC_004605.1	446096	446041	-	trans-encoded	
sRNA632	NC_004605.1	476538	476482	-	trans-encoded	
sRNA633	NC_004605.1	953806	953700	-	trans-encoded	
sRNA634	NC_004605.1	1316032	1315623	-	trans-encoded	
sRNA635	NC_004605.1	1414466	1414412	-	trans-encoded	
sRNA636	NC_004605.1	1695595	1695705	+	trans-encoded	
